# Problem solving stages in the five square problem

**DOI:** 10.3389/fpsyg.2015.01050

**Published:** 2015-08-04

**Authors:** Anna Fedor, Eörs Szathmáry, Michael Öllinger

**Affiliations:** ^1^Parmenides Center for the Study of ThinkingPullach, Germany; ^2^Parmenides Center for the Conceptual Foundations of SciencePullach, Germany; ^3^MTA-ELTE Theoretical Biology and Evolutionary Ecology Research Group, Biological Institute, Eötvös Loránd UniversityBudapest, Hungary; ^4^Psychological Department, Ludwig-Maximilians-UniversityMunich, Germany

**Keywords:** insight, problem solving stages, impasse, four-stage model, Five-Square problem

## Abstract

According to the restructuring hypothesis, insight problem solving typically progresses through consecutive stages of search, impasse, insight, and search again for someone, who solves the task. The order of these stages was determined through self-reports of problem solvers and has never been verified behaviorally. We asked whether individual analysis of problem solving attempts of participants revealed the same order of problem solving stages as defined by the theory and whether their subjective feelings corresponded to the problem solving stages they were in. Our participants tried to solve the Five-Square problem in an online task, while we recorded the time and trajectory of their stick movements. After the task they were asked about their feelings related to insight and some of them also had the possibility of reporting impasse while working on the task. We found that the majority of participants did not follow the classic four-stage model of insight, but had more complex sequences of problem solving stages, with search and impasse recurring several times. This means that the classic four-stage model is not sufficient to describe variability on the individual level. We revised the classic model and we provide a new model that can generate all sequences found. Solvers reported insight more often than non-solvers and non-solvers reported impasse more often than solvers, as expected; but participants did not report impasse more often during behaviorally defined impasse stages than during other stages. This shows that impasse reports might be unreliable indicators of impasse. Our study highlights the importance of individual analysis of problem solving behavior to verify insight theory.

## Introduction

### Insight tasks and the restructuring hypothesis

**Figure 1:**
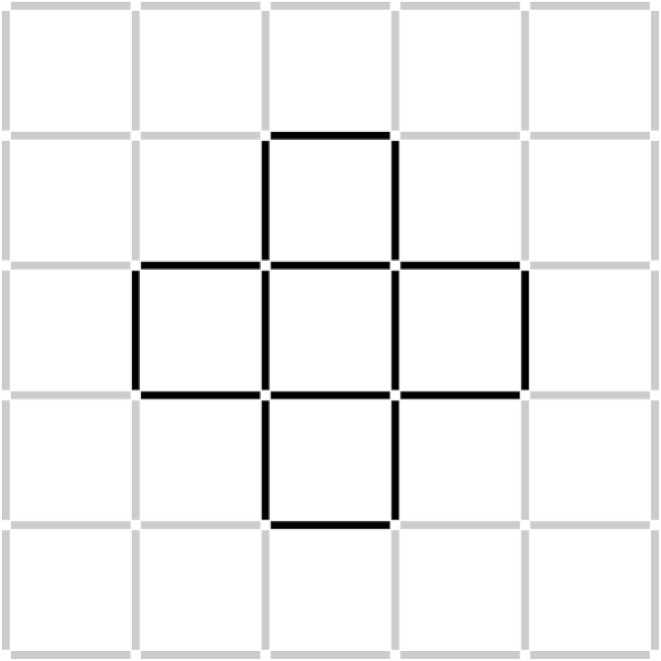
**The initial position of sticks on the grid in the five square problem**. For the solution, see the Supplementary Material. We show a 5-by-5 grid here, but in the computerized task, the cross shape was in the middle of a 9-by-9 grid.

Insight tasks are used in cognitive psychology to study insight problem solving (Öllinger and Knoblich, 2009). An example is the Five-Square problem (Katona, 1940), where problem solvers see a cross shape made of matchsticks (Figure 1) and they have to replace three matchsticks in order to get a shape of four squares of equal size instead of the given five squares in the cross shape. According to the restructuring hypothesis, insight problem solving is different from analytic problem solving (Fleck and Weisberg, 2013): problem solvers cannot assess how far they are from the solution (Metcalfe and Wiebe, 1987), and the solution pops into the problem solvers' mind suddenly and unexpectedly, evoking an Eureka moment, or “Aha!” experience (Durso et al., 1994; Wegner, 2002). This moment of enlightenment is usually—according to some, necessarily (Ohlsson, 1992; Knoblich et al., 1999, 2001; Jones, 2003; Öllinger et al., 2014a)—preceded by a longer period of impasse when the problem solver gets stuck and has no idea how to proceed.

The restructuring hypothesis explains these phenomena by a need for structural change in the mental representation of the problem (Wertheimer, 1959; Ohlsson, 1984a,b, 1992; Fleck and Weisberg, 2013). When the problem solver starts working on the task, the candidate solutions that the problem solver considers are unnecessarily constrained by some false assumptions about the task. The search space in which the person is looking for the solution does not contain the solution, because it is overly restricted. For example, in the Five-Square problem, most problem solvers start placing sticks to positions that touch original stick positions, i.e., they do not consider putting sticks further away from the initial shape. According to Öllinger et al. (2014a), they are constrained by the false assumption that the shape should stay in one piece. After failing several times, the problem solver reaches an impasse and feels that there is no way to get closer to the solution (Ohlsson, 1992). During an impasse problem solvers either do nothing or they repeat previous attempts to solve the task (Beeftink et al., 2008). Those people who do not solve the task get stuck in this state, while others overcome the self-imposed constraints. The latter start to look for the solution in an extended search space, in which, eventually they find the solution (in the Five-Square problem, they start putting sticks to further positions, and decompose the initial cross shape). Overcoming constraints and restructuring the task in one's mind is often accompanied by an “Aha!” experience that is usually used as the defining feature of insight problem solving (Bowden and Beeman, 1998; Boden, 2004; Bowden et al., 2005; Kounios et al., 2006; Danek et al., 2013, 2014).

### Levels of description of problem solving stages

The reader might have realized that in the description above, problem solving stages are sometimes described by the cognitive process that supposedly goes on in the problem solver's mind (Wallas, 1926), other times by what the problem solver does or feels (Danek et al., 2014). We see a lack of clarity in this regard in the literature, so here we would like to disentangle the different levels of description, and identify which phenomena belong to the cognitive, behavioral and affective level (Table 1).

**Table 1:** **Three levels of description of insight problem solving stages for a successful problem solver who goes through restructuring**.

**Level of description**	**Stage 1: constrained search**	**Stage 2: impasse**	**Stage 3: insight**	**Stage 4: extended search**	**Stage 5: solution**
Cognitive level (what goes on in the problem solver's mind)	Conscious search in the initial, constrained search space (constraints, heuristics)	Unconscious search; incubation; fixation; parallel search	Representational change: the self-imposed constraints on the search space are relaxed	Conscious search in the new, extended search space	Finding the solution
Behavioral level (what the problem solver does)	The problem solver repeatedly tries to solve the problem (makes moves)	The problem solver is inactive or the problem solver repeats his previous moves	The problem solver makes the critical move: the first move outside the constrained search space	The problem solver repeatedly tries to solve the problem (makes moves)	The problem solver solves the problem
Affective level (what the problem solver feels)	Determination, motivation	The problem solver feels stuck, frustrated and does not know how to proceed	Insight, “Aha!” experience, Eureka moment	Excitement	Success, satisfaction

The first stage is most often described on the behavioral level: the problem solver repeatedly attempts to solve the task, but fails. The underlying cognitive process is supposed to be conscious search in the initial, constrained search space (e.g., MacGregor et al., 2001). The second stage, impasse, is usually identified by mental states: frustration, feeling being stuck, not knowing how to proceed (Ohlsson, 1992; Danek et al., 2014). People who cannot solve the task get stuck in this stage. Its behavioral correlates are either inactivity, or repeating previous moves or candidate solutions (Ohlsson, 1992; Jones, 2003; Beeftink et al., 2008). We do not know what happens during impasse on the cognitive level but we hypothesize that there is an underlying unconscious search process that could result in lifting the constraints of the initial search space (Metcalfe and Wiebe, 1987; Bowers et al., 1990; Seifert et al., 1994; Bowden and Beeman, 1998; Kounios and Beeman, 2014). According to others, incubation is important in this process (Seifert et al., 1994; Beeftink et al., 2008): taking breaks from the task increases solution rate. Recently, Dietrich and Haider (2014) and Fernando et al. (2010) proposed that the underlying search might be evolutionary and parallel in nature. That means that several search processes are launched at the same time and their results are tested against a criterion of success (fitness function). The most promising candidates are copied, and modified until a solution is found or a dead-end is reached. The third stage, insight, is named after its affective correlate, but its most important feature is the cognitive process of representational change, or restructuring (Knoblich et al., 1999). Its behavioral correlate (or rather causal effect) is sometimes identified as the critical move—the first move that goes outside the restricted search space (Jones, 2003). The fourth stage is conscious search again, but now, in the extended search space, at the end of which the problem solver finds the solution (Öllinger et al., 2014b). The fourth stage could be very brief (depending on the problem) because problem solvers often find the solution shortly after representational change, if they have the right insight. Probably because of this, it is sometimes unclear whether the “Aha!” experience is associated with restructuring or with finding the solution. Note that once the problem solver gets into the impasse, representational change is necessary, but not sufficient for solving the problem: some problem solvers cannot solve the task even when they are told to relax the constraint (Weisberg and Alba, 1982; Kershaw et al., 2013). On the other hand, it is possible to solve insight tasks without going through impasse and insight, if the first search space is sufficient.

The cognitive level is the most interesting for us, but since it is hidden, we can only observe the behavioral and emotional correlates of cognitive processes. The affective level cannot be directly assessed, because we either have to rely on subjective reports of problem solvers about their feelings or we could measure correlates of their emotions (e.g., heart rate, galvanic skin response, piloerection, pupil dilation). The behavioral level can be described easily and objectively. Behavioral measures during insight problem solving usually involve the moves that problem solvers make in order to solve the task. The difficulty is to get enough data from participants and to convince them to act out their thoughts instead of trying to solve the problem in their heads.

### Models: the order of problem solving stages

**Figure 2:**
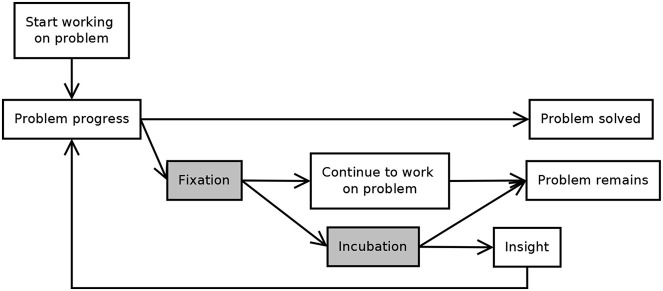
**Beeftink et al.'s (2008) stage model of insight problem solving (modified figure)**. Gray areas are part of impasse.

**Figure 3:**
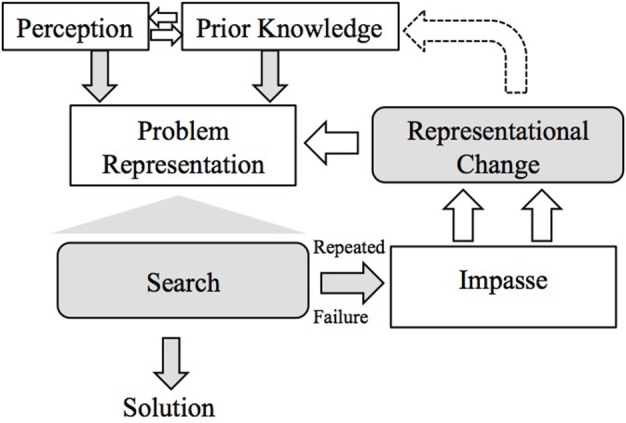
**Öllinger et al. (2014b) stage model of insight problem solving (original figure)**.

Wallas (1926) described the stages of insight problem solving as preparation, incubation, illumination, and verification. These roughly correspond to the stages described above. More recently, researchers constructed box-and-arrow type models, which show the possible sequences of problem solving stages (see Figures 2, 3). These models usually agree that the sequence of stages is different for people who solve the task with insight—who go through all the stages described above—from those who solve the task without insight; and that people who fail to solve the task get stuck in the impasse stage.

For example, based on Beeftink et al.'s (2008) model (Figure 2), the sequence of stages could be:

- Search → solve for people, who solve the task without impasse and insight,
- Search → fixation → search for people who can't solve the task, and
- Search → fixation → incubation → insight → solve for people who solve the task with restructuring.

In their model, fixation is an unconscious state, where people keep repeating previous problem solving attempts and incubation happens when the problem solver is in the conscious state of impasse: after unsuccessful attempts accumulate and reach a threshold, problem solvers realize that they are stuck and they take a break. Their model emphasizes the difference between routes with and without incubation: although it is possible that one cannot solve the task even after incubation, but it is not possible to solve the task without it.

Öllinger et al.'s (2014b) model (Ohlsson, 1992; based on Jones, 2003) does not differentiate between fixation and incubation, but these are implicitly included in the impasse stage. Their model generates search → solution, and search → impasse → representational change → search → solution sequences (Figure 3).

### Search space in the five-square problem

The search space of the Five-Square problem consists of the possible positions where the matchsticks could be placed (Simon and Newell, 1974). In our version of the task, this space was discretized: sticks could reside only on 180 predefined grid positions. The search space limits the number of possible stick movements, but it is probably still much larger than the actual search space of problem solvers—the collection of grid positions that they consider for a solution—because they rarely put sticks on the outer positions of the grid.

In a previous study, Öllinger et al. (2014a) found that the self-imposed constraint in this task is to keep the original shape in one piece, so we hypothesized that participants would first only use grid positions close to the original shape. They would quickly exhaust this search space and start repeating previously explored grid positions with their stick movements. Problem solvers who overcome this constraint expand their search space. The behavioral correlate should be the selection of new grid positions—including those that are part of the solution—further away from the cross shape.

### The present study

We propose an objective behavioral measure to define problem solving stages individually. We aimed to validate the stage models of insight by testing their predictions against this data. We were also interested how participants' subjective feelings correlate with their behavior.

We used the number of explored grid positions as a behavioral measure and tracked its changes through the problem solving attempts of participants. The grid positions explored by problem solvers are taken as instances from their cognitive search space and as such provide us with a window to their cognitive processes. Based on the stage model of insight, we predicted that in the case of problem solvers, who solve the task through restructuring, the number of explored grid positions would increase with every move at the beginning, until it reaches a plateau. The plateau indicates that the participant exhausted the possibilities within the constrained representation of the problem, and with every consecutive move the participant just repeats previous source and target positions. Participants could repeat positions even before exhausting the possibilities if they forget their previous moves. Some participants would give up, and stay inactive for longer periods of time. Both inactivity, and repetitions are taken as behavioral correlates of impasse (Beeftink et al., 2008), i.e., they can be used to objectively—independently of the problem solvers' feelings—identify the impasse stage. Some participants would get stuck in this stage, while others would get an insight and continue searching again. The search of an individual who had an insight is unconstrained by the idea that the shape should stay in one piece, so the person could put sticks further away from the original figure, and the number of explored grid positions would increase again until the person finds the solution. To sum up, we defined problem solving stages based on the curve of the number of explored grid positions through time for each participant individually and predicted that the sequence of problem solving stages would follow the process models of insight.

We also asked participants about their feelings related to insight and impasse. In our study, we used two self-reporting measures: an online impasse report and a *post-hoc* insight report. We asked participants in the impasse monitoring group to press a button when they felt being stuck during the task. After finishing the task, we asked all participants whether they had an “Aha!” experience (Danek et al., 2014). We predicted that more solvers would report insight than non-solvers, because successful solvers can have an “Aha!” experience, non-solvers probably cannot. We also predicted that non-solvers would report impasse more often than solvers, because all non-solvers should have an impasse, while solvers do not have it necessarily (it is possible to solve the task without impasse). We predicted that participants would report impasse during periods of frequent repetitions and inactivity, i.e., during the behaviorally defined impasse stage. According to Kounios and Beeman (2014) having an impasse is not necessary for having an “Aha!” experience—we wanted to see whether this claim holds for the five square problem too.

## Methods

### The task and design

In the Five-Square problem (Katona, 1940), the task is to reduce the number of five squares seen on Figure 1 to four squares. Participants should move three sticks to new grid positions without discarding any sticks (see solution in the Supplementary Material, Data Sheet 2).

We introduced two experimental groups. In the impasse monitoring group participants were provided with a description of the impasse feeling before they started the task, and they were asked to press a button when they felt like that during the task. A second group served as a control group to see whether the metacognitive task of monitoring one's impasse state had an impact on problem solving success.

### Participants

#### Recruitment

Four participants were recruited at Queen Mary University, London; the rest of the participants were recruited on the Internet, via a crowdsourcing platform called CrowdFlower. Here, people willing to work online can sign up and then choose from the available jobs that employers offer. We recruited “level 3 workers,” who are the highest rated, most trustworthy group of workers on CrowdFlower. They received one dollar as a payment. The participants were randomly assigned to either the impasse monitoring group or the control group.

Our experiments obey the Declaration of Helsinki (2013). We followed the code and the ethical principles of the German Psychological Society and the European Commission. Participants were allowed to quit the online experiment any time without providing a reason.

#### Sample size

We had more than twice as many participants in the impasse monitoring group than in the control group because we wanted to analyse the impasse monitoring group in more detail, namely perform a paired *t*-test on those participants who pressed the impasse button. We computed the required sample size for this test *a priori* with the G^*^ Power software (Faul et al., 2007), assuming a medium effect size (0.5) and setting α = 0.05 and power = 0.8. According to the analysis, the sample size for a matched-pairs *t*-test with these parameters should be 34, which we multiplied by 2.5 thinking that about half of the participants won't be able to solve the task and not all of these will press the impasse button. At the end, this analysis proved to be insufficient, since we assumed normal distribution, but the data was not normally distributed, so we had to do a non-parametric test instead of the planned paired *t*-test.

#### Exclusion criteria

We excluded all participants who closed the software before either they solved the task or the 15 min elapsed, even if they restarted the software afterwards. We did this, because we could not monitor their behavior while the program was closed.

#### Demographics

After excluding participants who closed the software early, 129 participants remained; 42 in the control group and 87 in the impasse monitoring group. There were 27 females and 102 males. The average age of participants was 30.3 years (range 16–69). We had participants from 37 countries and five continents (Europe = 59, Asia = 44, North-America = 9, South-America = 12, Africa = 5). The solvers and non-solvers were almost equally distributed across continents. Only four countries had more than five participants (India: 20, USA: 9, Romania: 7, UK: 6). Most of our participants had some higher education. Eleven participants had basic levels of English, the rest judged their English as intermediate level or higher.

### Materials and procedures

We provided a downloadable version of the Five-Square problem, written in Microsoft Visual Basic®, on the Internet. The program was run individually on the participants' computers and guided them through the experiment autonomously.

The program started with a practice trial, where participants had to drag and drop four sticks on the screen to four different grid positions. After completing this task participants received the following written instruction:

“*You will see five squares made of sticks on the screen. Your task is to move exactly three sticks to produce four squares of equal size, while leaving no sticks that are not part of squares*.

After three moves the task resets automatically or you can reset it anytime by pressing the Reset button. You can try to solve the task as many times as you like, in fact, try to show us all your ideas by moving the sticks, don't just try to solve the task in your head. You will have 15 min to solve the task—try to use all of it. It is not a problem, if you cannot solve the task, but if you close the program before 15 min elapsed, your submission will be invalid.”

Participants in the impasse-monitoring group received an additional instruction:

“*Sometimes before solving a difficult problem people feel like they are stuck, they are not getting closer to the solution. We would like to know if you feel like this during the task, so in this case please press the ‘I’m stuck' button. You can press the button more than once if you feel that this feeling increases.”*

After receiving the instructions, the initial figure of five squares appeared on the screen and participants could move the sticks by drag-and-drop with the mouse. The program recorded the movement of the sticks and the button presses (Impasse button and Restart button) along with the time that passed since starting the task. It has automatically checked whether a correct solution was achieved.

Participants were allowed to restart from the initial configuration of sticks as often as they wanted by pressing the “Restart” button. Alternatively, the program reset automatically after unsuccessful attempts of three moves and provided feedback (“*This is not a correct solution. Please, try again!*”). After solving the problem or reaching the upper time limit of 15 min, participants were asked to post the output log file to the experimenter via our website and to complete a short online questionnaire (about their age, gender, handedness, vision, educational background, nationality, mother tongue, and level of English). All participants were asked whether they solved the problem with or without insight:

“*Some people feel a sudden, unexpected, unintended, and surprising moment where a solution pops into someone's mind. The accompanying experience is often called ‘Aha!’ experience. Did you have this feeling before or when you solved the task?”*

The participants had to choose between the following answers: I did not solve the task; No, I did not feel anything like this; Yes, I felt exactly like this; Other: [free text].

### Analyses of problem solving stages

**Figure 4:**
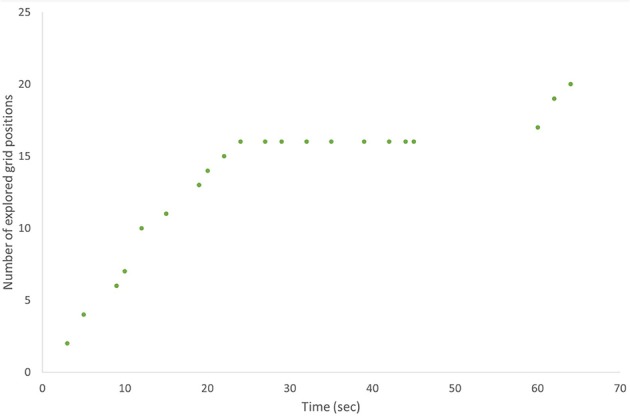
**The number of explored grid positions vs. time, cartoon figure with made up data**. This is what we expected the performance of a typical solver would look like. Each data point on these plots represents a stick movement. Slopes, plateaus, and gaps (see definitions in the Methods section) are identified and demarcated by black vertical lines.

We plotted the number of explored grid positions vs. time for each participant individually (see Figures 4, **6** and the Supplementary Material). On these plots, each point represents a move (dragging-and-dropping one stick). The number of explored grid positions includes the source positions (where the stick was picked up from) and the target positions (where the stick was put down to). With each move, it either increases by two (if the move consisted of new source and target positions), increases by one (if either the source position or the target position is a position that has already been used—i.e., if the move involves a repetition of a grid position), or stays constant (if both the source and the target position are repetitions).

For the purposes of our analyses, we defined three different patterns on these plots:

- A *slope* consists of minimum three consecutive moves that continuously increase the number of explored grid positions. This means that each move within the slope includes at least one new grid position that the participant has not used before.
- A *plateau* consists of minimum three consecutive moves that do not increase the number of explored grid positions. Moves within the plateau consist of repetitions of previously used grid positions.
- A *gap* is a period of time between two moves if it is longer than the average time between two consecutive moves for the participant + 2^*^SD. In case of first moves of trials, the time between the move and the last move of the previous trial includes the time spent by restarting the task, because the figure was visible during restarting.

On the behavioral level, these patterns translate to *search, repetition* and *inactivity*, respectively, which we hypothesize to indicate the cognitive phases of search (slope) and impasse (plateau or gap). We identified slopes, plateaus and gaps in the plots of each participant and looked at whether they conformed to the sequence of problem solving stages generated by the box-and-arrow models of insight problem solving. Figure 4 shows a made up example: it summarizes our predictions about the behavior of a typical solver conforming to current models of insight problem solving.

## Results

### Effect of manipulation

To control whether the additional metacognitive task in the impasse monitoring group affected problem solving performance, we compared the two groups in terms of solution rate and solution time.

#### Solution rate

The overall solution rate was 51%; 55% in the control group and 49% in the impasse monitoring group (Table 2). According to the Chi-square test, the difference was not significant: χ^2^_(1, *N* = 129)_ = 0.14, *p* = 0.70, Φ = 0.03.

**Table 2:** **Solution rate in the two experimental groups**.

**Participants**	**Control group**	**Impasse monitoring group**	**Total**
Solvers	23	43	66
Non-solvers	19	44	63
Total	42	87	129

#### Solution time

Twenty nine participants solved the task under 1 min in the two groups. The rest of the solution times were more or less evenly distributed between 2 and 15 min. We compared the solution time of solvers in the control group and the impasse monitoring group with a Mann-Whitney *U*-test—the difference between the groups was not significant, *U* = 424.00, *p* = 0.34, *r* = 0.12. For non-solvers, the task always ended after 15 min in both groups.

Since we did not find differences between the groups, we merged them for the rest of the analyses, except when we looked at the impasse monitoring group separately for the subjective impasse feeling analysis.

### Exclusion criteria for data analysis

There were 28 participants who only had one trial. Twenty six of these solved the task under 32 s; one solved it in 3.4 min and another one did not solve it. It is probable that the former solved the task in his head and the latter gave up after only one trial, and those 26 participants, who solved it in about half minute either already knew the task or just found the solution instantly. We excluded these 28 participants from the rest of the analyses, because we do not have enough movement data from them and also because we suspect that they knew the solution in advance.

One hundred and one participants remained, 34 in the control group (15 solvers) and 67 in the impasse monitoring group (24 solvers). The overall solution rate decreased to 39%.

### Target positions

**Figure 5:**
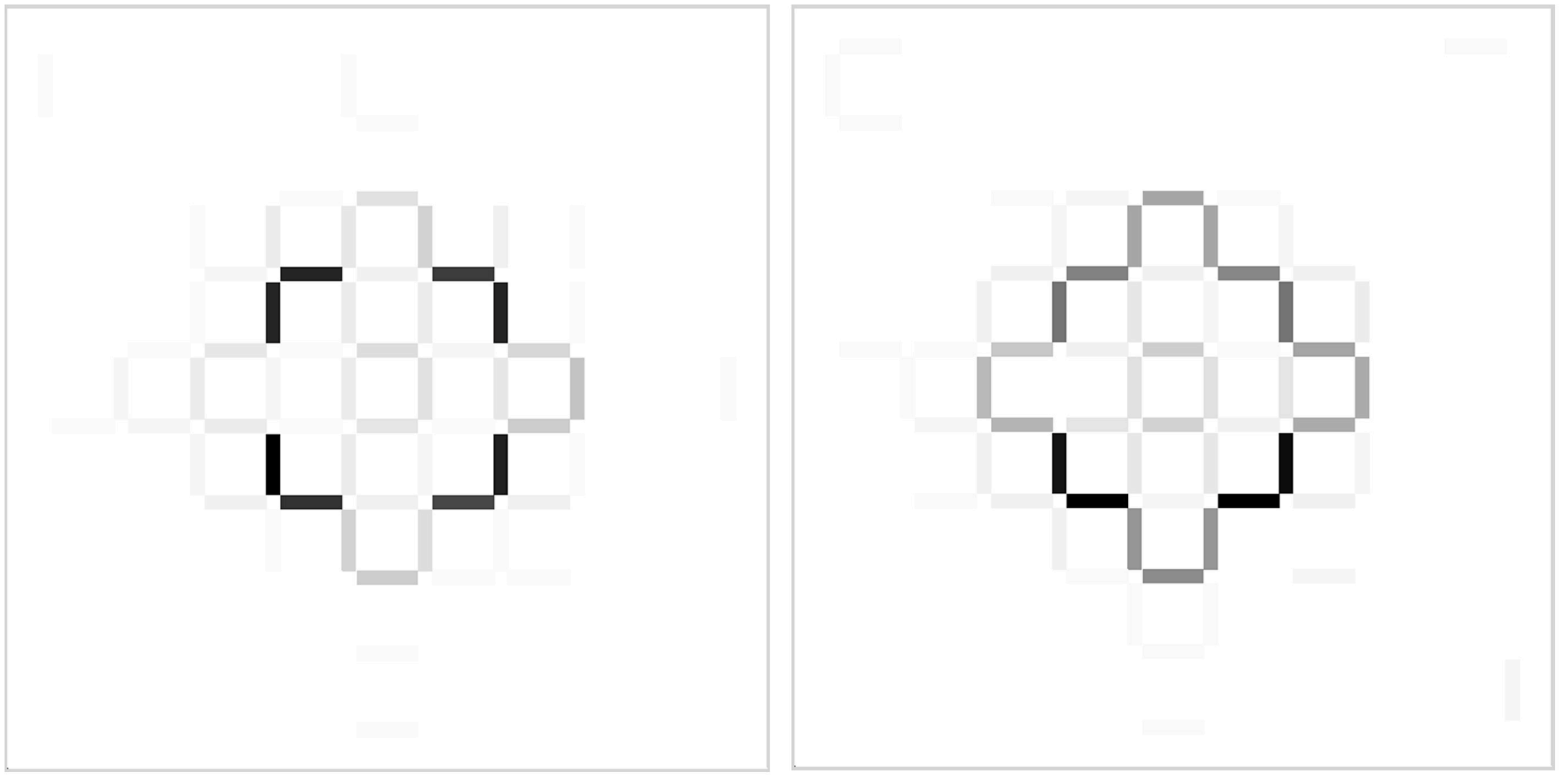
**The frequency of grid positions used as target positions in the moves of solvers in the first quarter of their moves (left) and the last quarter of their moves (right)**. The shades of grid positions represent a heat map: the darker the shade, the more frequently the position was chosen. Invisible positions were not used at all.

To illustrate how the actual search space of participants changed over time we plotted the grid with hues proportional to the relative frequency of positions used as targets (Figure 5)—for calculating the relative frequencies we used the first half of moves of each participant for the plot on the left and the second half of moves of each participant for the plot on the right.

We can see that the most frequent positions were the ones closest to the original cross shape (those that complemented the cross shape to a three-by-three square); the frequency of these eight positions (drawn in black in Figure 5 because they had the highest frequency) decreased over time: *M* = 51.4, *SD* = 3.6 in the first half of moves, and *M* = 38.4, *SD* = 9.9 in the second half of moves, *t*_(7)_ = 3.6, *p* = 0.009, *d* = 2.7032. On the other hand, the frequency of those 12 positions that could be part of the solution increased over time: *M* = 9.8, *SD* = 3.9 in the first half of moves, and *M* = 19.9, *SD* = 3.6 in the second half of moves, *t*_(11)_ = 9.6, *p* < 0.0001, *d* = 5.8029.

### Repetition rate

We compared the proportion of repeated positions of solvers and non-solvers. According to the unpaired *t*-test, the repetition rate of non-solvers (*M* = 0.5, *SD* = 0.2) was significantly higher than that of solvers (*M* = 0.3, *SD* = 0.3), *t*_(99)_ = 5.13, *p* < 0.0001, *d* = 1.03.

### Problem solving stages

**Figure 6:**
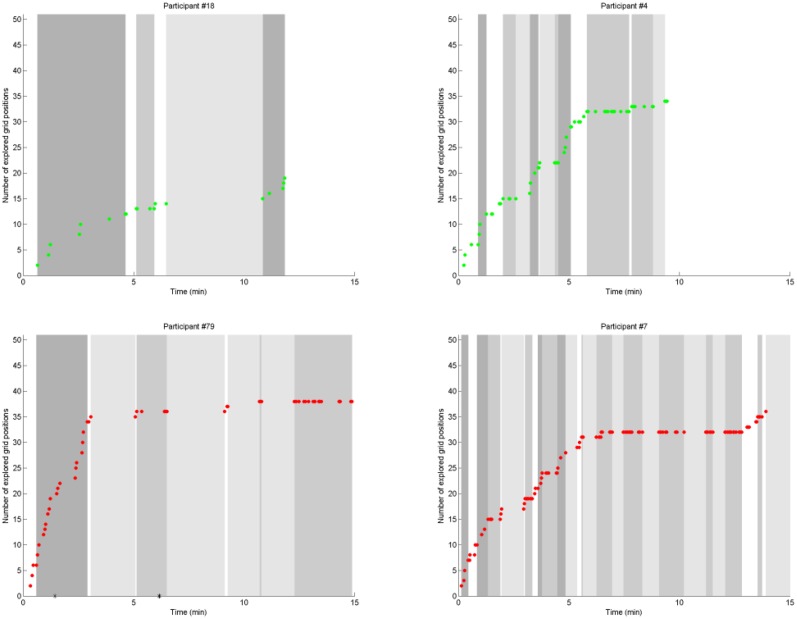
**The number of explored grid positions vs. time**. This figure shows data from four participants (two solvers, two non-solvers). The plots of all the participants can be found in the Supplementary Data. Examples plotted in green (#18 and #4) are from solvers, examples plotted in red (#79 and #7) are from non-solvers. Examples #18 and #79 conform to the stage model of insight, whereas examples #4 and #7 reveal more complex sequences of problem solving stages. Each data point on these plots represents a stick movement. Slopes, plateaus, and gaps (see definitions in the Methods section) are highlighted with different shades of gray: slopes are dark gray, plateaus are medium, and gaps are light gray. Asterisks on the horizontal axis indicate the time of impasse button presses in case of participant #79 (the rest of the participants in this figure were in the control group).

We looked at the number of explored grid positions through time for each participant (see the figures in the Supplementary Material and four examples in Figure 6) and we identified slopes, plateaus, and gaps, as defined in the Methods section. There were uncategorized moves that did not fall into neither slopes, nor plateaus—for the sake of simplicity, we ignored these. For 11 solvers and 6 non-solvers there was a gap before the first move—we disregarded these gaps too, because probably these were not signs of impasse.

All participants had at least one slope. The gap was missing in case of 5 solvers and 1 non-solver and the plateau was missing in case of 16 solvers and 5 non-solvers. Table 3 shows the different sequences, their interpretation and the number of participants who had such a sequence. We categorized the sequences based on whether they conformed to the stage model of insight problem solving.

**Table 3:** **Sequences of problem solving stages, their interpretation and the number of participants who had such a sequence**.

	**Sequence of problem solving stages**	**Interpretation**	**Number of participants**	**Conforms to the stage model of insight?**	**Sum**
**Solvers**	Slope	Solved the task without impasse	8	Yes	19 solvers (49%)
	Slope—alternating gaps and plateaus—slope	Classic search—impasse—search sequence, with probably an insight before the second search stage	7	Yes	
	Slope—alternating gaps and plateaus	Classic search—impasse—search sequence but the moves leading to the solution were already used before	4	Yes	
	Other sequences with alternating slopes, gaps and plateaus	Recurrent search and impasse stages	20	No	20 solvers (51%)
**Non-solvers**	Slope—alternating gaps and plateaus	Search—impasse	7	Yes	8 non-solvers (13%)
	Plateau—slope—alternating gaps and plateaus	Search—impasse	1	Yes	
	Slope	Search (probably gave up before reaching impasse)	1	No	54 non-solvers (87%)
	Other sequences with alternating slopes, gaps and plateaus	Recurrent search and impasse stages	53	No	

*The last column on the right summarizes how many solvers and non-solvers conformed (gray background) or did not conform (white background) to the stage model of insight*.

We have found that 49% (19 out of 39) of the solvers and 13% (8 out of 62) of the non-solvers followed the classic stage model of insight. The rest of the participants had more complex sequences, with search and impasse stages recurring several times.

### Subjective feelings of insight and impasse

#### Subjective insight, impasse, and success

In the two groups, 74% of the solvers (29 participants out of 39 solvers) and 10% of the non-solvers (6 participants out of 62 non-solvers) reported insight feeling after the task. According to the Chi-square test with Yates correction, the association between having an insight and being a solver was significant, χ^2^_(1, *N* = 101)_ = 41.42, *p* < 0.0001, Φ = 0.64.

In the impasse monitoring group 33% of the solvers (8 participants out of 24 solvers) and 79% of the non-solvers (34 participants out of 43 non-solvers) pressed the impasse button at least once. According to the Chi-square test with Yates correction, the association between pressing the impasse button and being a non-solver was significant,χ^2^_(1, *N* = 67)_ = 11.89, *p* < 0.0006, Φ = 0.42.

#### Temporal distribution of impasse button presses

We tested whether participants in the impasse monitoring group tended to press the impasse button during plateaus and gaps or during slopes. There were 42 participants who pressed the button at least once—only these participants were included in the following two analyses.

We calculated the number of impasse button presses per minute during plateaus and gaps together and during slopes. According to the Wilcoxon matched-pairs signed-ranks test, the medians did not differ significantly, *Z* = 0.63, *p* = 0.53, *r* = 0.10.

This is quite unexpected so we checked whether non-solvers tended to press the impasse button more often during the second half of the task (7.5–15 min) than during the first half of the task (0–7.5 min). According to the paired *t*-test, the difference was not significant, *t*_(33)_ = 0.46, *p* = 0.65, *d* = 0.16.

#### Association of plateaus and gaps with reported “Aha!” experience

We compared the length of plateaus and gaps of solvers, who reported insight and solvers, who did not report insight. According to the unpaired *t*-test the difference was not significant, *t*_(37)_ = 1.04, *p* = 0.31, *d* = 0.3403. In fact, there were three solvers, who reported insight and did not have any plateaus or gaps, just a short slope.

## Discussion and conclusions

### Summary of results

We investigated problem solving stages in the Five-Square problem based on the main assumptions of the restructuring hypothesis of insight, i.e., that problem solving proceeds in different stages, including conscious search, impasse, and restructuring. We used a behavioral measure, the time and trajectory of stick movements by participants, to objectively define problem solving stages. This was based on the assumptions that during search the problem solver explores new grid positions, while during impasse, the problem solver either repeats previously explored grid positions or stays inactive. We analyzed how these stages follow each other and how they correlate with subjective impasse and insight feelings of the participants.

We have found that less than half of the successful problem solvers, and 13% of the unsuccessful participants followed the sequence of problem solving stages that the stage models of insight predicted. The rest of the participants had more complicated sequences of search and impasse, with these stages alternating several times. This means that the stage models must be extended to explain variability on the individual level. Most of this variability disappears in group-level analysis, which highlights the importance of individual analysis of problem solving behavior.

Solvers reported insight feeling more often than non-solvers and non-solvers reported impasse feeling more often than solvers, as it was expected. We predicted that the behaviorally defined impasse would correlate with the subjective feelings of participants, but we did not find temporal correlation between objective impasse stages and subjective impasse reports. This shows that impasse reports might be unreliable indicators of impasse.

### Empirical validation of problem solving stages

Although it has long been proposed that the process of insight problem solving can be divided into stages (Ohlsson, 1992; Sandkühler and Bhattacharya, 2008), there were very few studies that tried to empirically validate this hypothesis.

Knoblich et al. (2001) asked participants to solve matchstick arithmetic tasks while recording their eye-movements. They divided the problem solving time of each participant in three equally long intervals and compared mean fixation durations across intervals. They found that mean fixation duration increased across intervals on average, which they took as a sign that more and more participants entered impasse with time. This finding is based on the assumption that impasse is associated with longer fixations, because people tend to stare blankly at the screen when they don't know what to do. They also found that people tend to fixate on different elements of the task in each interval: in the first interval their attention is differentially allocated on elements that are consistent with the constrained search space induced by prior knowledge, while in the second and third intervals successful solvers look more at the elements of the extended search space. These findings seem to support the search-impasse sequence predicted by stage models of insight, however, the results were based on group level analyses, which mask individual variation.

Jones (2003) criticized that Knoblich et al. (2001) determined problem solving stages *post-hoc*. He examined move durations and eye-movements during two versions of the car park problem. He defined impasse as a longer break before the critical (insightful) move, i.e., in his definition, impasse is a time interval between two moves. This longer thinking time before the critical move also coincided with longer fixation times. In Jones's study there is only one impasse per definition, moreover, he also assumed that participants did not make moves during impasse, which we find problematic.

Fleck and Weisberg (2013) analyzed insight problem solving by thinking-aloud protocols (Ericsson and Simon, 1993). They found only weak evidence for the “classic impasse-restructuring-insight sequence” (p. 436), instead, participants used a variety of strategies. E.g., some participants solved the problem by using prior knowledge that they gathered from the solution of similar problems. Other participants solved the problem by the application of heuristics that helped them to restrict the search space. At last, some participants restructured their problem representation after they failed several times. Only these participants supposedly went through the problem solving stages described by the restructuring hypothesis.

### A new model for insight problem solving

Our main finding—that in most cases problem solving stages do not follow the simple stage model of insight—highlights the need for revising the model. The model should be able to explain recurring search and insight stages. There are different versions of the model, but all include search, impasse and restructuring/insight, but none of them can generate the variety of the sequences of these stages that we found. For example, Beeftink et al.'s (2008) model could be extended with loops to be able to account for various problem solving types. Their model already includes the possibility to go back to search behavior (“work on a problem”) once more after the impasse (Start working on a problem → Problem progress → Fixation → Continue to work on problem), but it does not allow to go back more than once or to go back and forth between fixation (repetitions) and incubation (inactivity). Öllinger et al.'s (2014b) model could also be modified to allow for a more flexible sequence of stages by making the arrow between search and impasse double headed.

**Figure 7:**
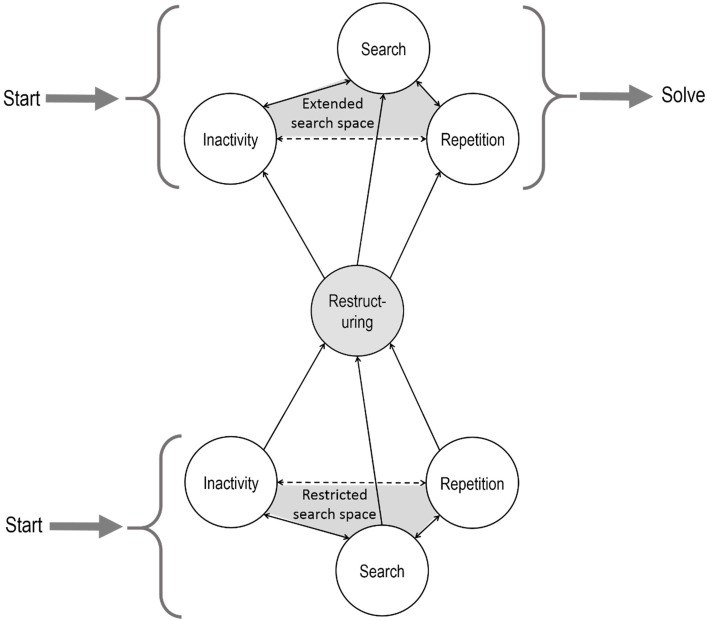
**Box-and-arrow model of insight problem solving**. White circles include problem solving stages described on the behavioral level. Gray shading refers to the cognitive level. Repetition and inactivity are sub-stages of impasse. Problem solving can either start in the restricted search space and then continue in the extended search space after restructuring, or could start in the extended search space right away.

We provide our own version of the model in Figure 7. It emphasizes that both before restructuring and after restructuring search, repetitions, and inactivity could follow each other in any order: each stage is accessible from the other two stages. Our model clearly separates the behavioral and the cognitive level, emphasizing the difference between objective observations and theory.

Box-and-arrow type stage models explain the order of problem solving stages, and the underlying theory of representational change explains—to some extent—what drives the process through these stages. Repetitions and inactivity on the cognitive level are supposedly caused by the confined search space: when there is a finite number of candidate solutions, after trying enough of them, the problem solver inevitably bumps into repeating previous ones, unless the problem solver takes a break. We found that non-solvers have a higher repetition rate than solvers which could be the result of either a smaller search space, more time spent in impasse or poorer working memory. Restructuring leads to an extended search space, which includes more candidate solutions and among them, the solution, and thus repetitions and inactivity is less frequent than in the restricted search space.

### Limitations

#### Impasse button presses

There has been a long tradition of identifying different problem solving stages by self-reports or thinking aloud protocols (Duncker and Lees, 1945; Schooler et al., 1993; Jung-Beeman et al., 2004; Danek et al., 2013; Fleck and Weisberg, 2013), but most often, self-reports about the “Aha!” experience are used only to differentiate between insight and non-insight solutions (Jung-Beeman et al., 2004). To our knowledge, there were no studies so far that tried to find an association between the time of impasse reports and some other, objectively defined impasse stage.

We did not find a temporal correlation between the objectively defined impasse and the impasse reports of participants. There are several possibilities why this happened so. The link between the objectively defined impasse stage and the impasse reports of participants has several elements: the cognitive impasse state causes repetitions and inactivity on the behavioral level that we used to objectively define impasse. The same cognitive state might cause the subjective feeling of being stuck which should remind the participant to press the impasse button.

It is possible that the subjective feelings of participants do not correlate with their cognitive state of impasse, i.e., they are not aware that they are in an impasse. Another possibility is that the subjective feeling does not correlate with the button presses: either because our instructions were unclear about the impasse, or because subjects left the computer during some of the gaps and thus were not in the position to press the button.

Of course, it is also possible that repetition and inactivity are not reliable signals of impasse or that the definitions we used to operationalize them were ineffective. In the literature impasse has a double definition: on the one hand it is defined behaviorally as repetitions and/or inactivity (Knoblich et al., 2001; Jones, 2003; Beeftink et al., 2008); on the other hand it is defined as a feeling of being stuck (Ohlsson, 1992). We believe that it is better to start with the objective behavioral definition than with the subjective definition based on feelings. In our data, subjective impasse reports are equally likely to occur at the beginning, middle or end of the problem solving time—they do not show any obvious pattern or aggregation. The other problem is that they are quite infrequent so it would be hard to define stages based on them. But most importantly, we believe that stick movements are better reflections of the cognitive processes going on in the problem solver's mind than his feelings, let alone button presses supposedly based on his feelings. Monitoring one's feelings is a parallel task, besides the main task of problem solving and the problem solver most likely concentrates more on solving the task than analysing and reporting his feelings.

We should also explain the counter-intuitive finding that 6 of the non-solvers reported having an “Aha!” experience. There could be several reasons for that. It is possible that participants did not understand our instructions properly, or that these participants had a kind of partial or wrong insight (Öllinger and Knoblich, 2009). It is also possible that the “Aha!” experience is not linked to representational change (Danek et al., 2014).

#### Online task

Our overall solution rate was a bit higher than expected (54%), but more importantly, we had several participants who solved the task very quickly. This could raise suspicion about the reliability of the online task: after all, without supervision from the experimenter, participants could look up the solution of the task online, or they could seek help from someone else. To decrease this possibility, we excluded participants who solved the task in one trial and kept only those who generated more data. The solution rate after the exclusion was 39%, which is comparable to previous studies (Öllinger et al., 2014a).

No supervision also meant that participants could act more freely, and that they were in a familiar setting (probably their homes), which allowed them to take a break whenever they wanted. In laboratory experiments participants probably feel pressure from the experimenter to move sticks at a constant pace, which could result in loosing inactivity as a measure of impasse in a laboratory setting.

Using an online crowdsourcing platform, such as CrowdFlower also helped us to recruit participants with a variety of backgrounds. Research in cognitive psychology suffers from the homogeneity of participant populations: between 2003 and 2007, about 80% of participants in psychological experiments were undergraduate students (Henrich et al., 2010). This WEIRD population (Western Educated Industrialized Rich Democratic) might be the least representative for all of humanity, and in some areas it might even be the outlier (Henrich et al., 2010). Our methods represent one step toward involving participants of different ages, nationalities, and with different educational backgrounds, enabling us to draw more general conclusions about humans.

#### Definition of problem solving stages

There seems to be a consensus in the literature that inactivity and repetitions are behavioral correlates of impasse, and that during the search stage the problem solver makes new moves, so we used gaps, plateaus and slopes as the behavioral signals of different stages of problem solving. Our definitions of slope and plateau are arbitrary to some extent: we set three moves as the minimum length of these stages. Setting them shorter would have made the sequence of stages intractable, while setting them longer would have resulted in ignoring too many transient moves. For gaps, we used the most common outlier criterion, i.e., longer than the average move time plus two SDs. Here, the balance is also between identifying too many or too few gaps.

As can be seen in Figure 1, the initial configuration of sticks was symmetric. Consequently, there were four different sites where a solution could occur and the symmetric moves were equivalent. For our analyses, we decided not to take into consideration the symmetry of the figure and when we counted the number of explored grid positions, we did not collapse equivalent positions into the same category. We did this, because it turned out that participants did not use all segments of the figure equivalently: the top left portion was used less often than the rest.

### Future research

Future research should include adapting our methods of defining problem solving stages in other insight problems. The nine dot problem seems to be a good candidate, because it is also a multistep problem with a discretized search space where participants make relatively many moves.

Another interesting direction would be to try to find the neural correlates of problem solving stages and thus to add the neural level of description to the narrative. So far, most brain imaging studies tried to find the neural correlates of insight (Dietrich and Kanso, 2010; Dietrich and Haider, 2014; Kounios and Beeman, 2014), but now that we can pinpoint the beginning and the end of all problem solving stages we could look at the difference between brain activity during all the different stages of problem solving.

The restructuring hypothesis does not explain the process that generates the candidate hypotheses during conscious search and the process that leads to restructuring. Bayesian models explain how hypotheses (candidate solutions) are selected but most of them take the initial population of hypotheses as a given, except some notable exceptions (Kemp and Tenenbaum, 2008). A process generating candidate hypotheses seems to be a must. A question is how a mental search in the hypothesis space is conducted. It remains to be seen whether an evolutionary search (as in Darwinian neurodynamics, Fernando et al., 2012) could ultimately explain the observations; this we shall consider in a subsequent paper.

To sum up, we pioneered a new method for finding problem solving stages in the five square problem based on solely behavioral data. The analysis of problem solving stages revealed a discrepancy between existing stage models of insight problem solving and our data, which highlights the need for revising the models and to further investigate problem solving stages on the individual level.

### Conflict of interest statement

The authors declare that the research was conducted in the absence of any commercial or financial relationships that could be construed as a potential conflict of interest.

## Supplementary material

Presentation 1.zip: Figures for all the participants like the ones shown in Figure 6 (The number of explored grid positions vs. time).

Presentation 2.zip: Animation of participants' stick movements. The videos show the moves in order, one move per second - the time of moves is not shown, because it would make the videos too long to watch.

Data Sheet 1.xlsx: Raw data file. This file contains all data that we collected from participants during problem solving and through the questionnaires.

Data Sheet 2.PDF: A possible solution of the task (three other rotations are also possible).
