# Supplementary figures and images for: Problem solving stages in the five square problem (part 2 of 2)
Source: Front Psychol. 2015 Aug 4;6:1050. doi: 10.3389/fpsyg.2015.01050 (PMC4523725; doi:10.3389/fpsyg.2015.01050)

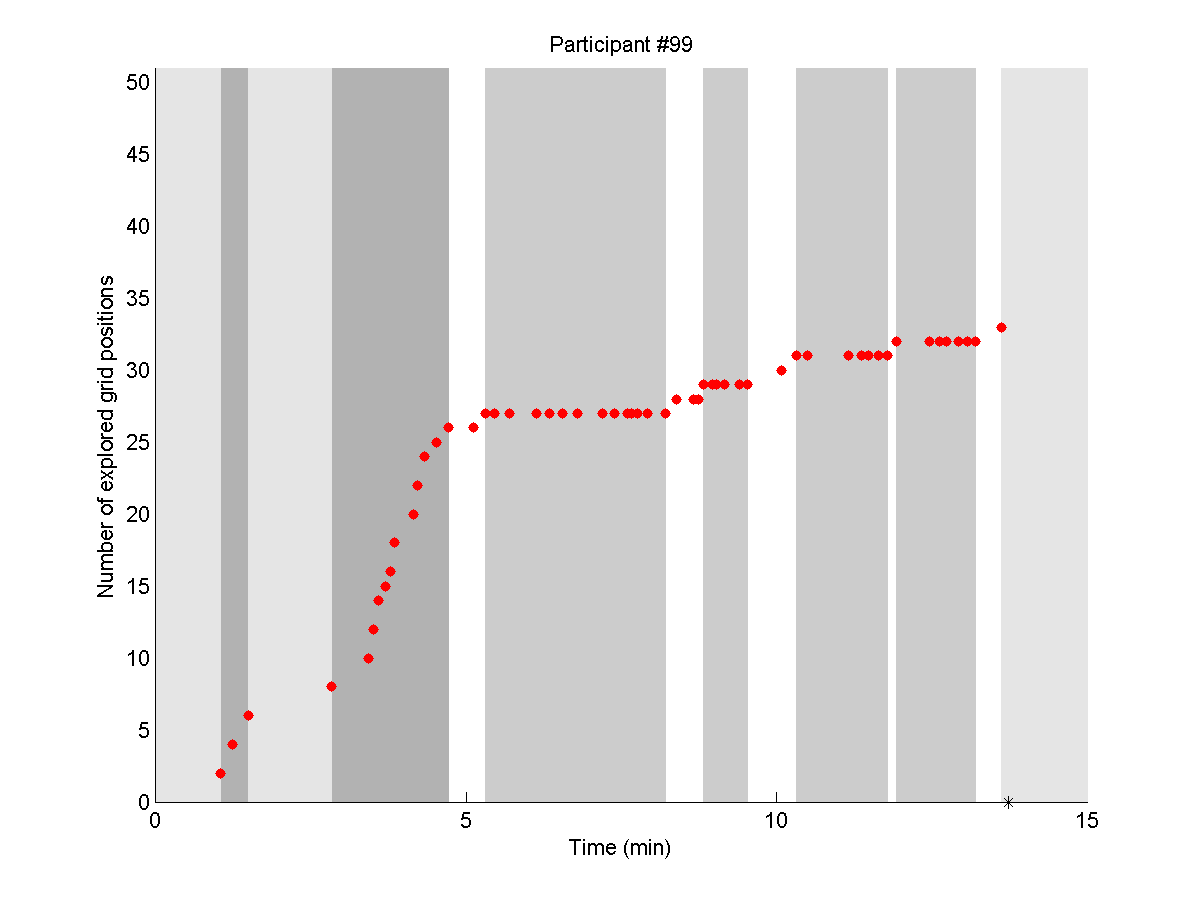

Supplement: Supplementary file 1 [file Presentation1.ZIP › individual plots/99.png]

## APPENDIX

---

A possible solution of the task (three other rotations are also possible):

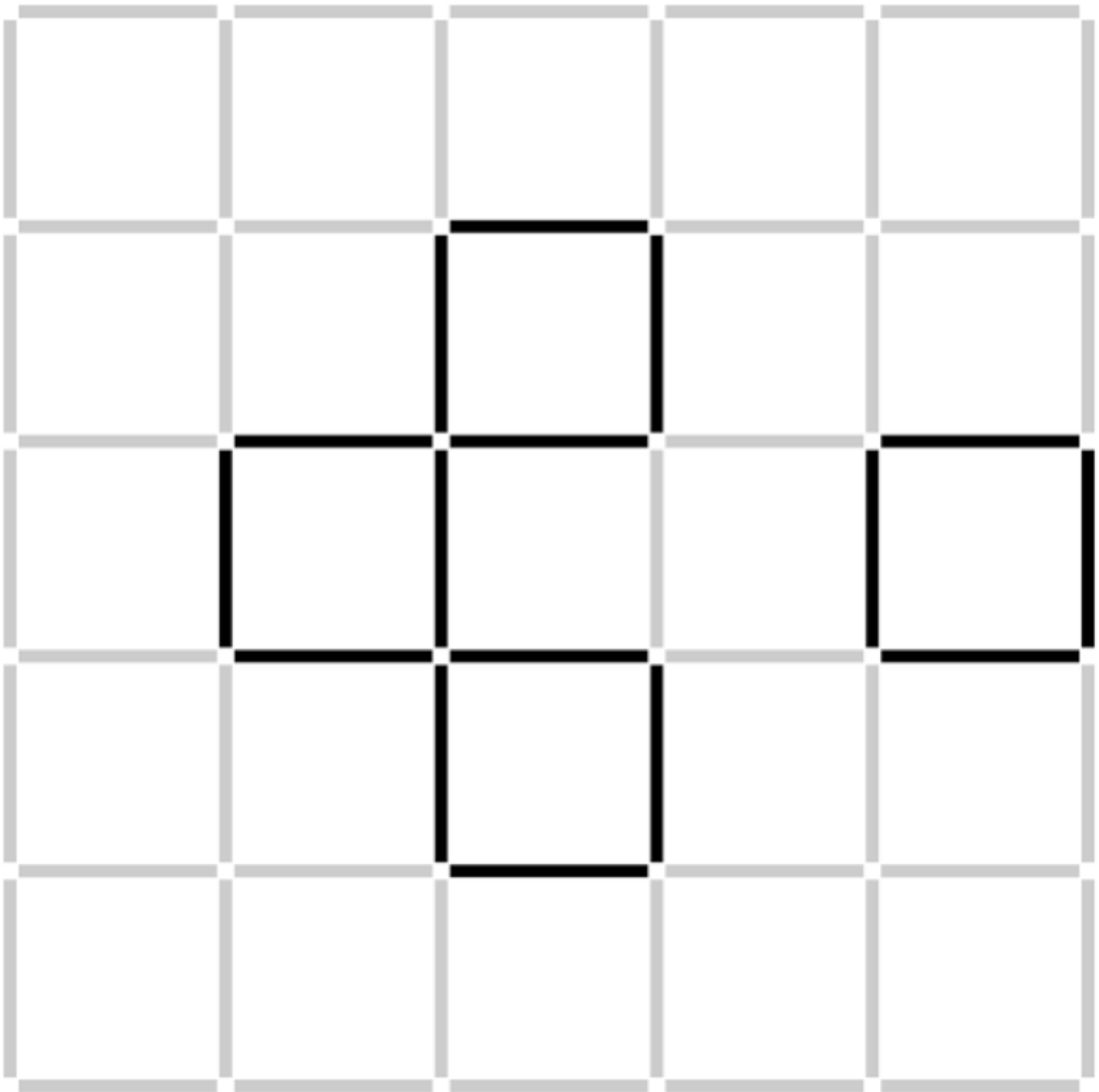

Supplement: Supplementary file 4 [file DataSheet2.PDF]
