# Supplementary figures and images for: Problem solving stages in the five square problem (part 1 of 2)
Source: Front Psychol. 2015 Aug 4;6:1050. doi: 10.3389/fpsyg.2015.01050 (PMC4523725; doi:10.3389/fpsyg.2015.01050)

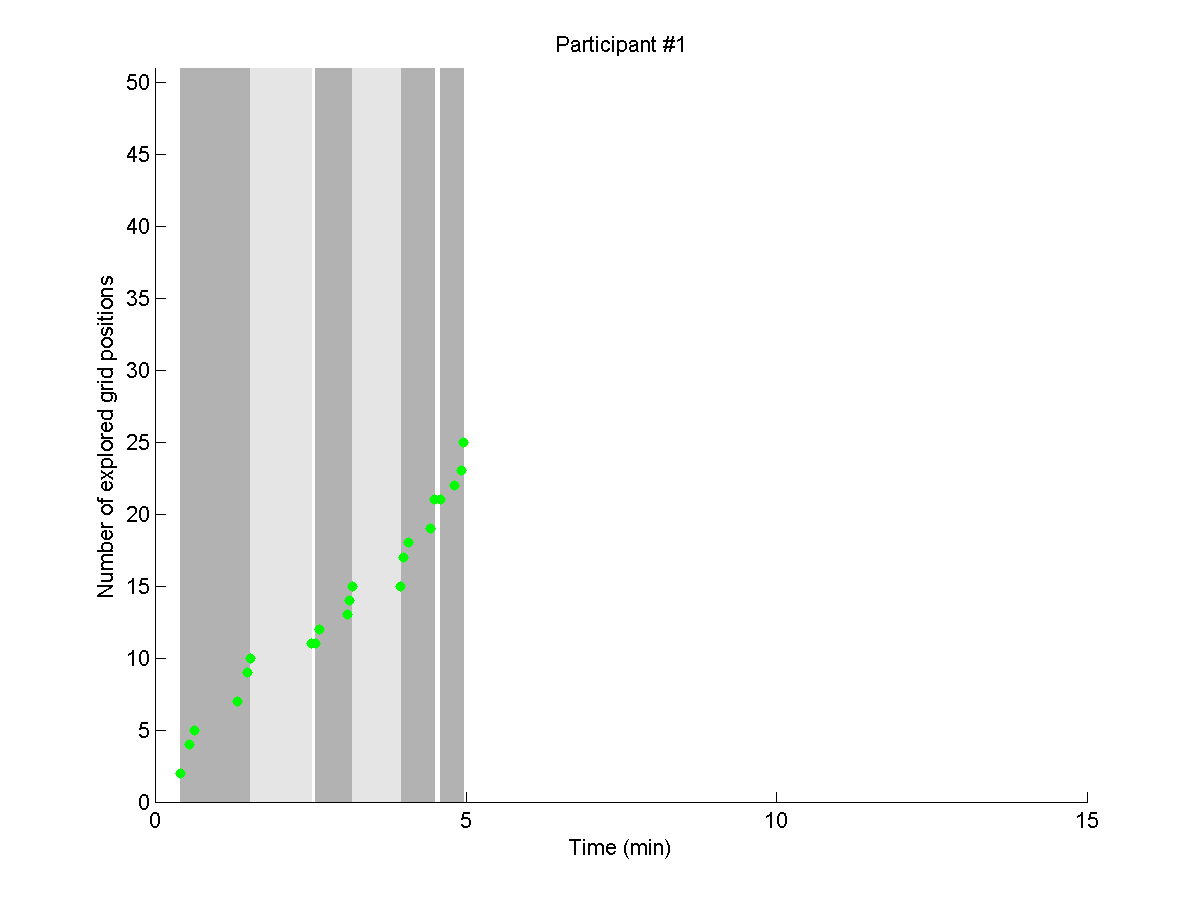

Supplement: Supplementary file 1 [file Presentation1.ZIP › individual plots/1.png]

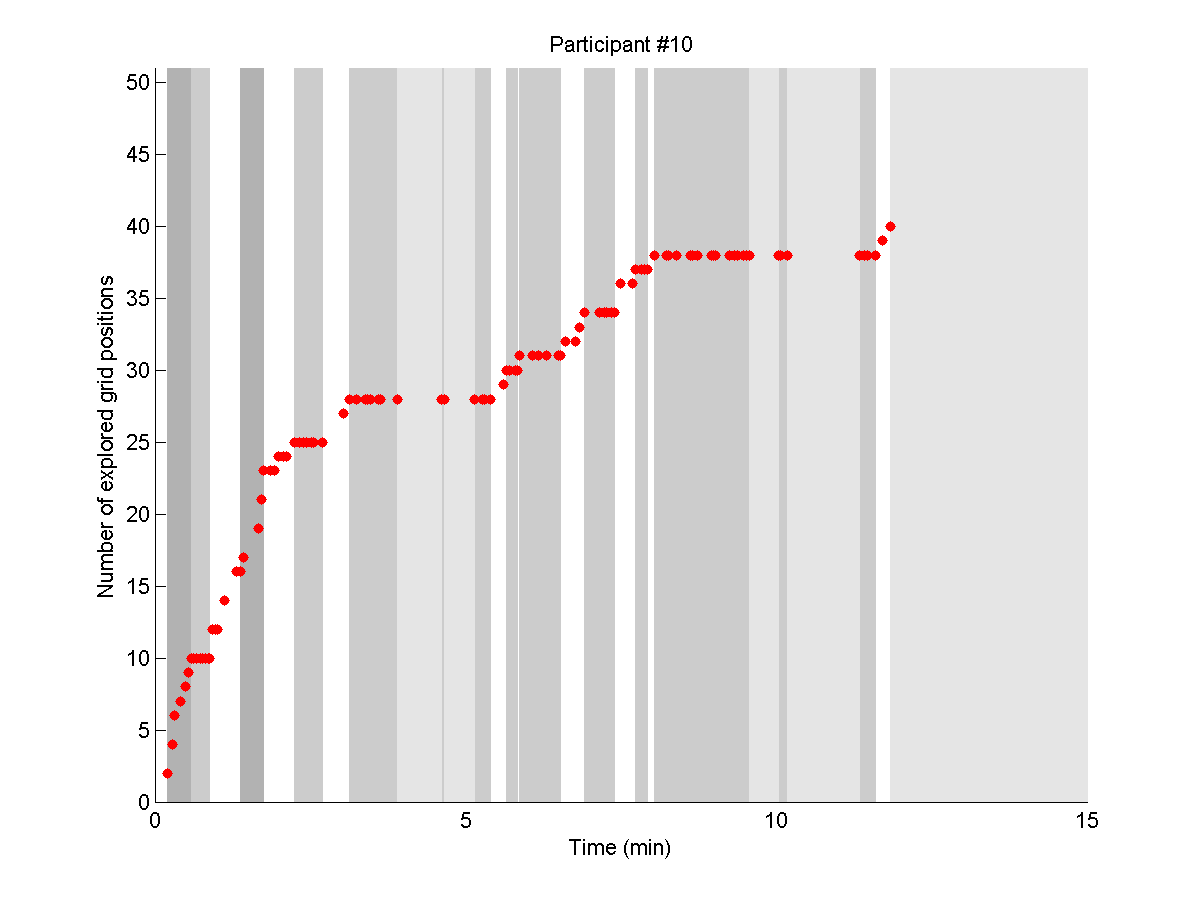

Supplement: Supplementary file 1 [file Presentation1.ZIP › individual plots/10.png]

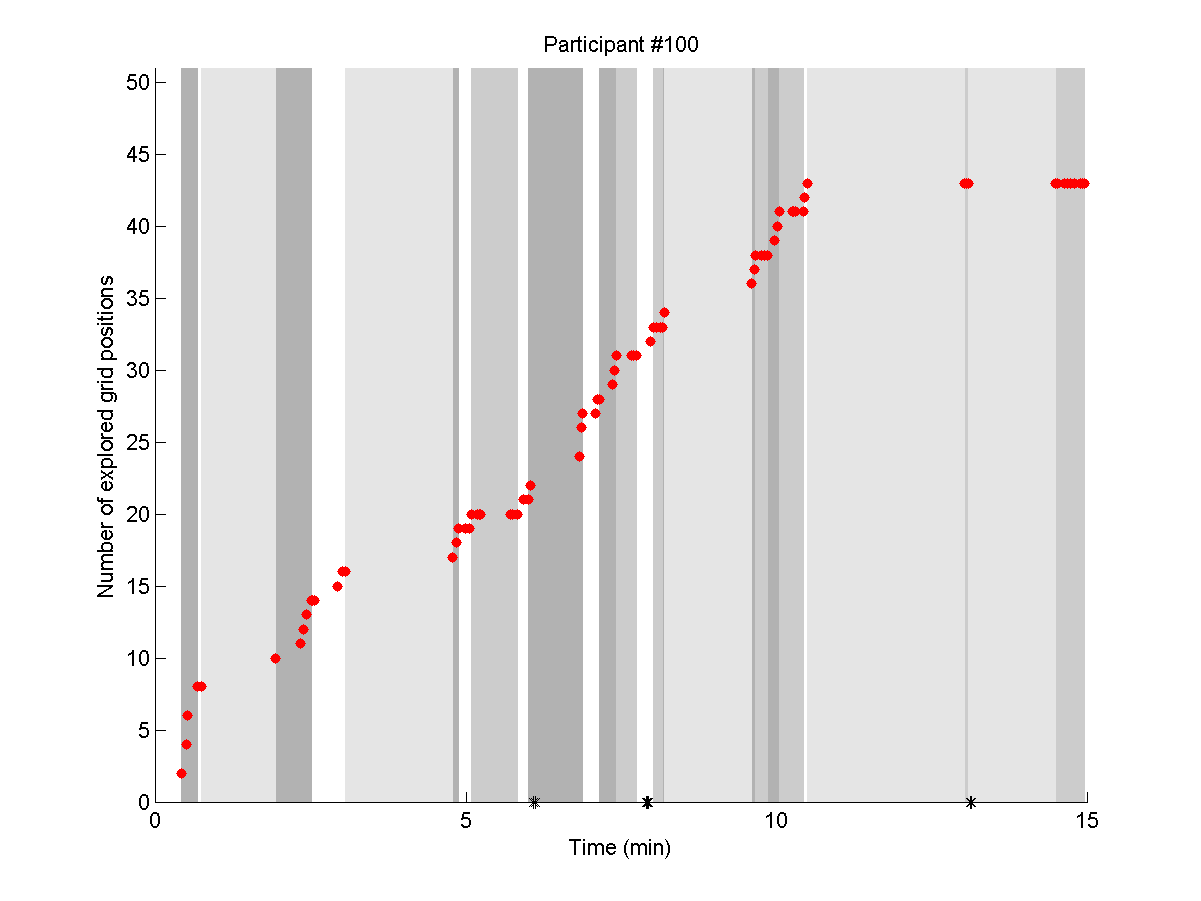

Supplement: Supplementary file 1 [file Presentation1.ZIP › individual plots/100.png]

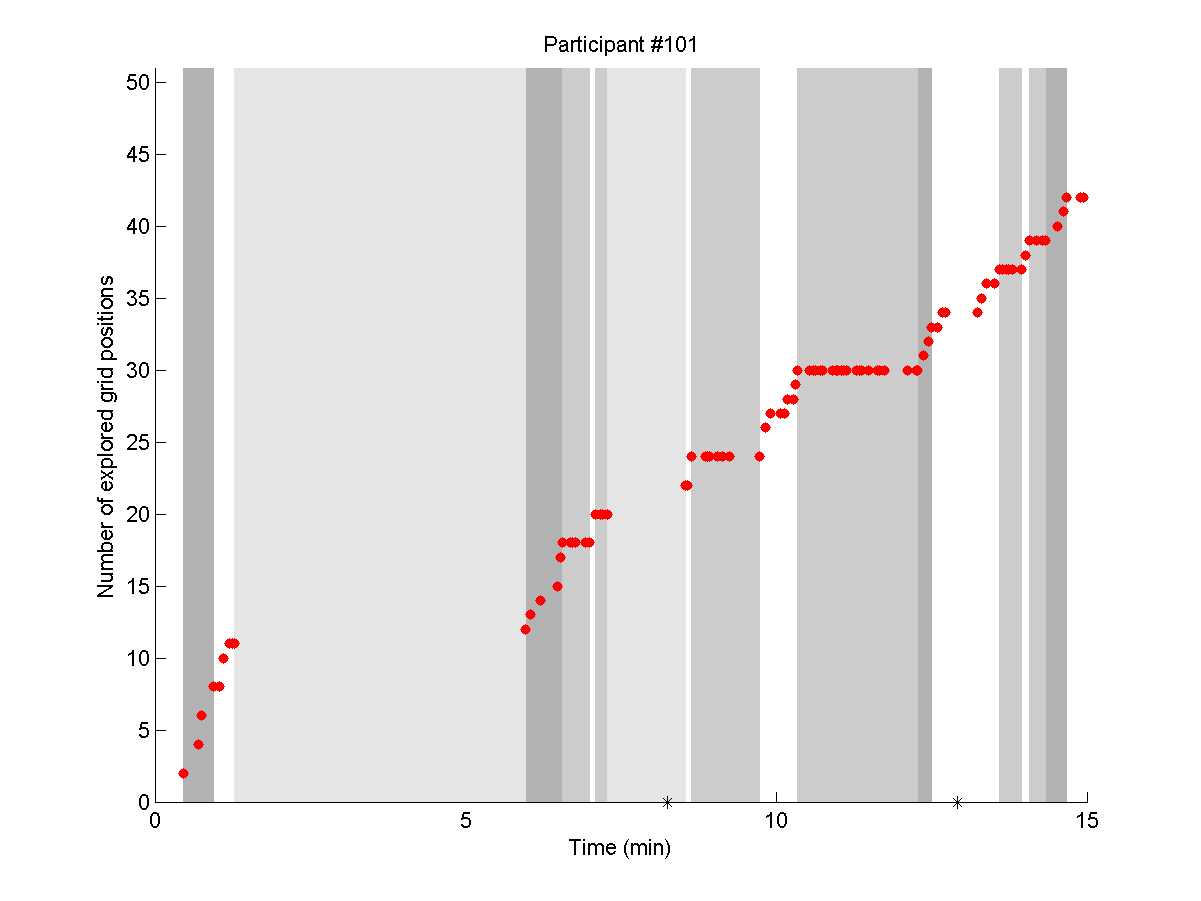

Supplement: Supplementary file 1 [file Presentation1.ZIP › individual plots/101.png]

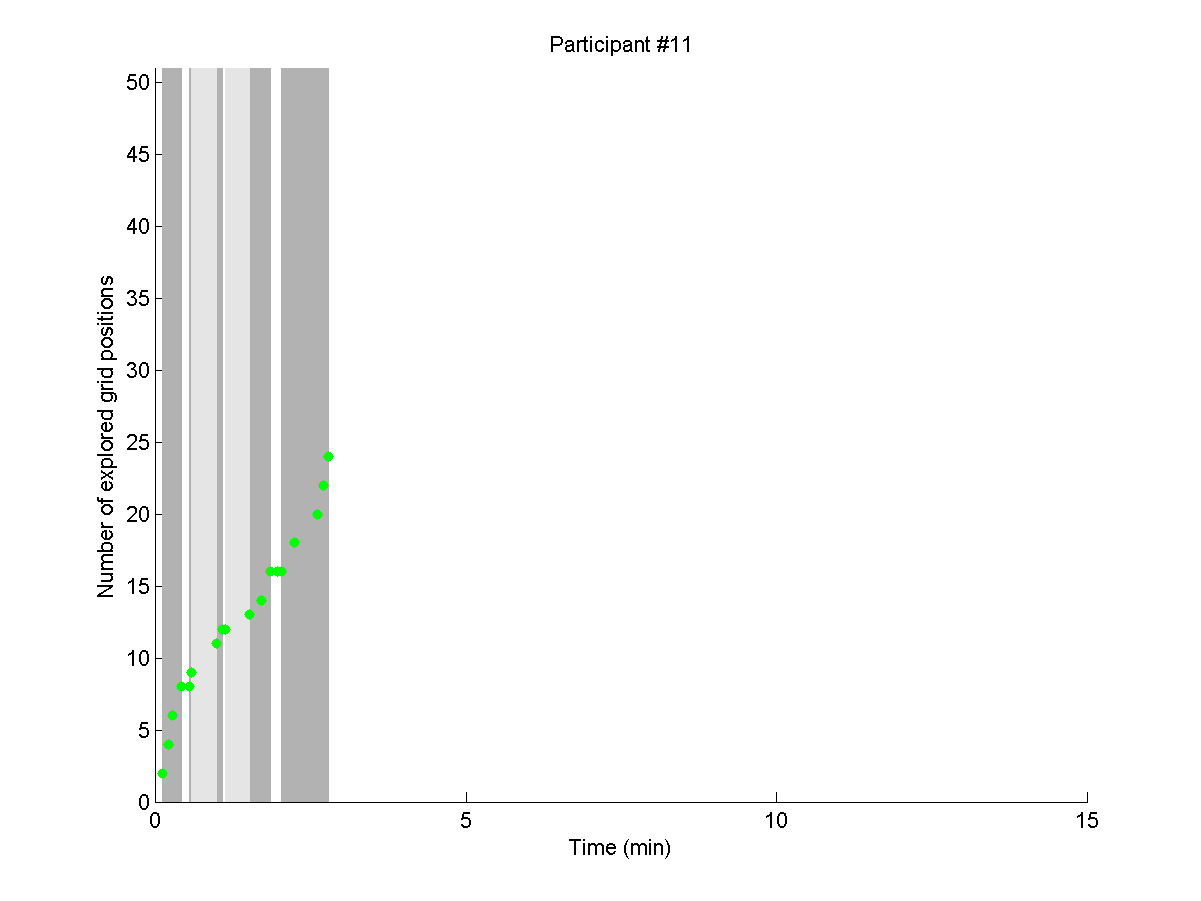

Supplement: Supplementary file 1 [file Presentation1.ZIP › individual plots/11.png]

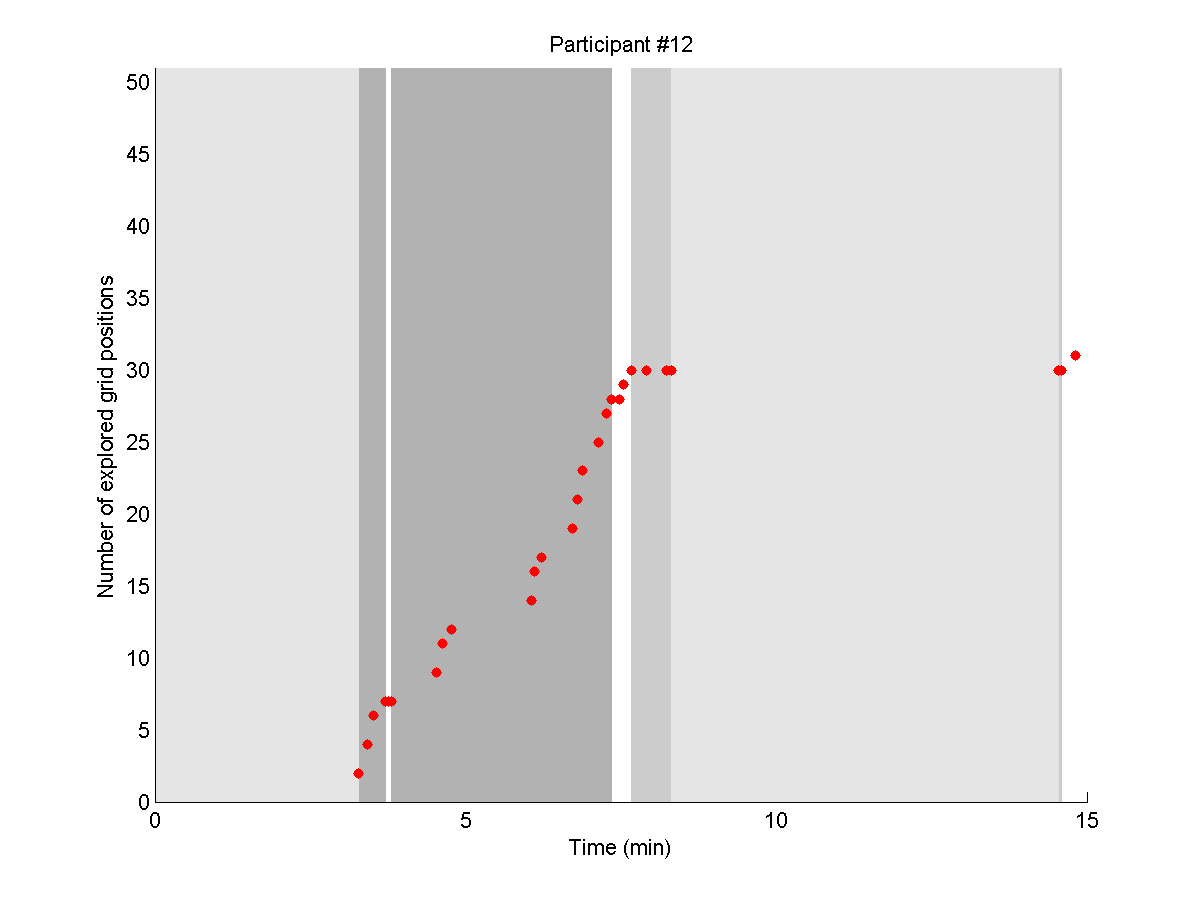

Supplement: Supplementary file 1 [file Presentation1.ZIP › individual plots/12.png]

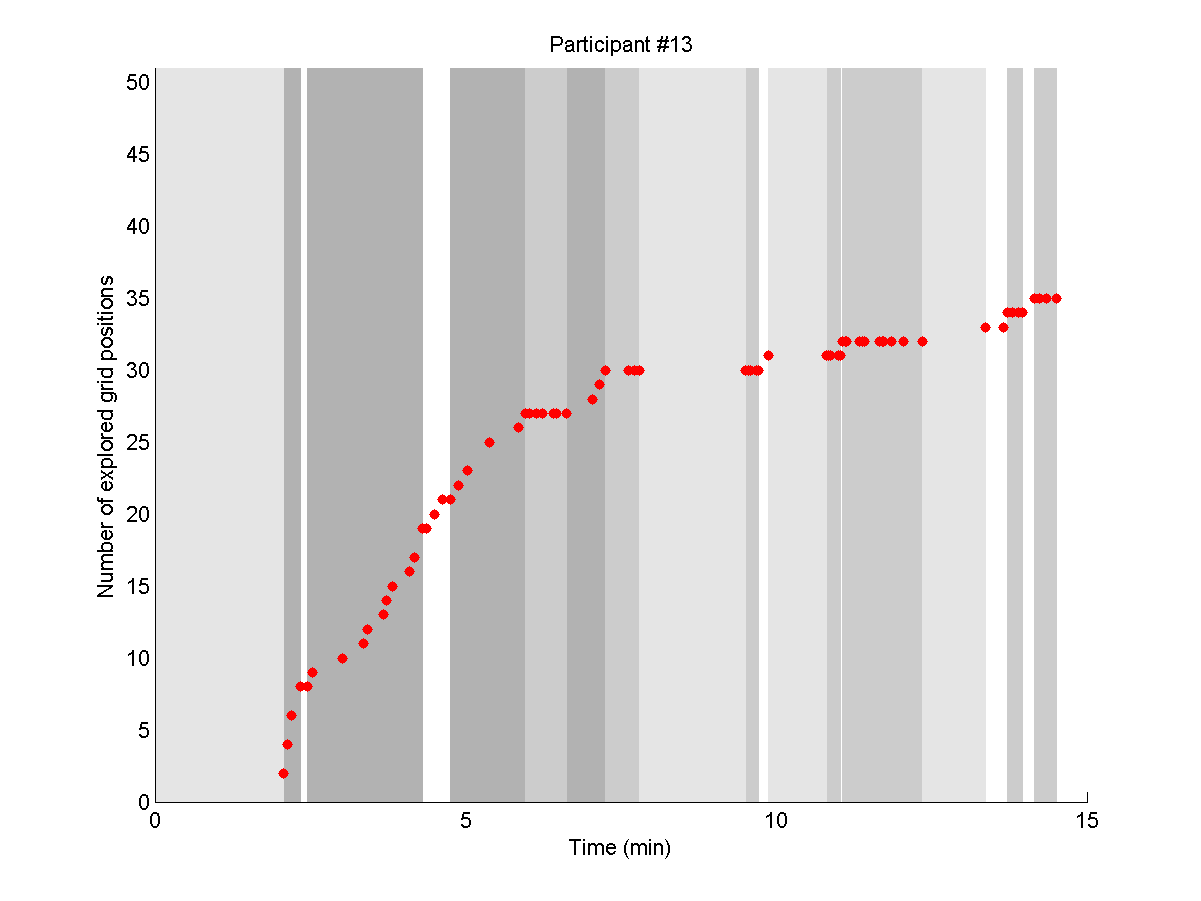

Supplement: Supplementary file 1 [file Presentation1.ZIP › individual plots/13.png]

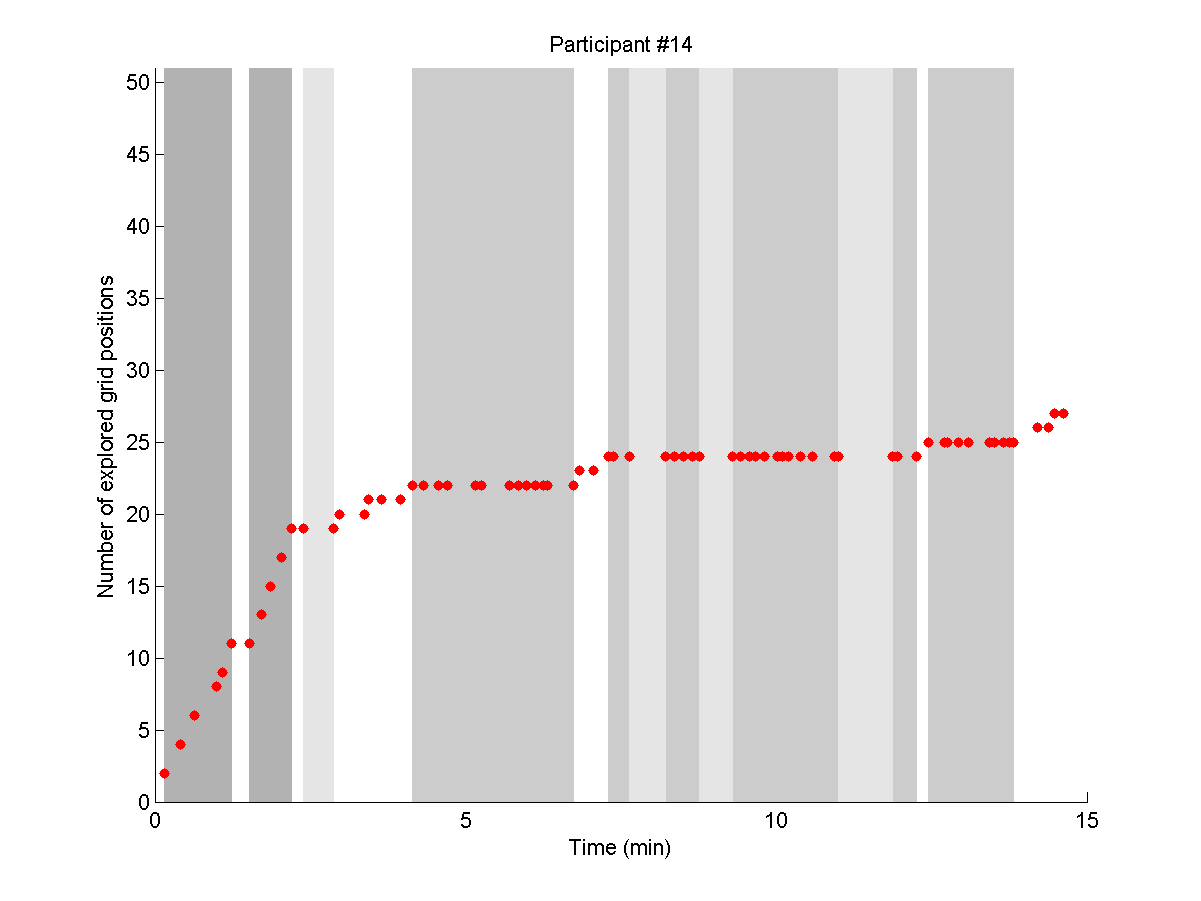

Supplement: Supplementary file 1 [file Presentation1.ZIP › individual plots/14.png]

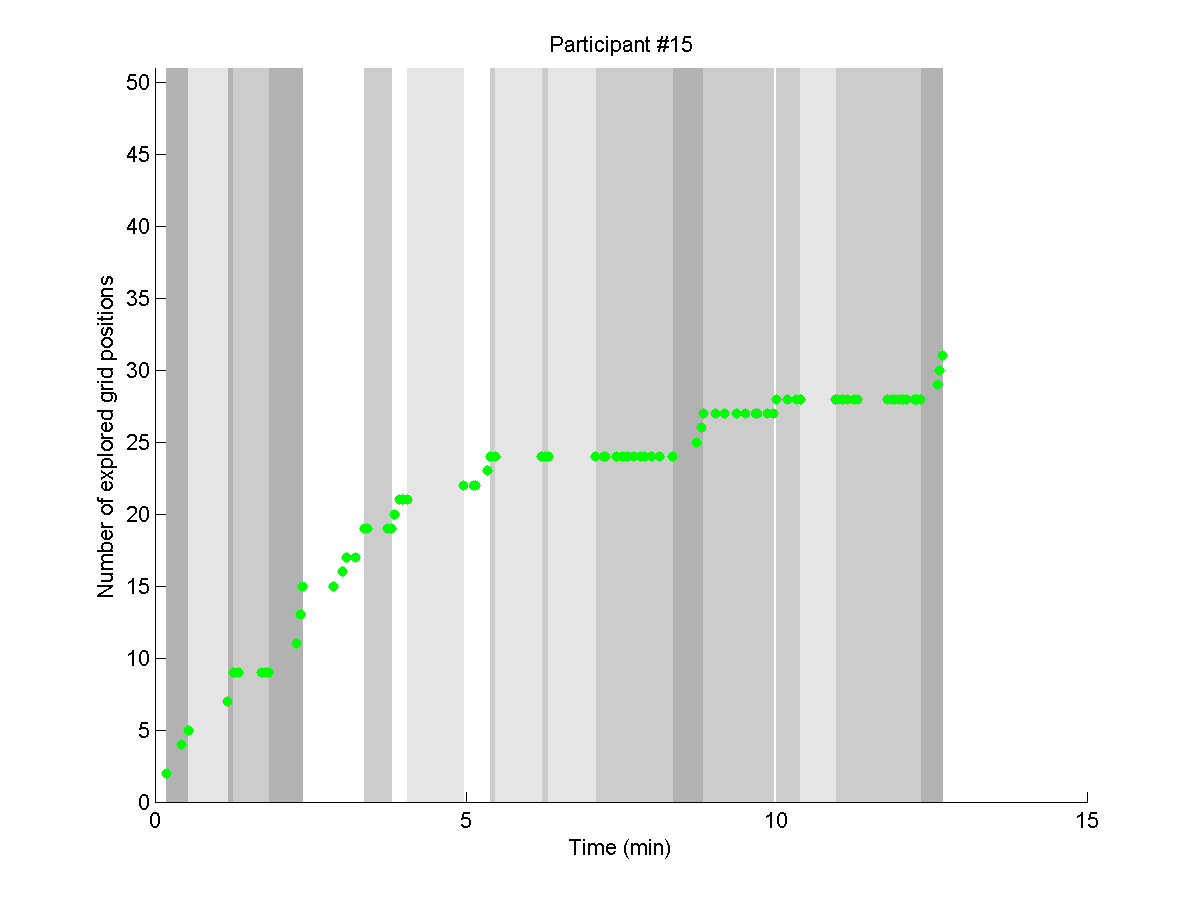

Supplement: Supplementary file 1 [file Presentation1.ZIP › individual plots/15.png]

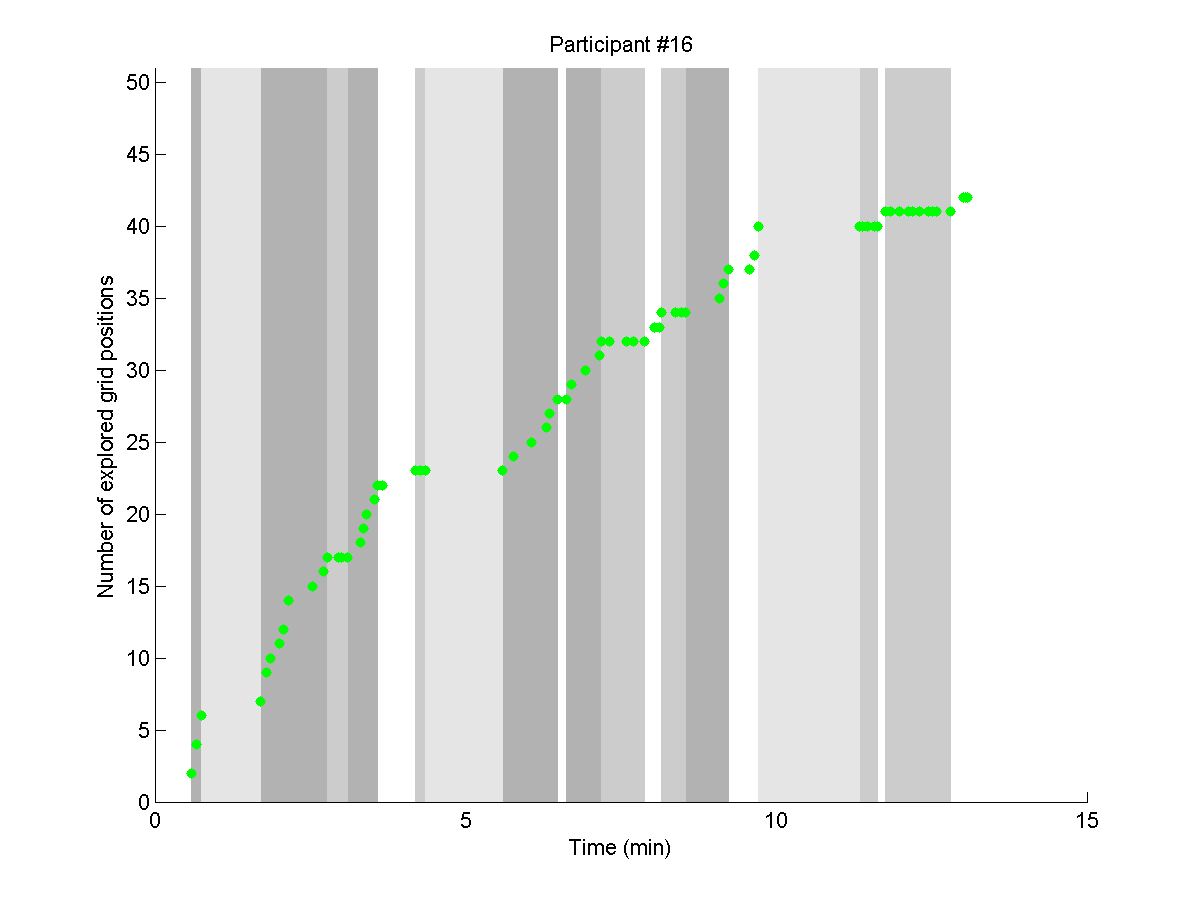

Supplement: Supplementary file 1 [file Presentation1.ZIP › individual plots/16.png]

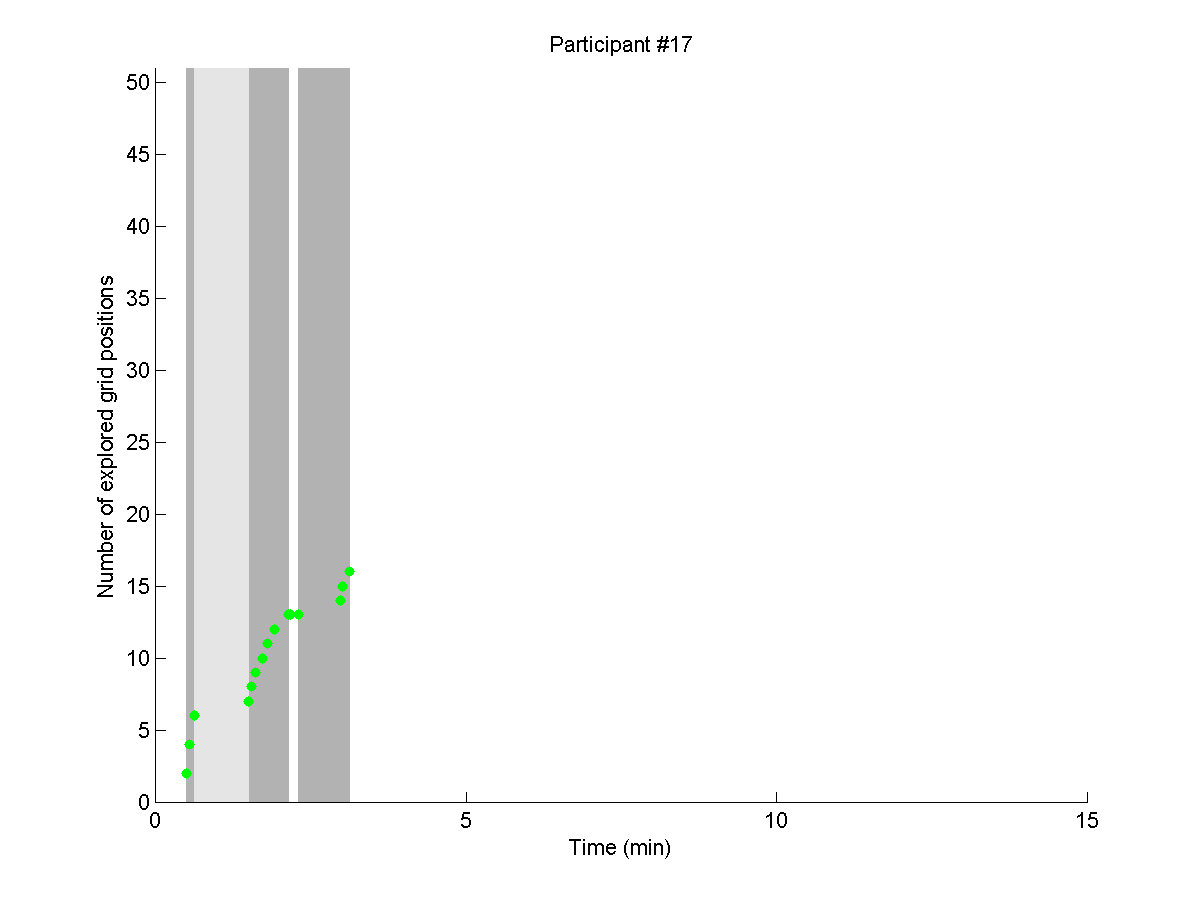

Supplement: Supplementary file 1 [file Presentation1.ZIP › individual plots/17.png]

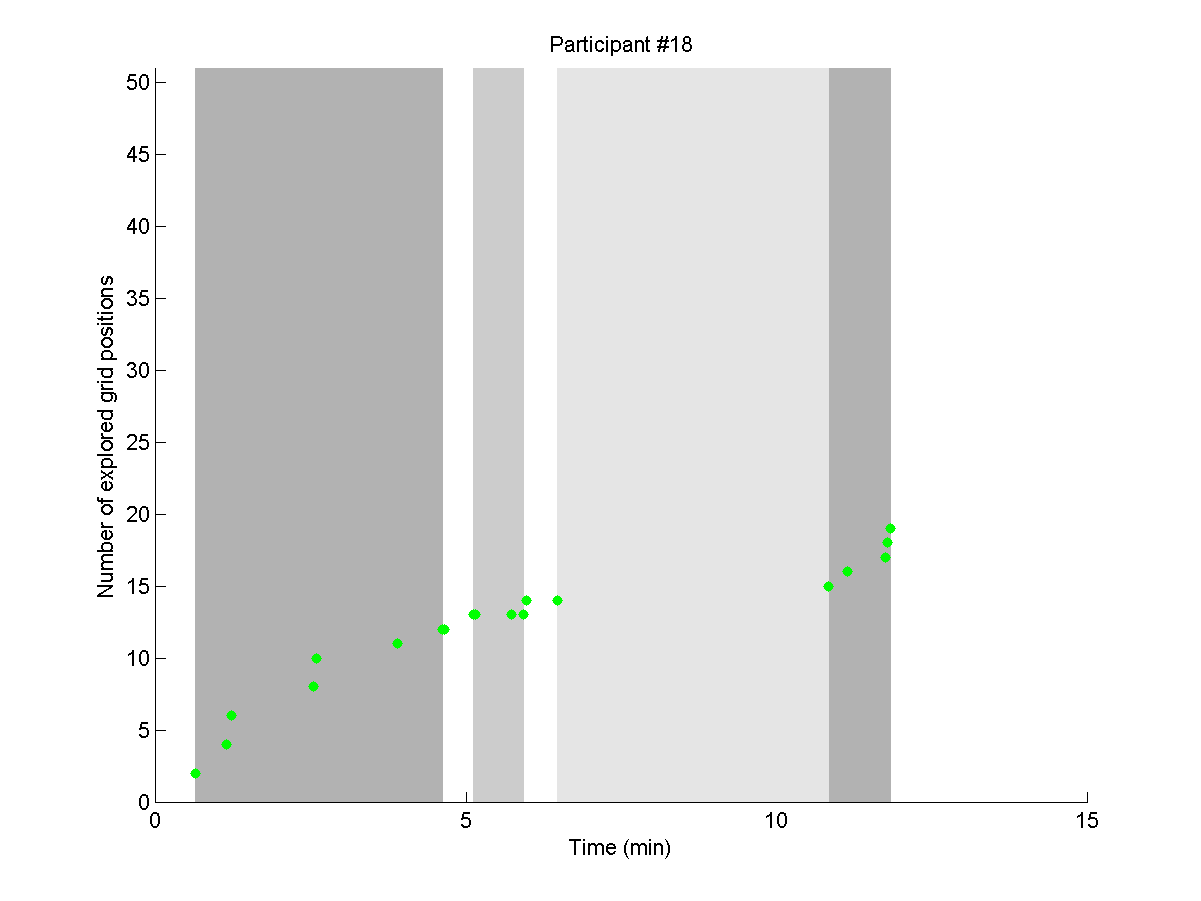

Supplement: Supplementary file 1 [file Presentation1.ZIP › individual plots/18.png]

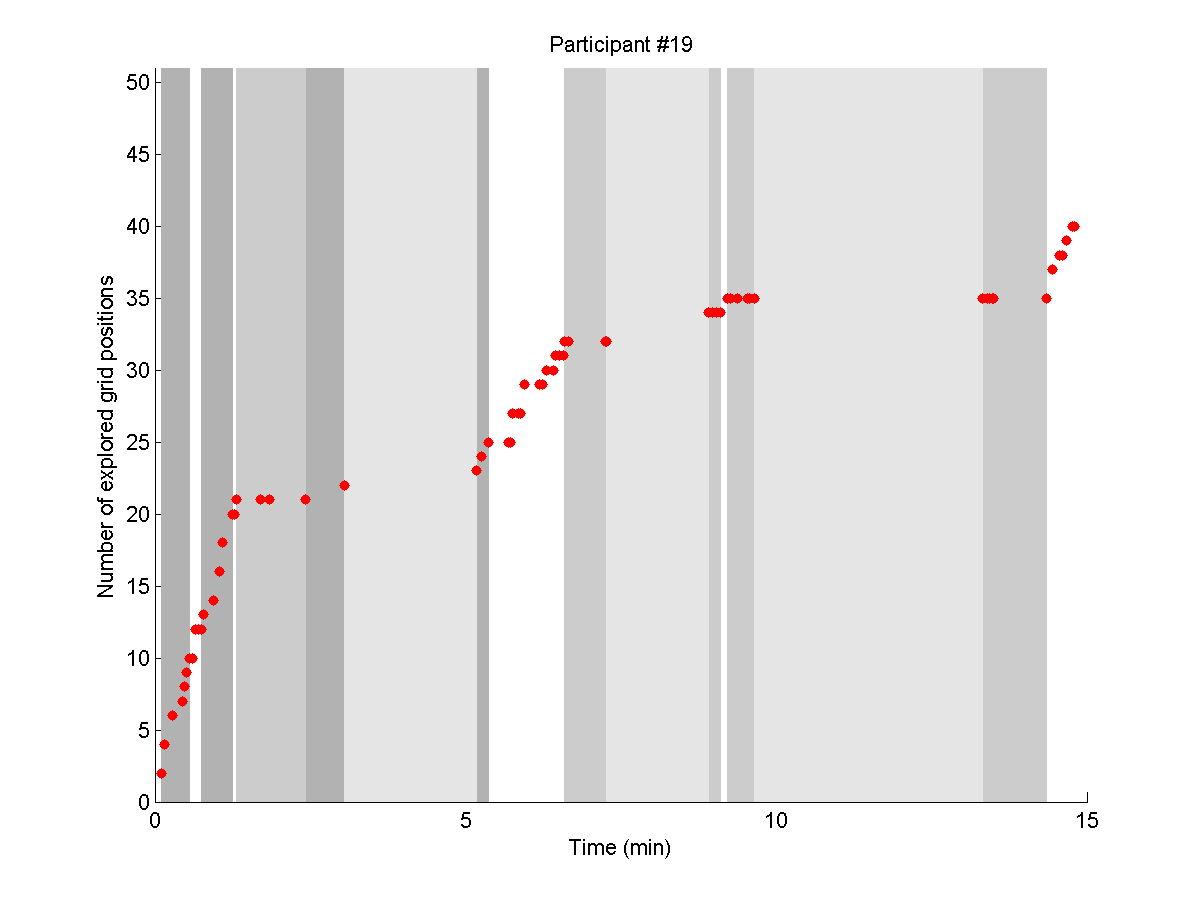

Supplement: Supplementary file 1 [file Presentation1.ZIP › individual plots/19.png]

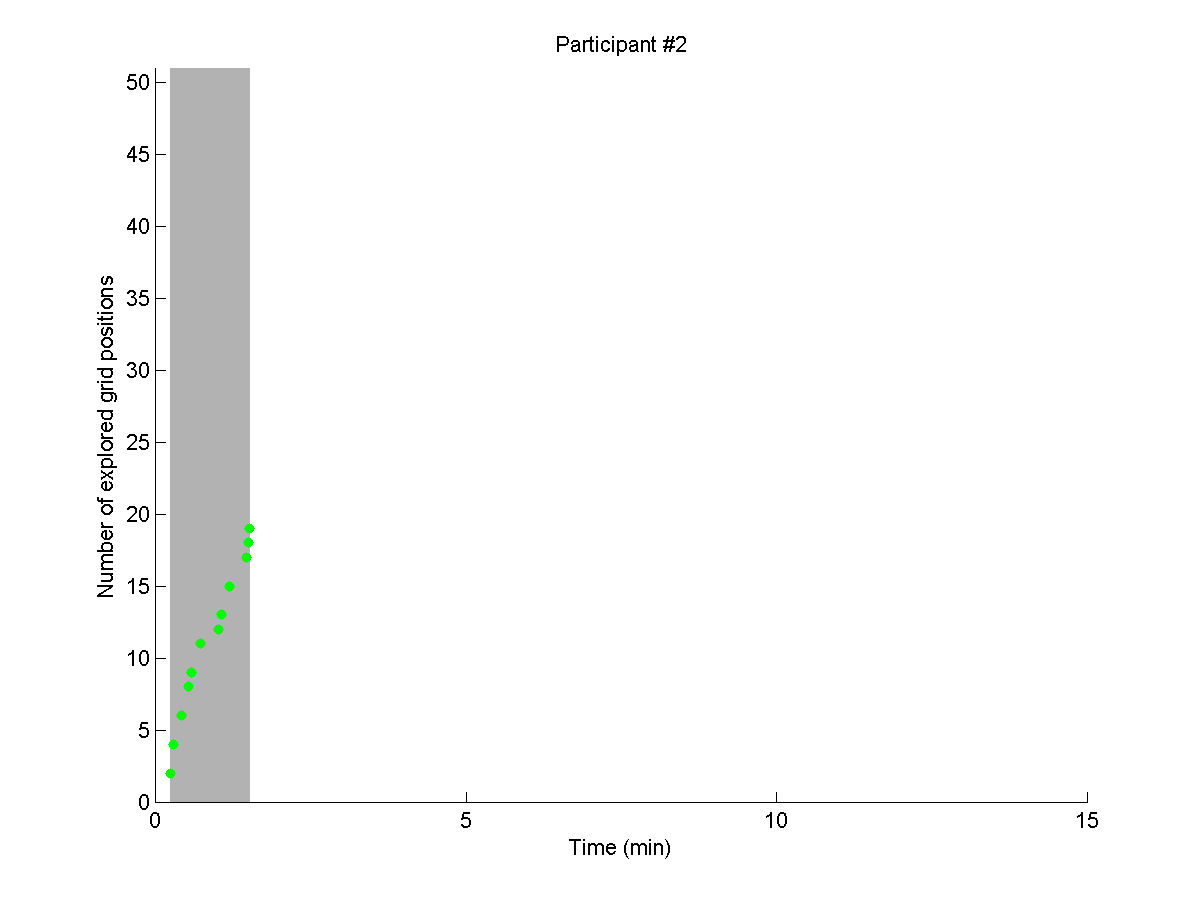

Supplement: Supplementary file 1 [file Presentation1.ZIP › individual plots/2.png]

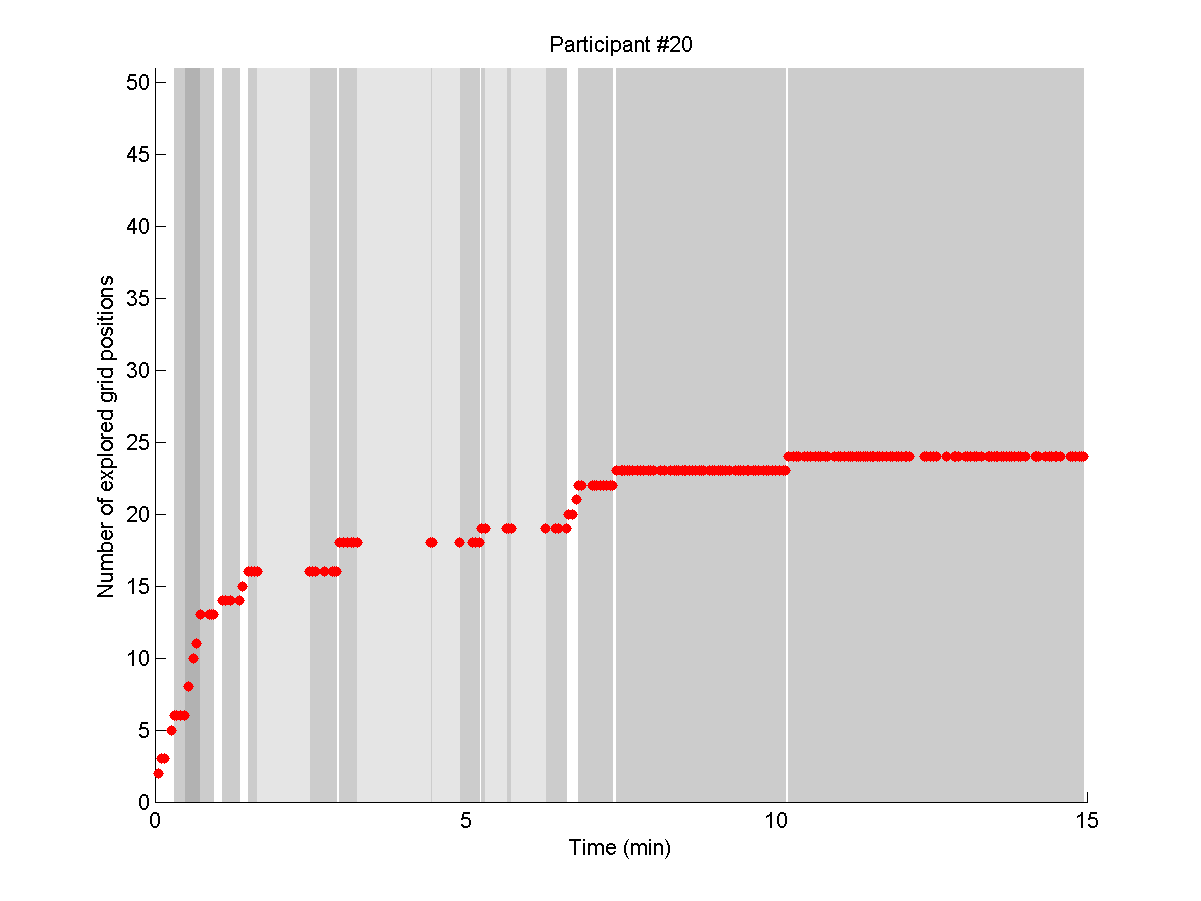

Supplement: Supplementary file 1 [file Presentation1.ZIP › individual plots/20.png]

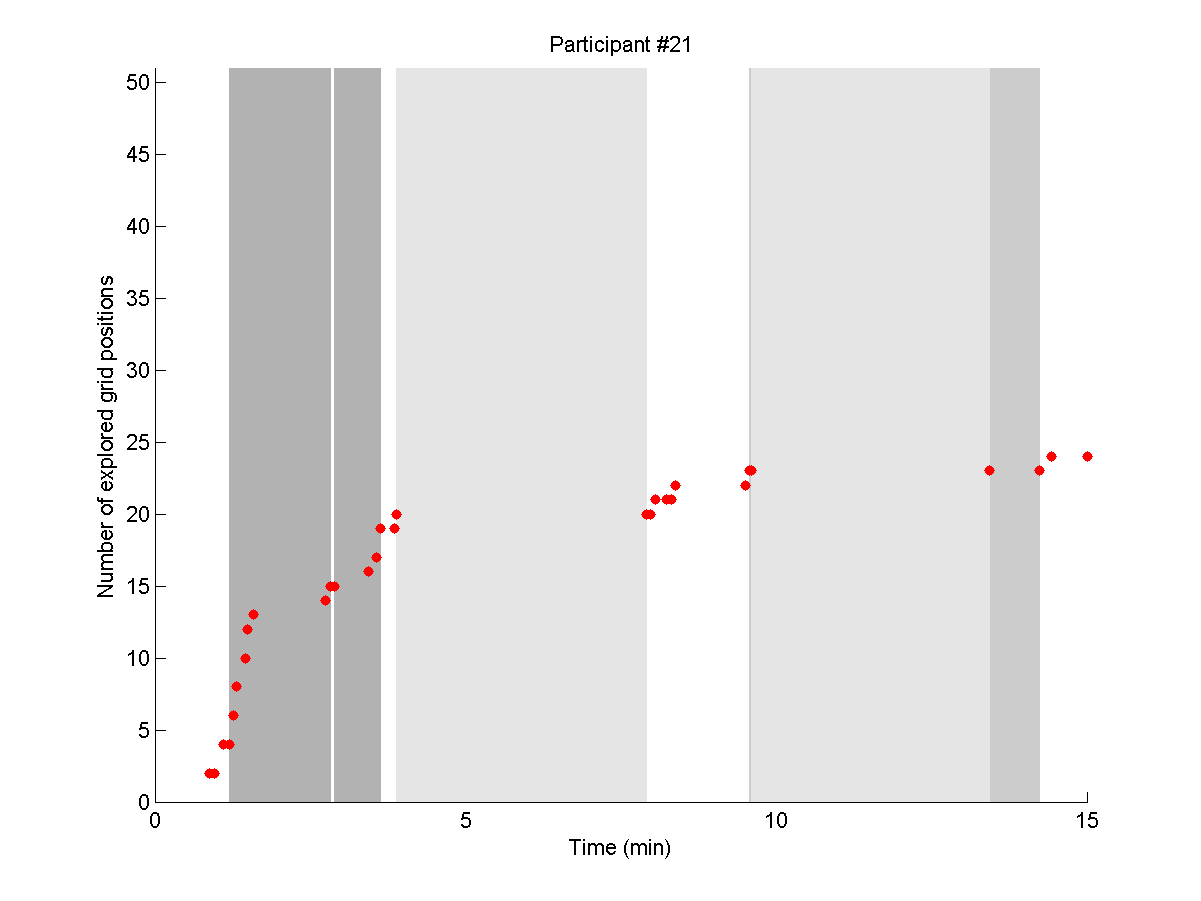

Supplement: Supplementary file 1 [file Presentation1.ZIP › individual plots/21.png]

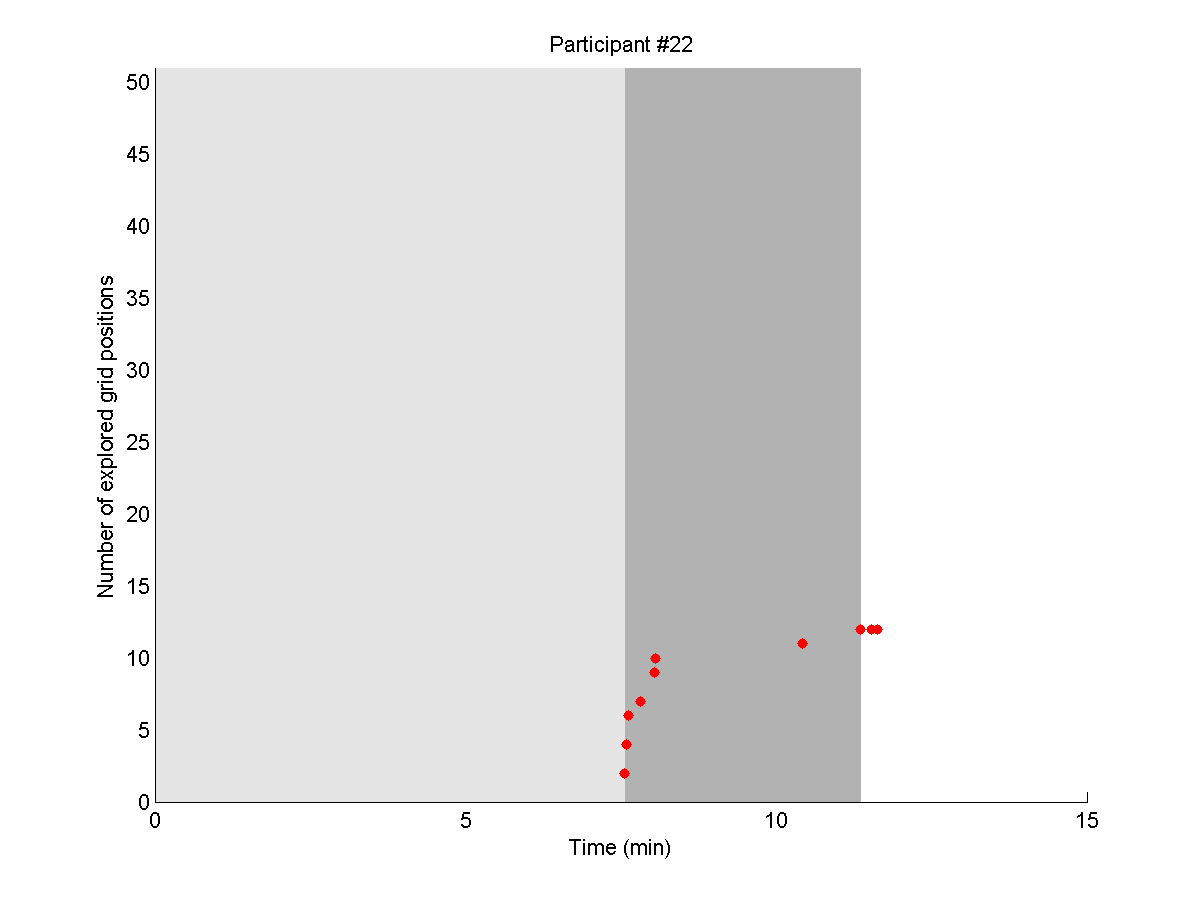

Supplement: Supplementary file 1 [file Presentation1.ZIP › individual plots/22.png]

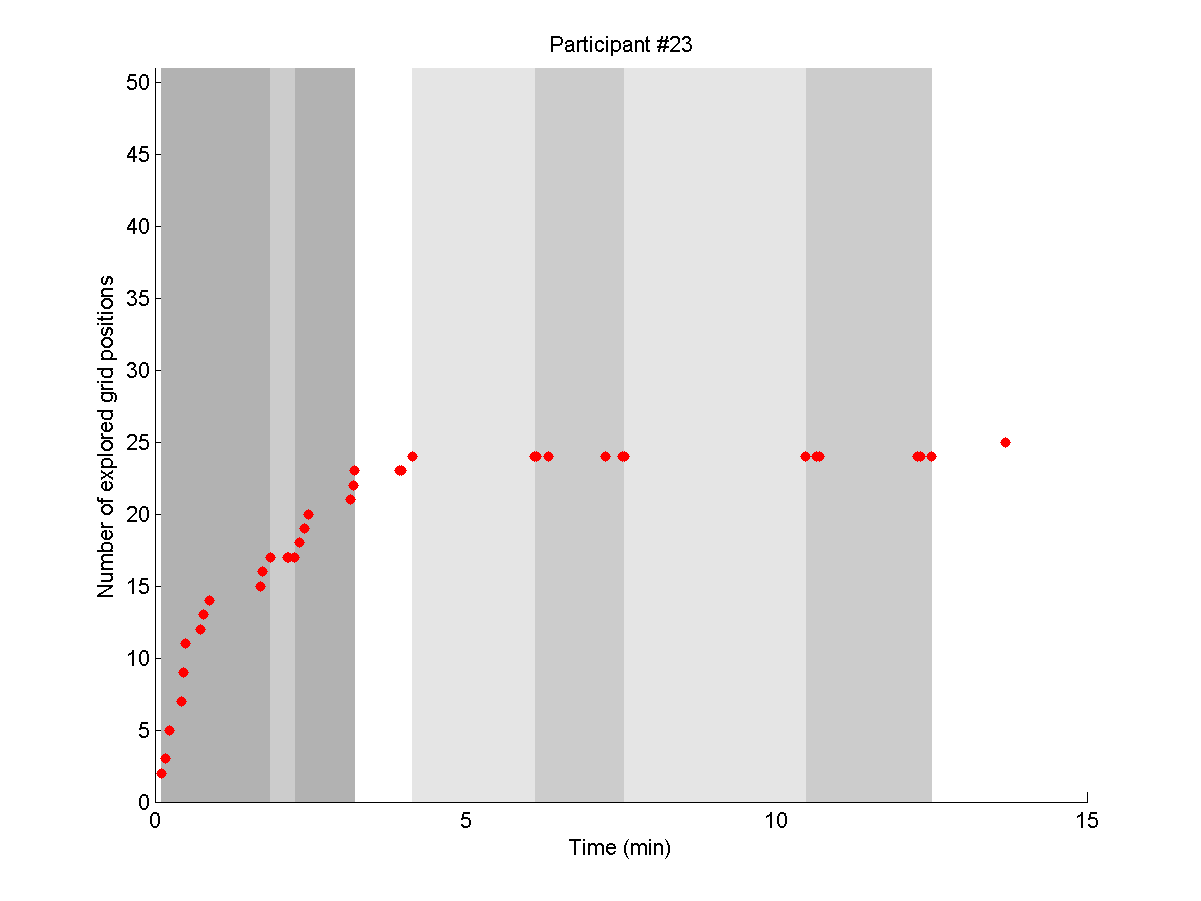

Supplement: Supplementary file 1 [file Presentation1.ZIP › individual plots/23.png]

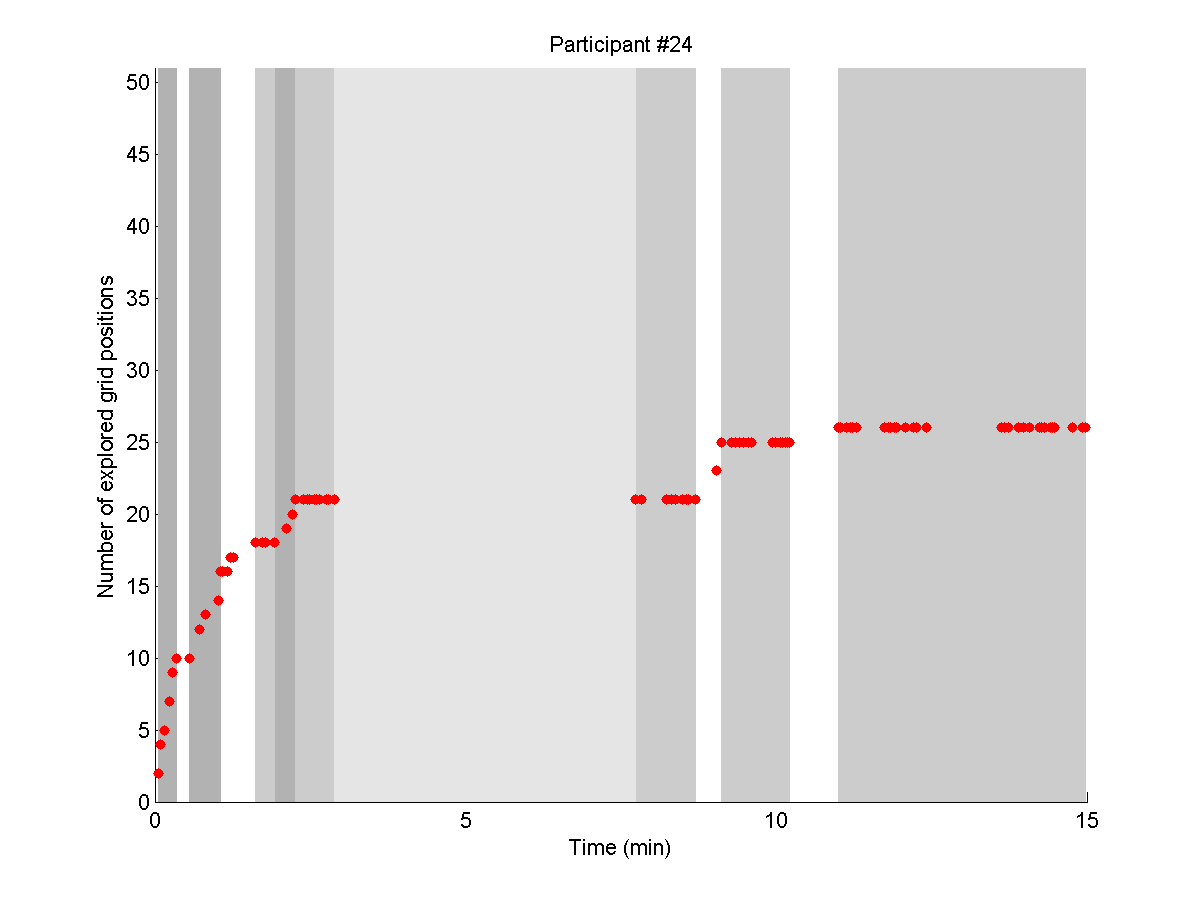

Supplement: Supplementary file 1 [file Presentation1.ZIP › individual plots/24.png]

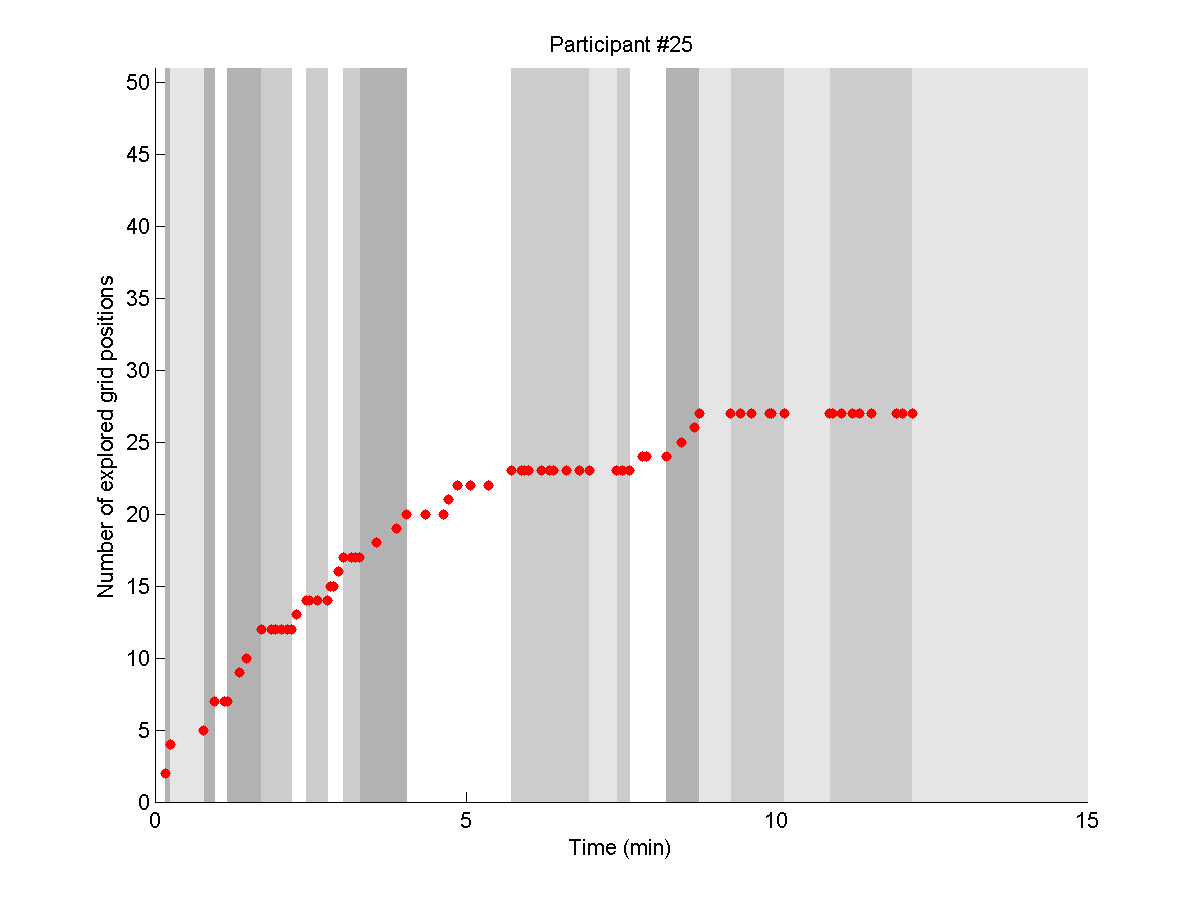

Supplement: Supplementary file 1 [file Presentation1.ZIP › individual plots/25.png]

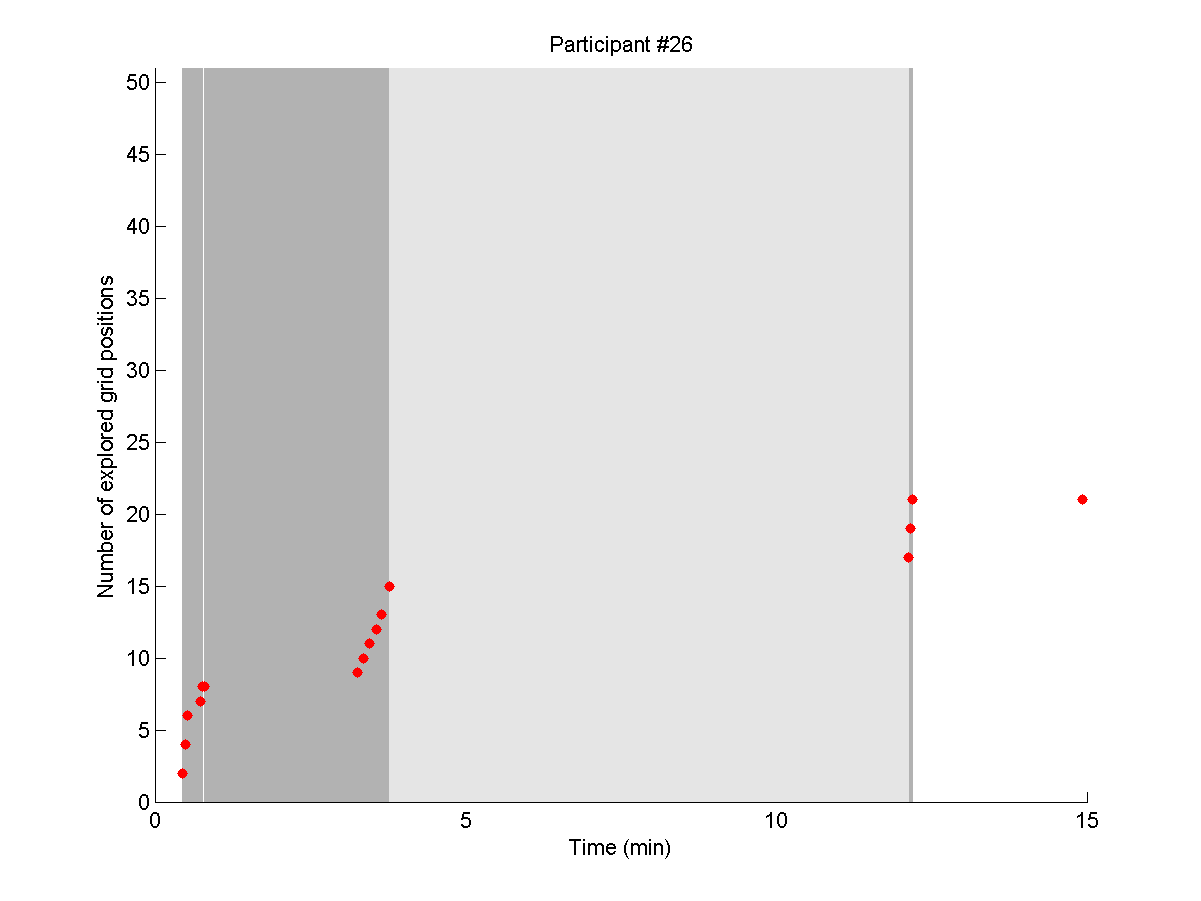

Supplement: Supplementary file 1 [file Presentation1.ZIP › individual plots/26.png]

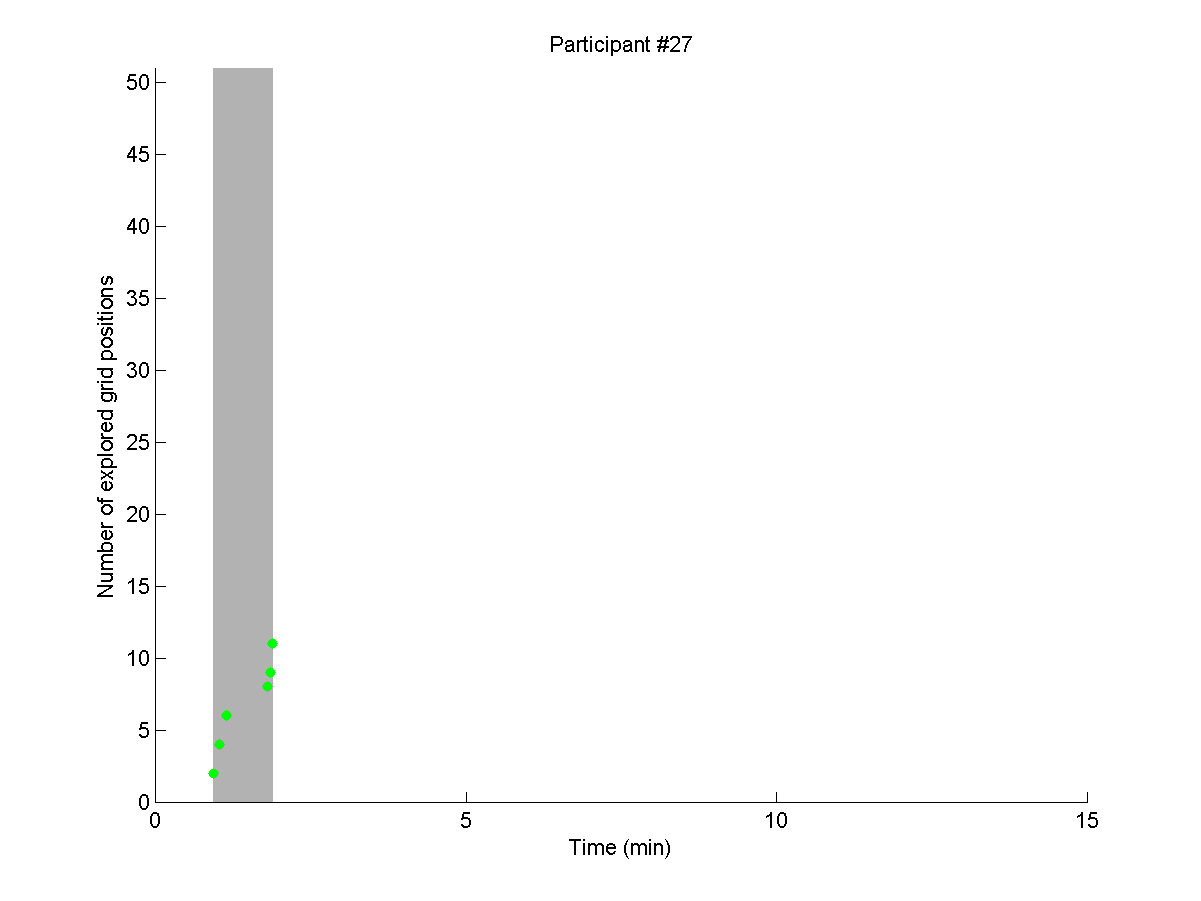

Supplement: Supplementary file 1 [file Presentation1.ZIP › individual plots/27.png]

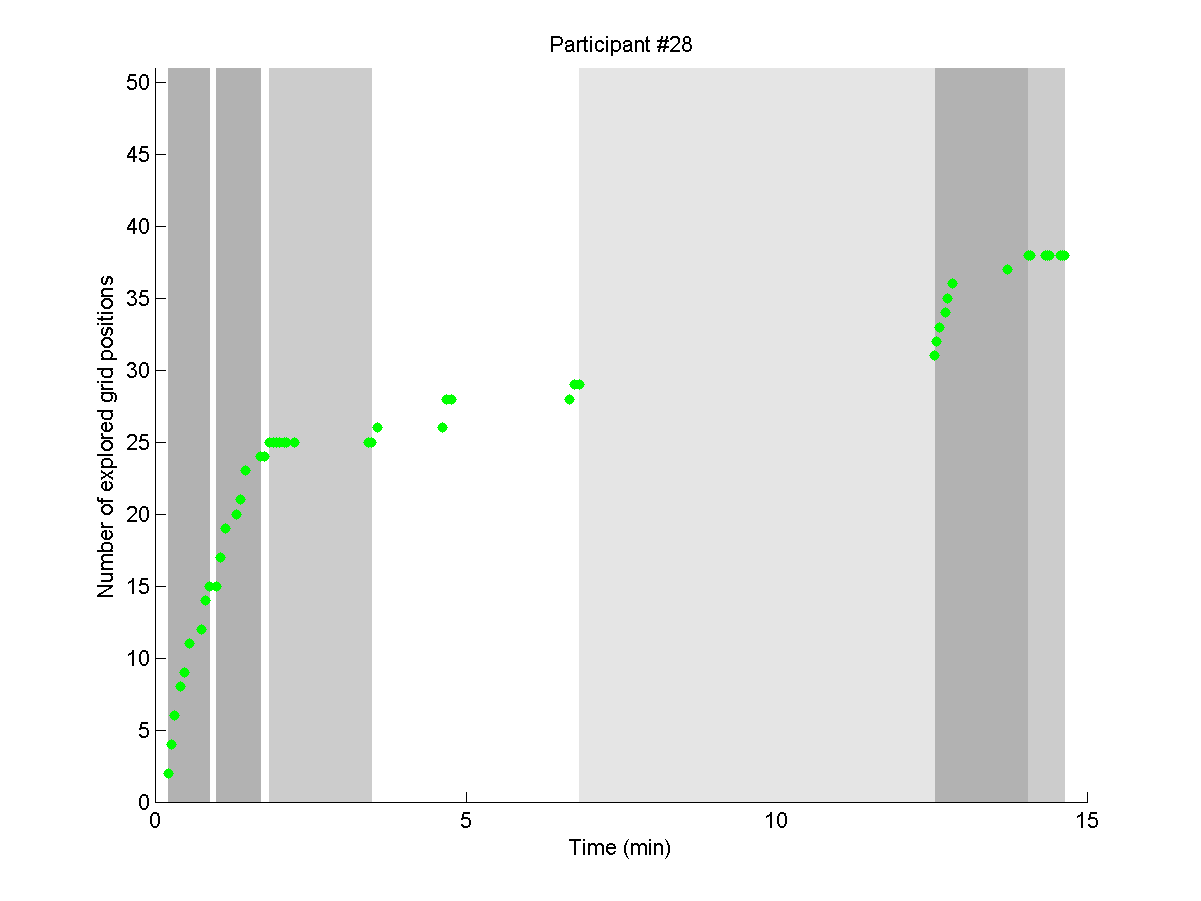

Supplement: Supplementary file 1 [file Presentation1.ZIP › individual plots/28.png]

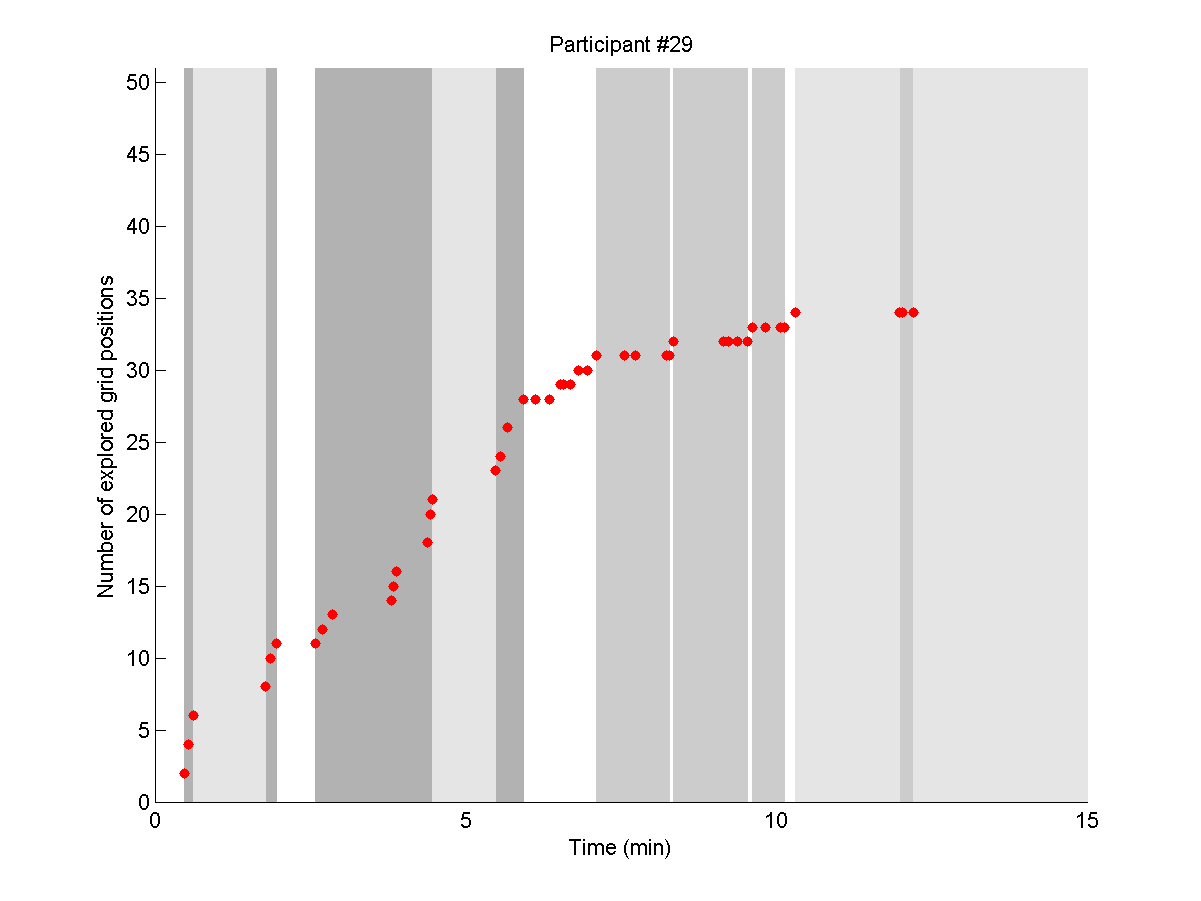

Supplement: Supplementary file 1 [file Presentation1.ZIP › individual plots/29.png]

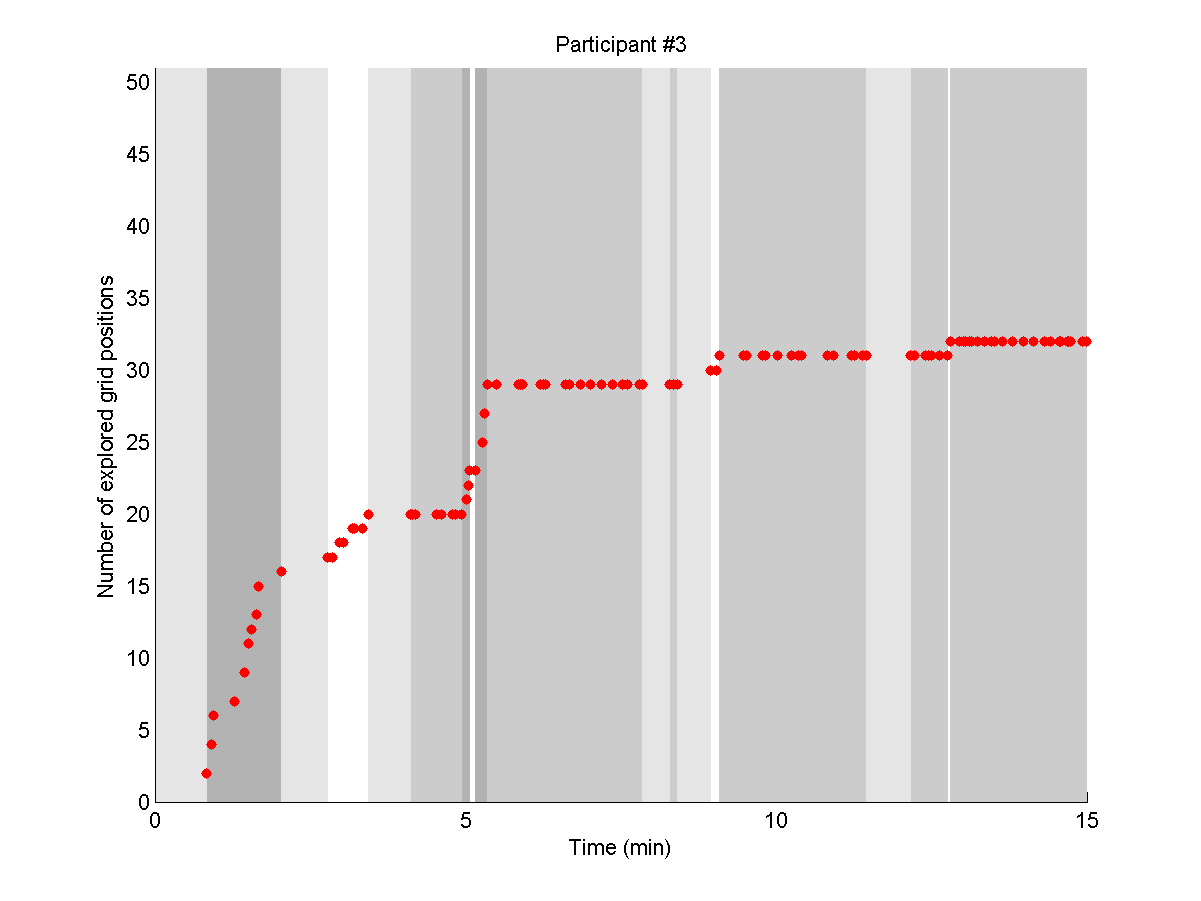

Supplement: Supplementary file 1 [file Presentation1.ZIP › individual plots/3.png]

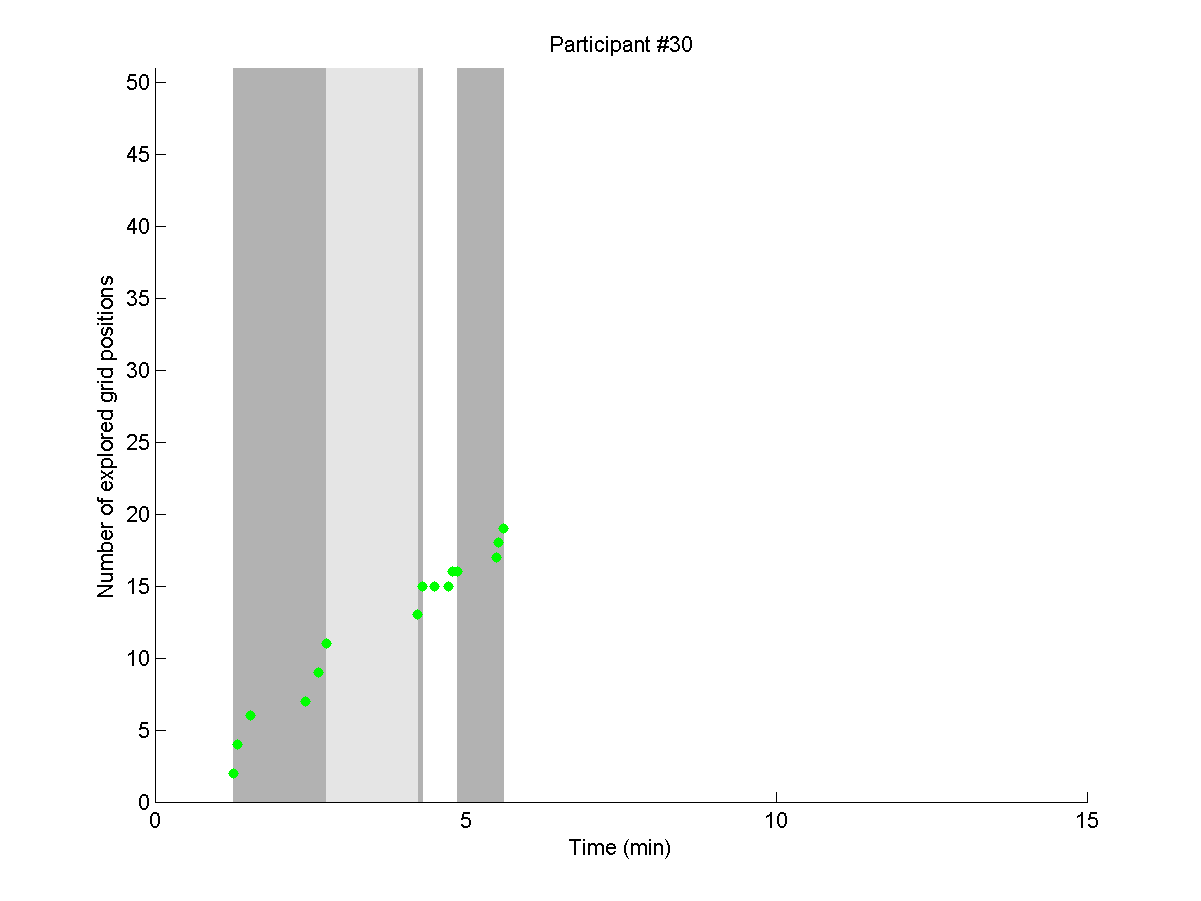

Supplement: Supplementary file 1 [file Presentation1.ZIP › individual plots/30.png]

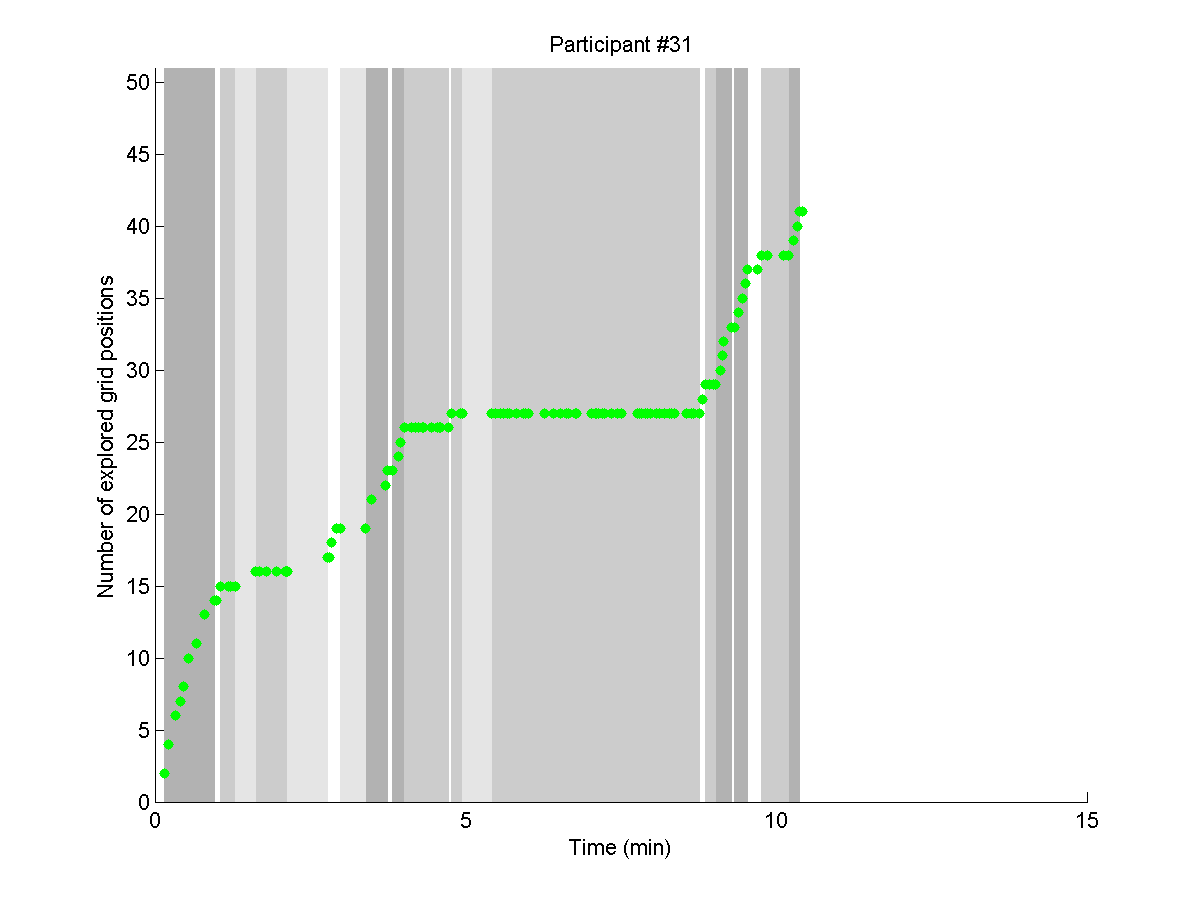

Supplement: Supplementary file 1 [file Presentation1.ZIP › individual plots/31.png]

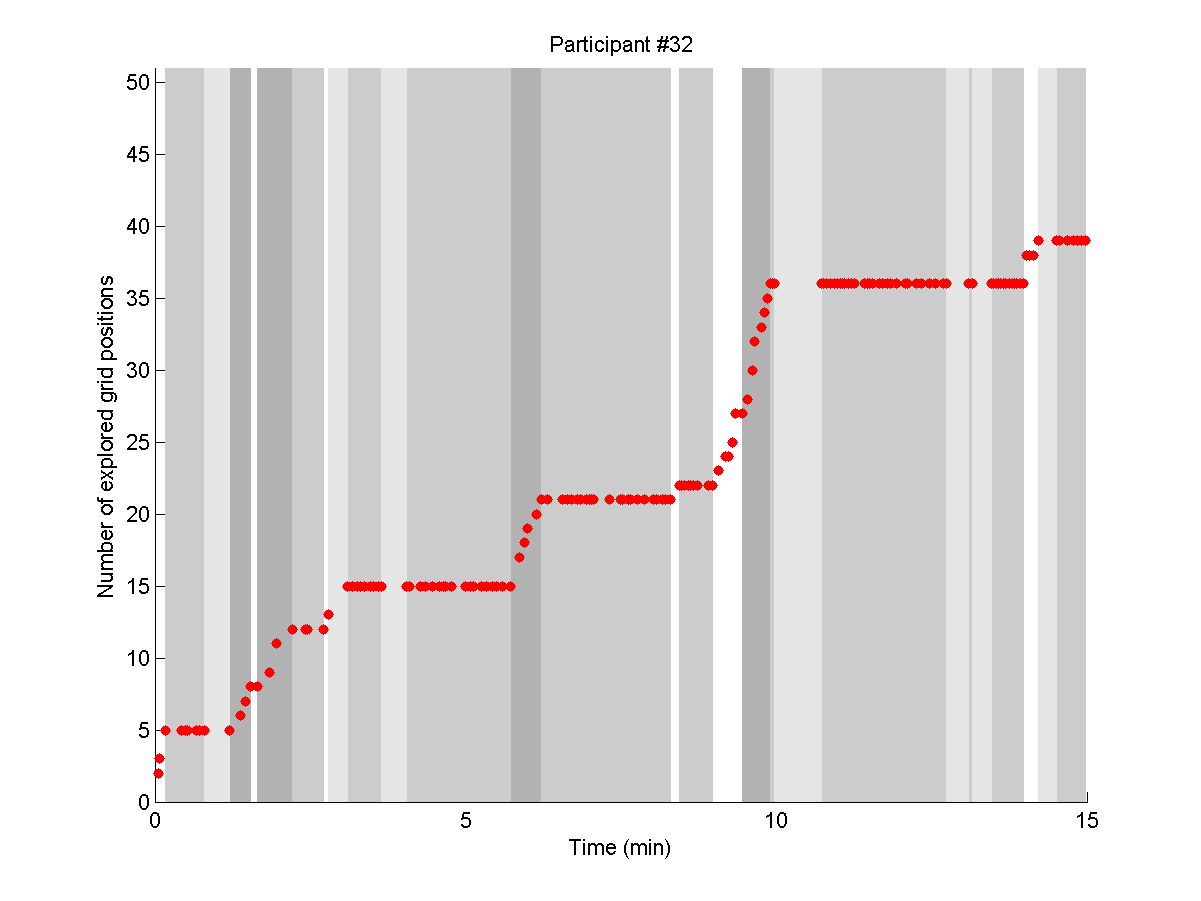

Supplement: Supplementary file 1 [file Presentation1.ZIP › individual plots/32.png]

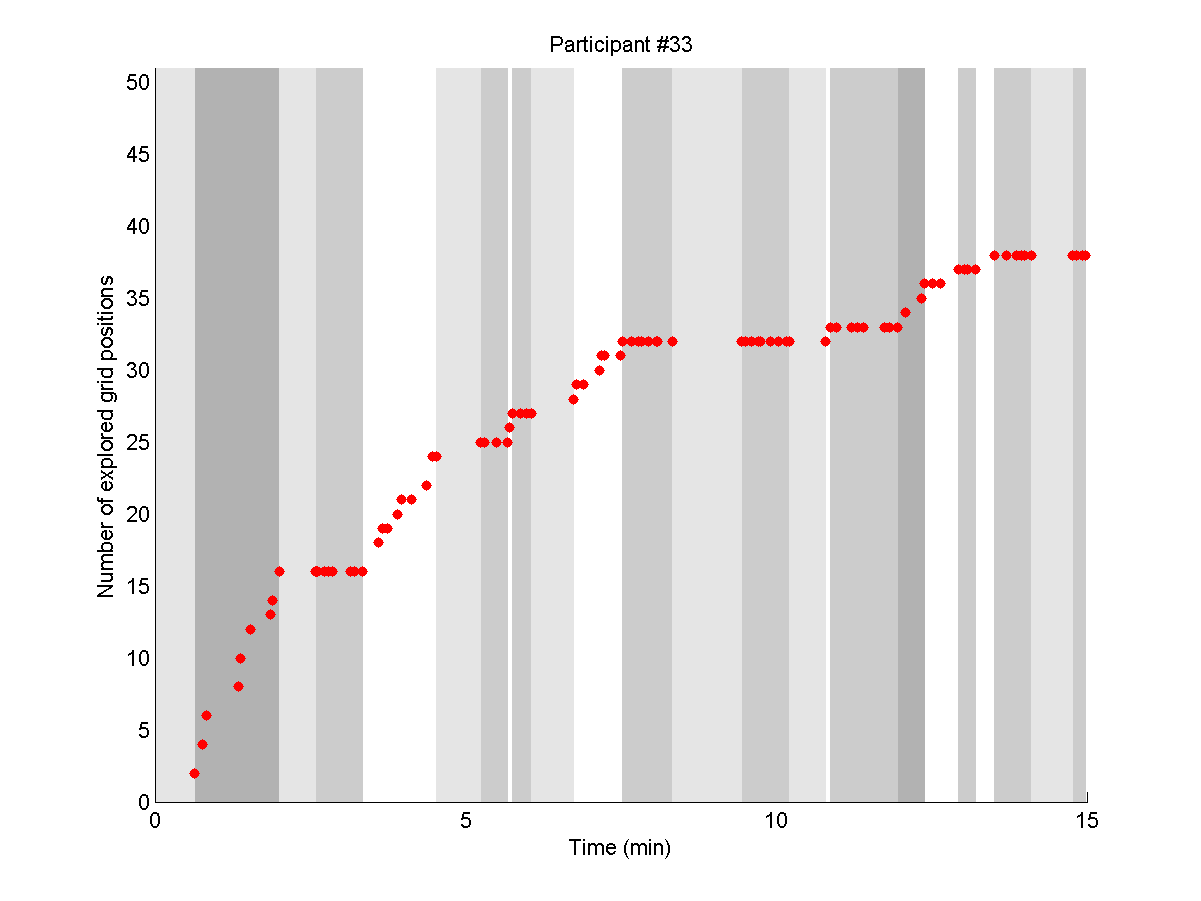

Supplement: Supplementary file 1 [file Presentation1.ZIP › individual plots/33.png]

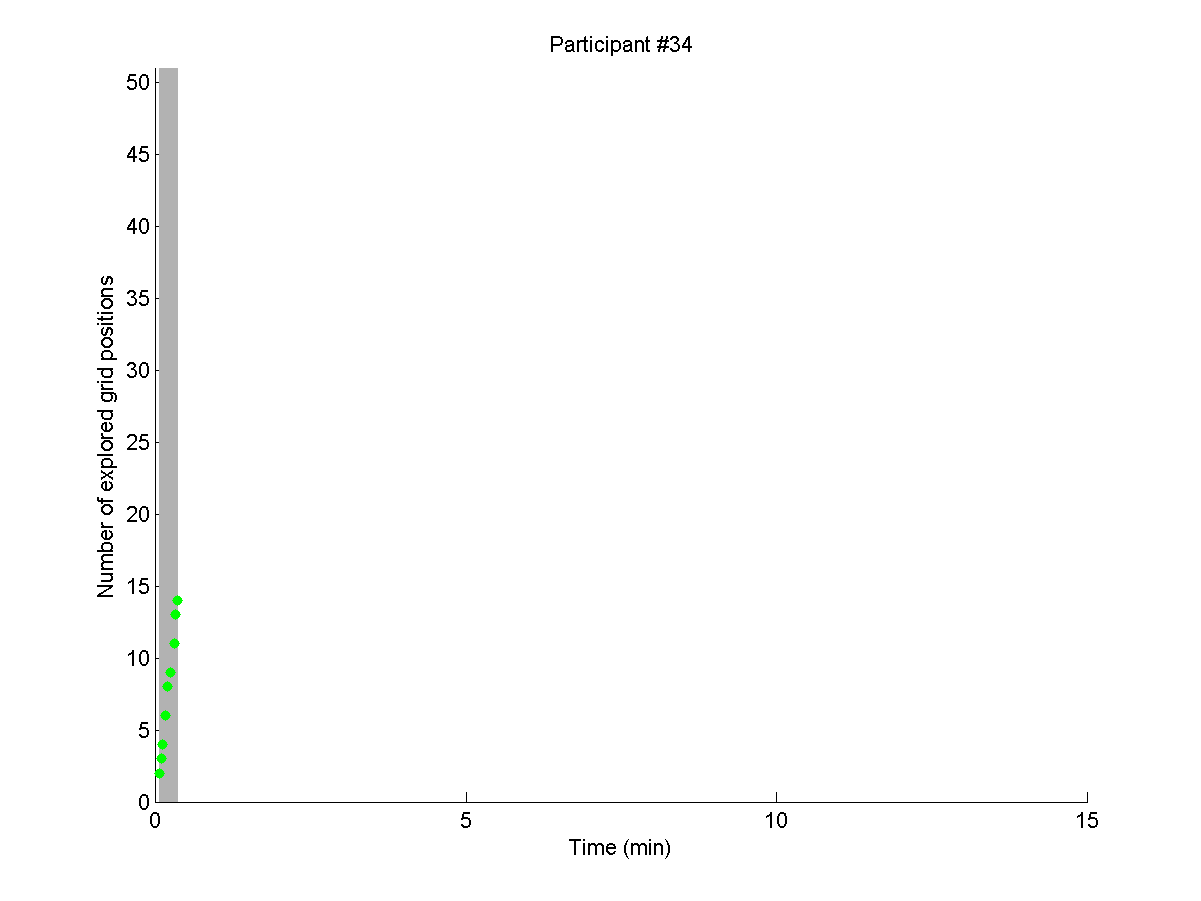

Supplement: Supplementary file 1 [file Presentation1.ZIP › individual plots/34.png]

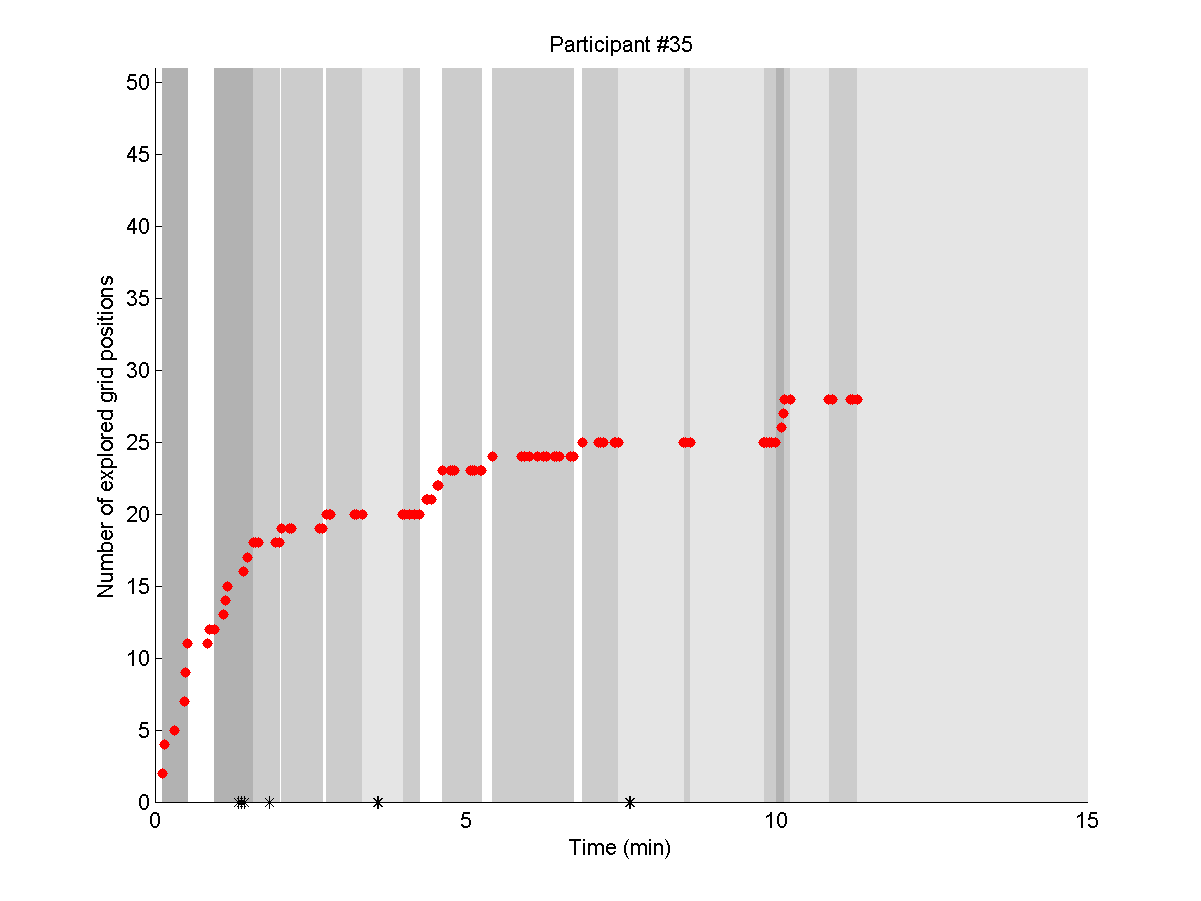

Supplement: Supplementary file 1 [file Presentation1.ZIP › individual plots/35.png]

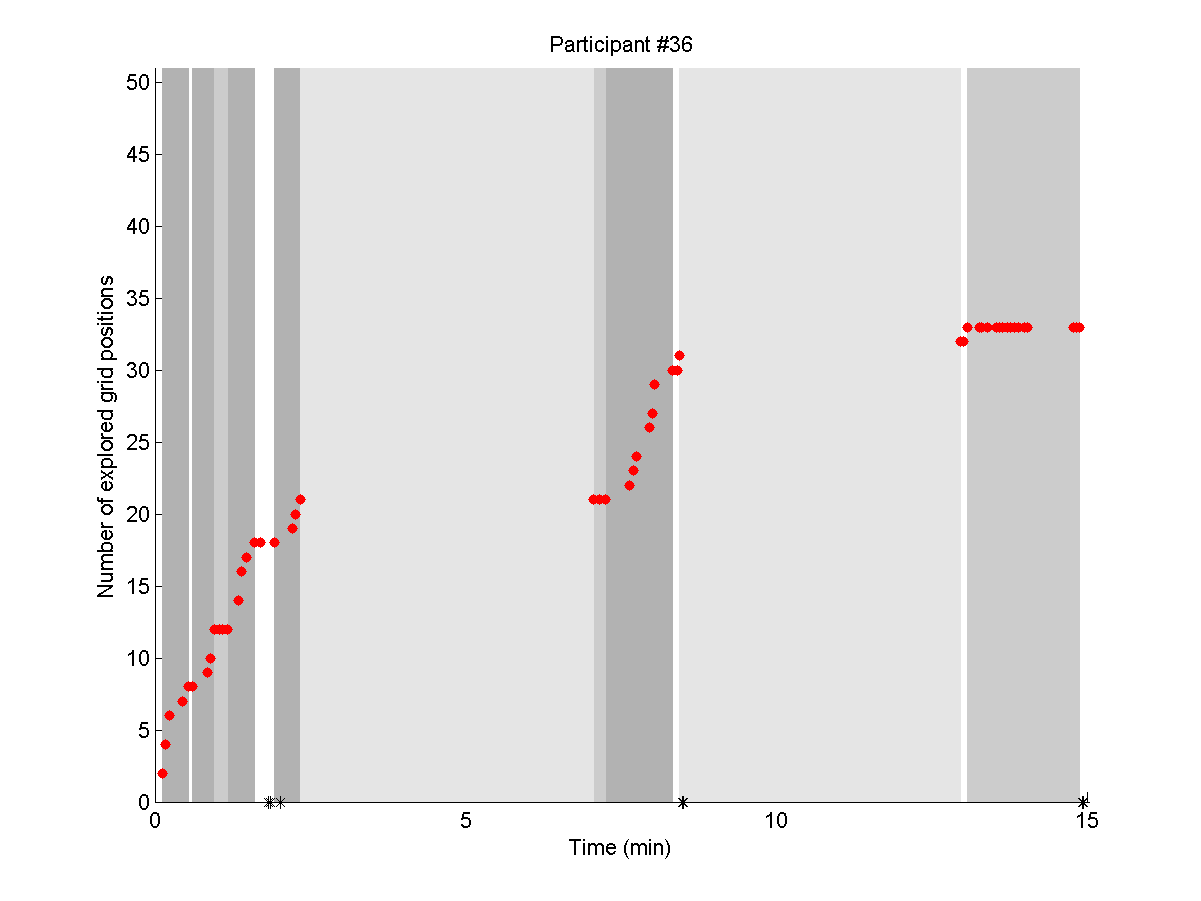

Supplement: Supplementary file 1 [file Presentation1.ZIP › individual plots/36.png]

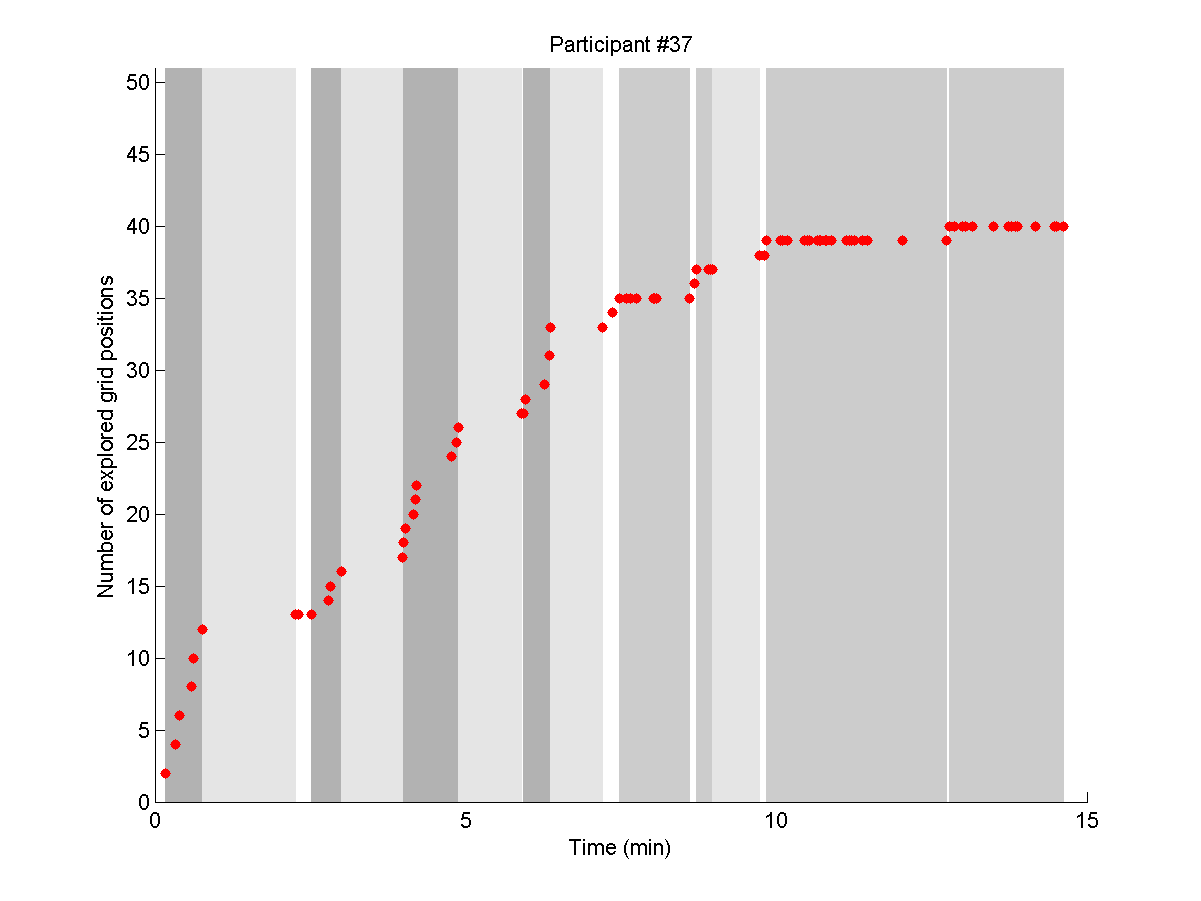

Supplement: Supplementary file 1 [file Presentation1.ZIP › individual plots/37.png]

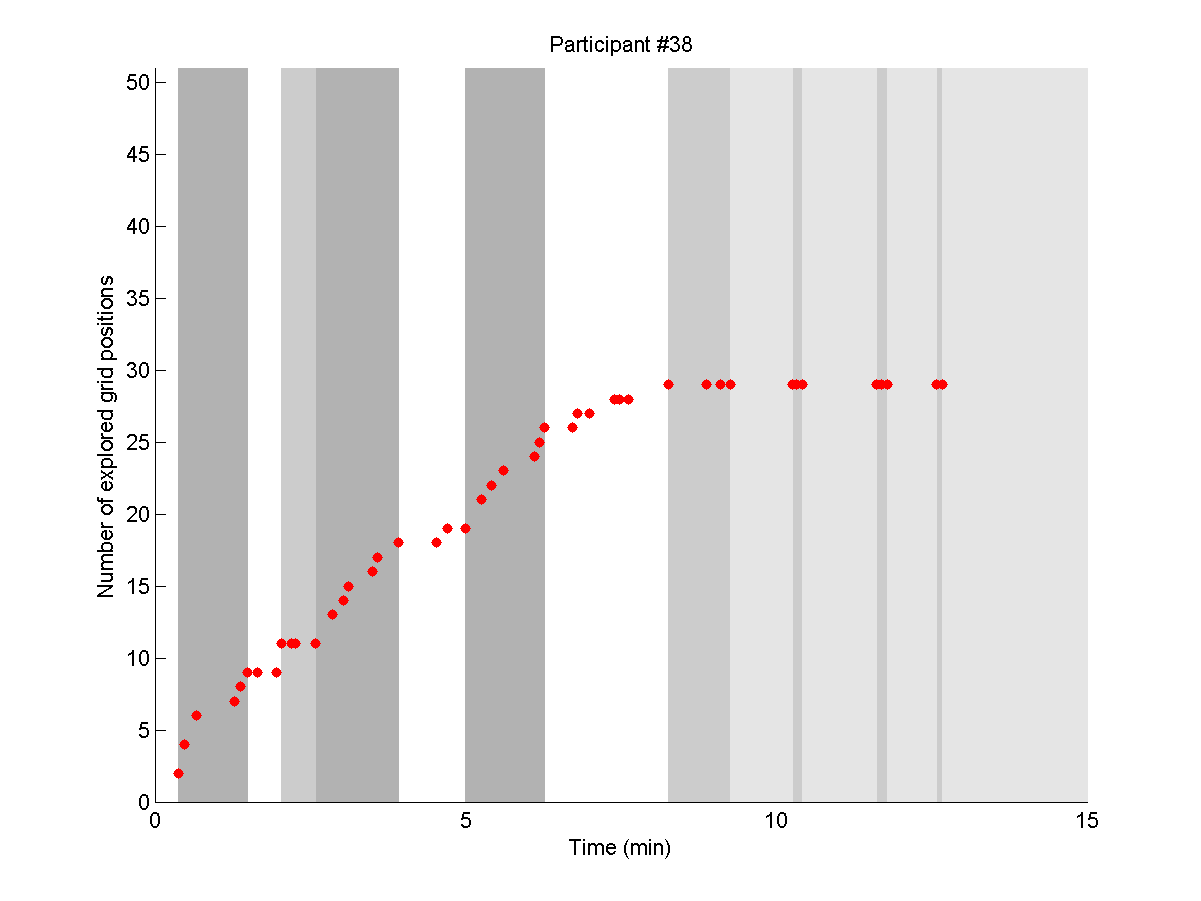

Supplement: Supplementary file 1 [file Presentation1.ZIP › individual plots/38.png]

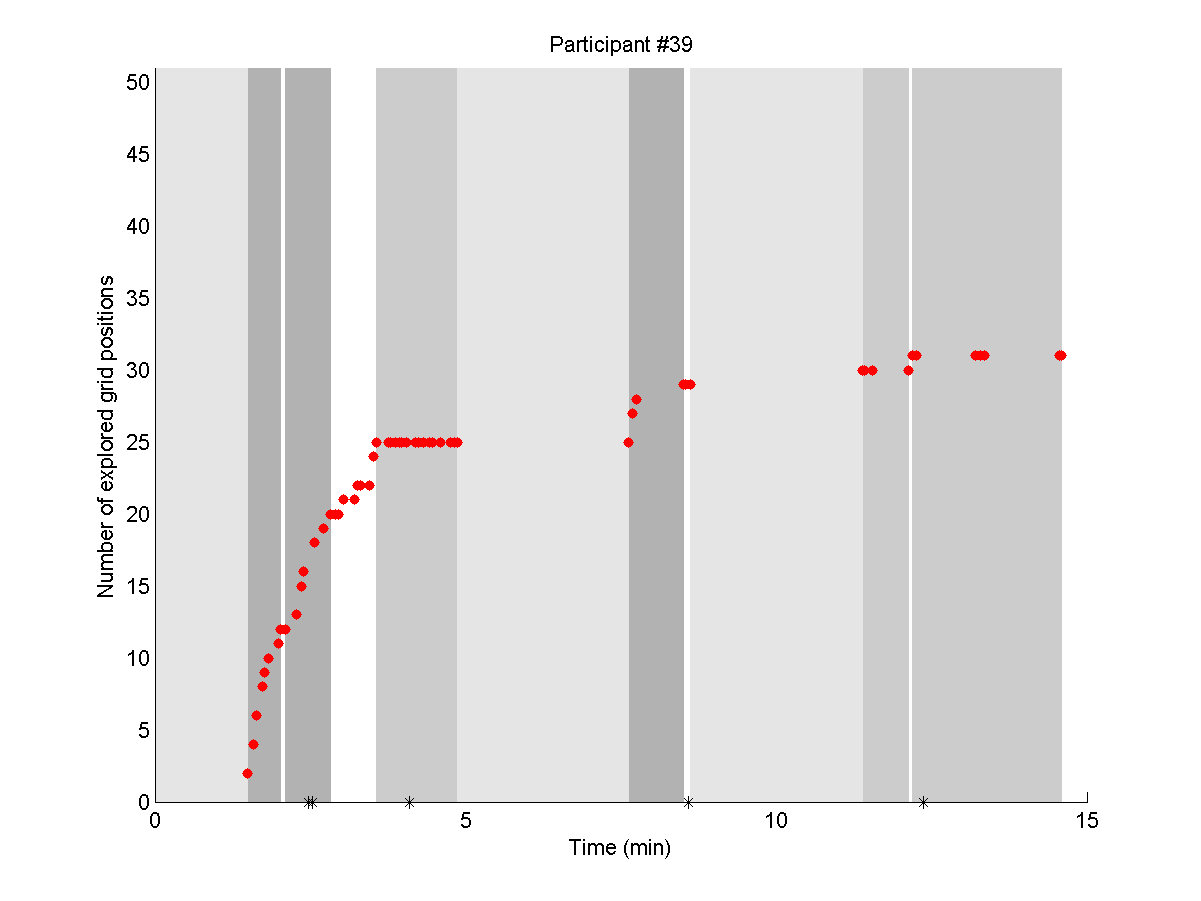

Supplement: Supplementary file 1 [file Presentation1.ZIP › individual plots/39.png]

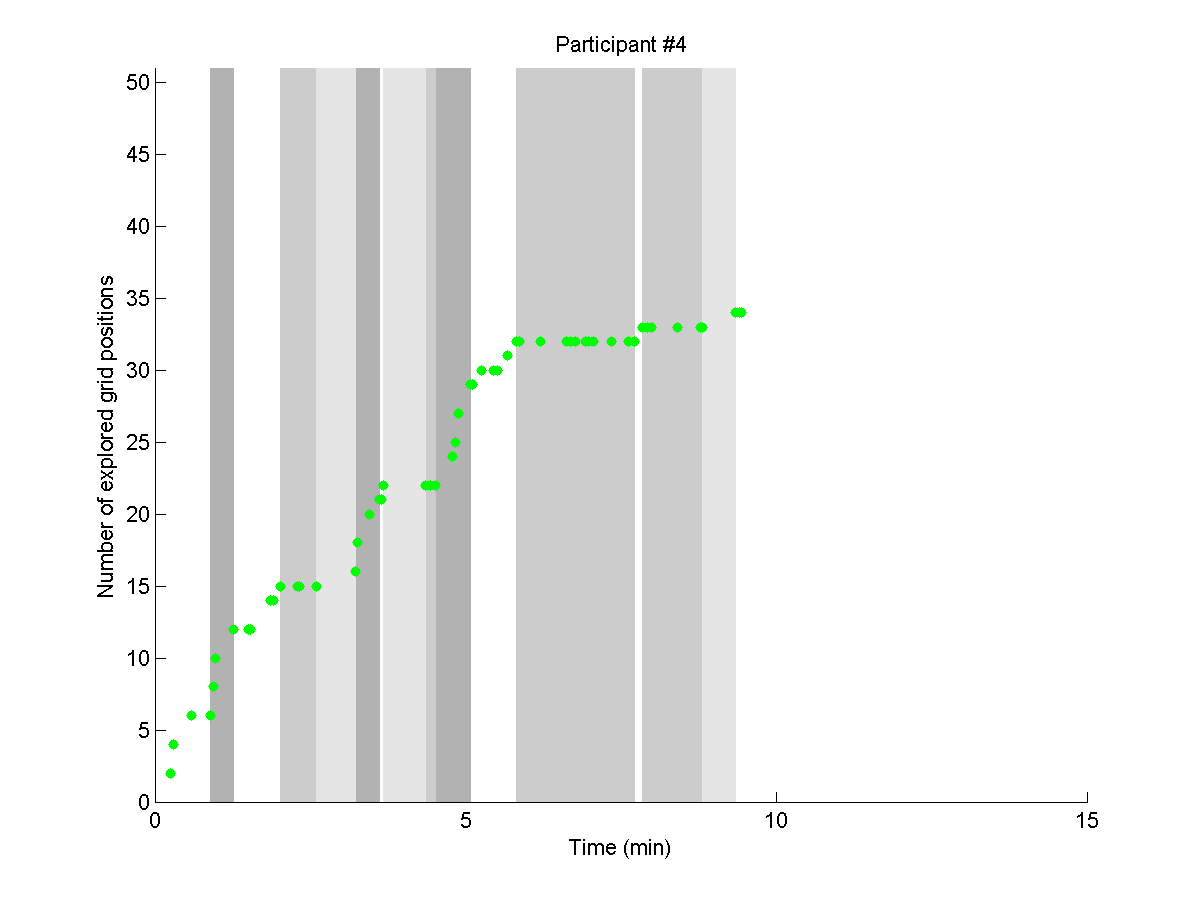

Supplement: Supplementary file 1 [file Presentation1.ZIP › individual plots/4.png]

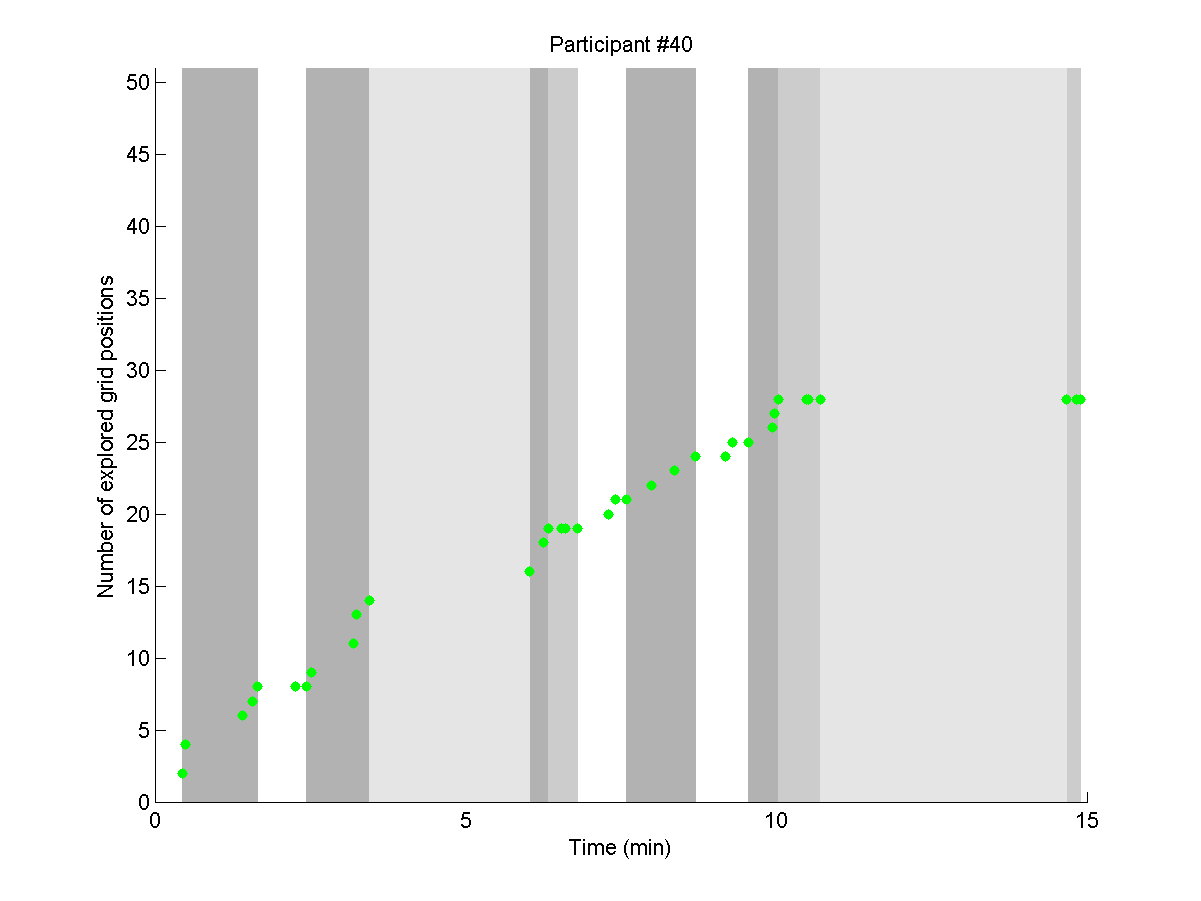

Supplement: Supplementary file 1 [file Presentation1.ZIP › individual plots/40.png]

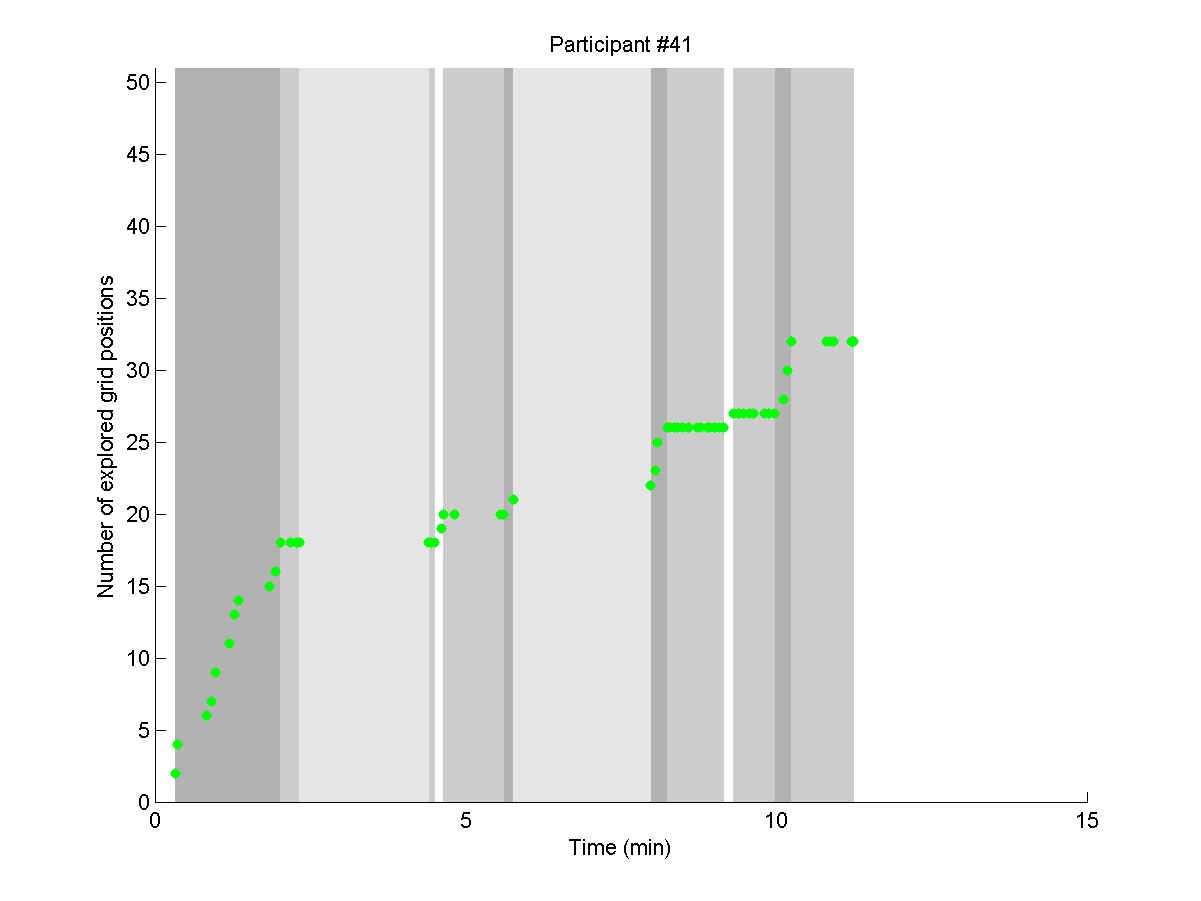

Supplement: Supplementary file 1 [file Presentation1.ZIP › individual plots/41.png]

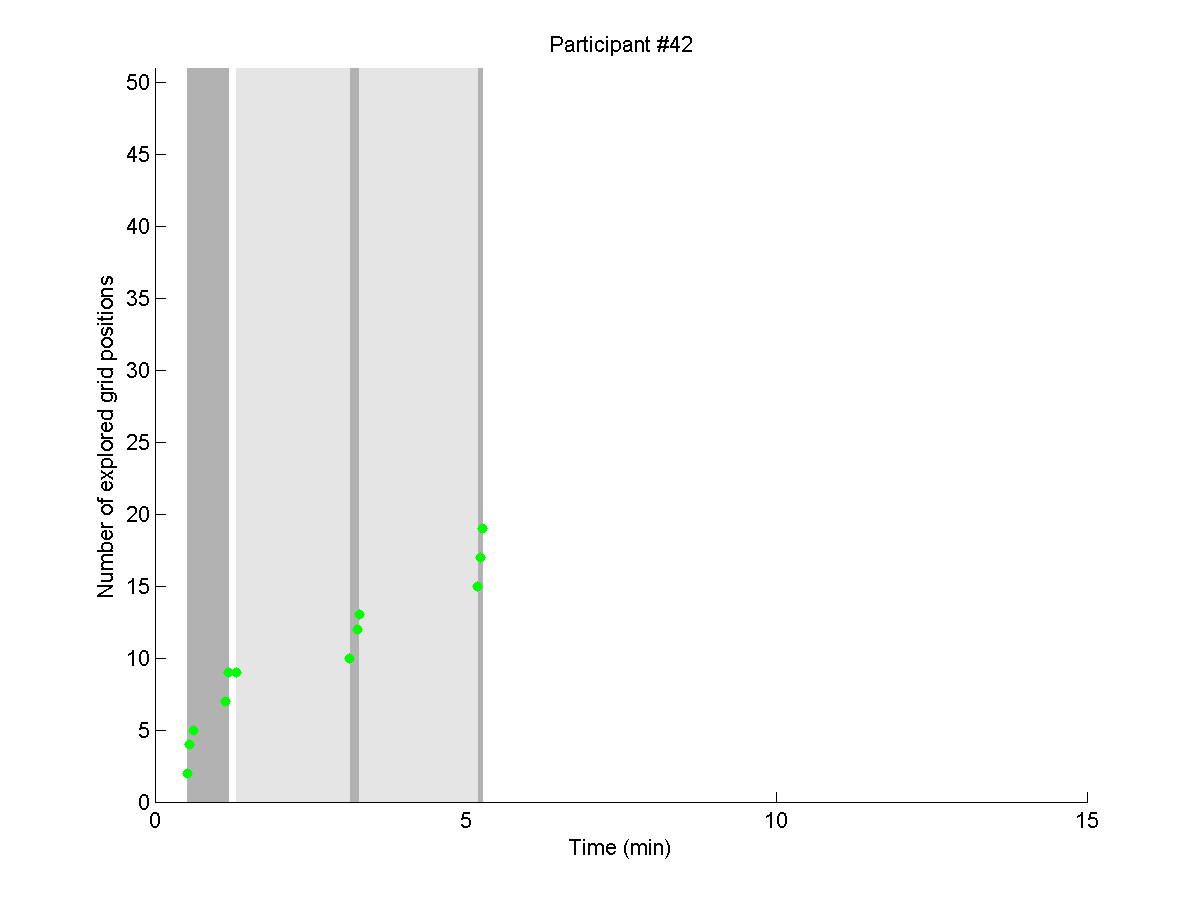

Supplement: Supplementary file 1 [file Presentation1.ZIP › individual plots/42.png]

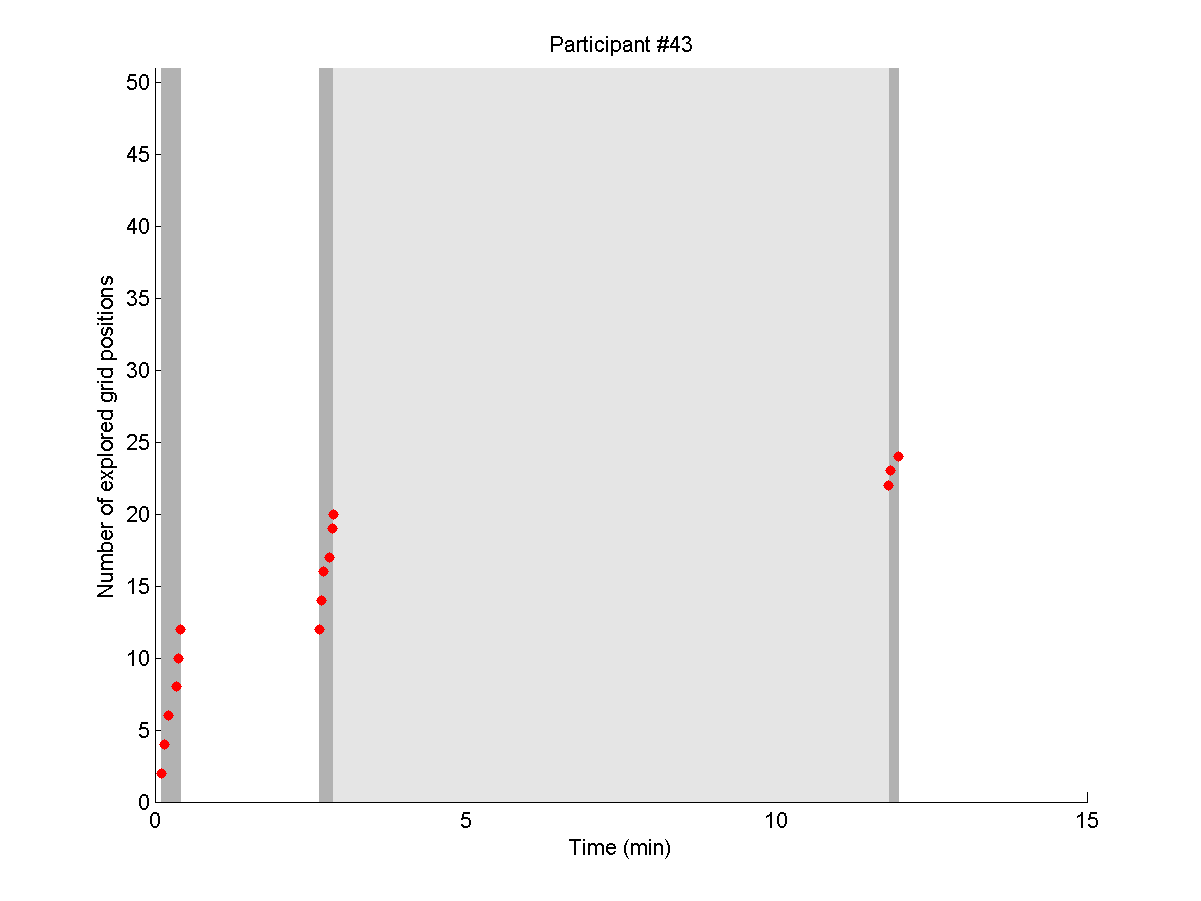

Supplement: Supplementary file 1 [file Presentation1.ZIP › individual plots/43.png]

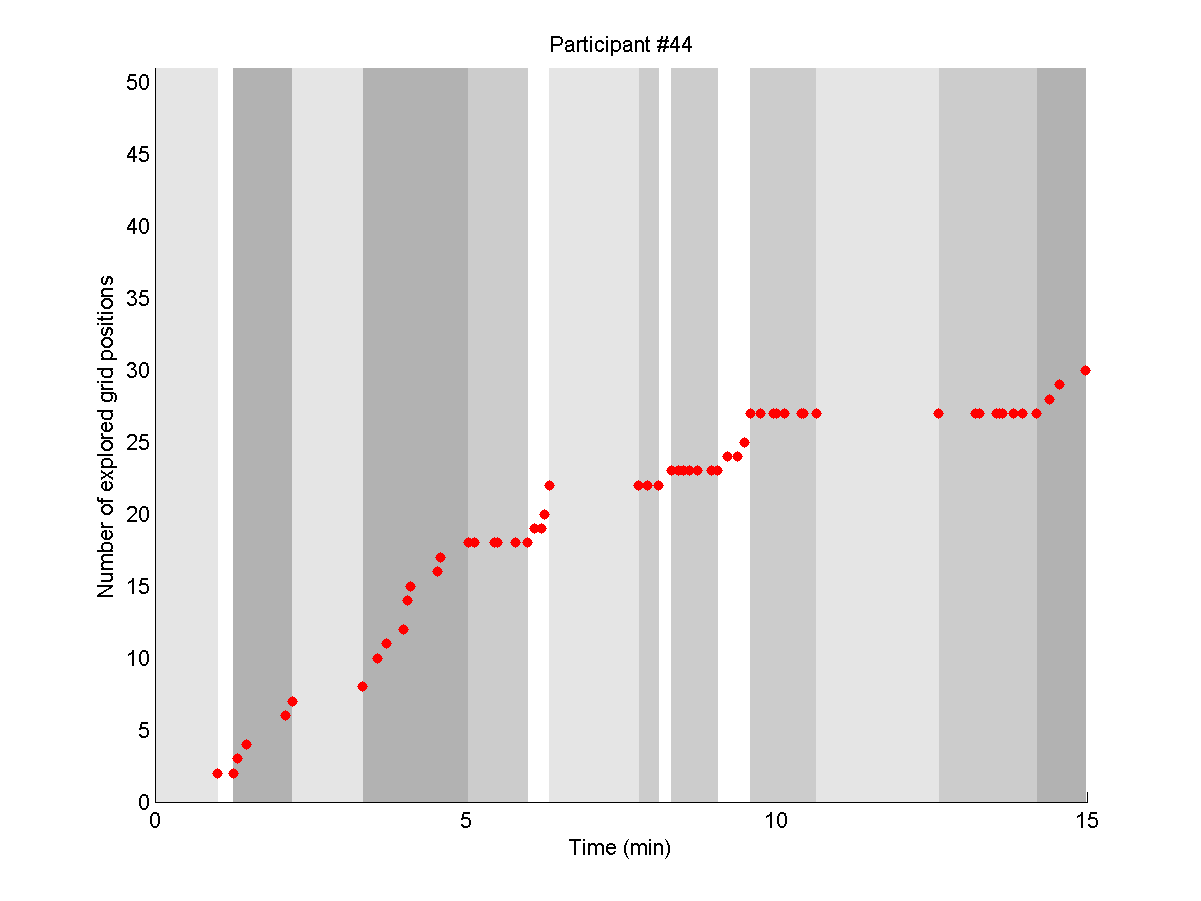

Supplement: Supplementary file 1 [file Presentation1.ZIP › individual plots/44.png]

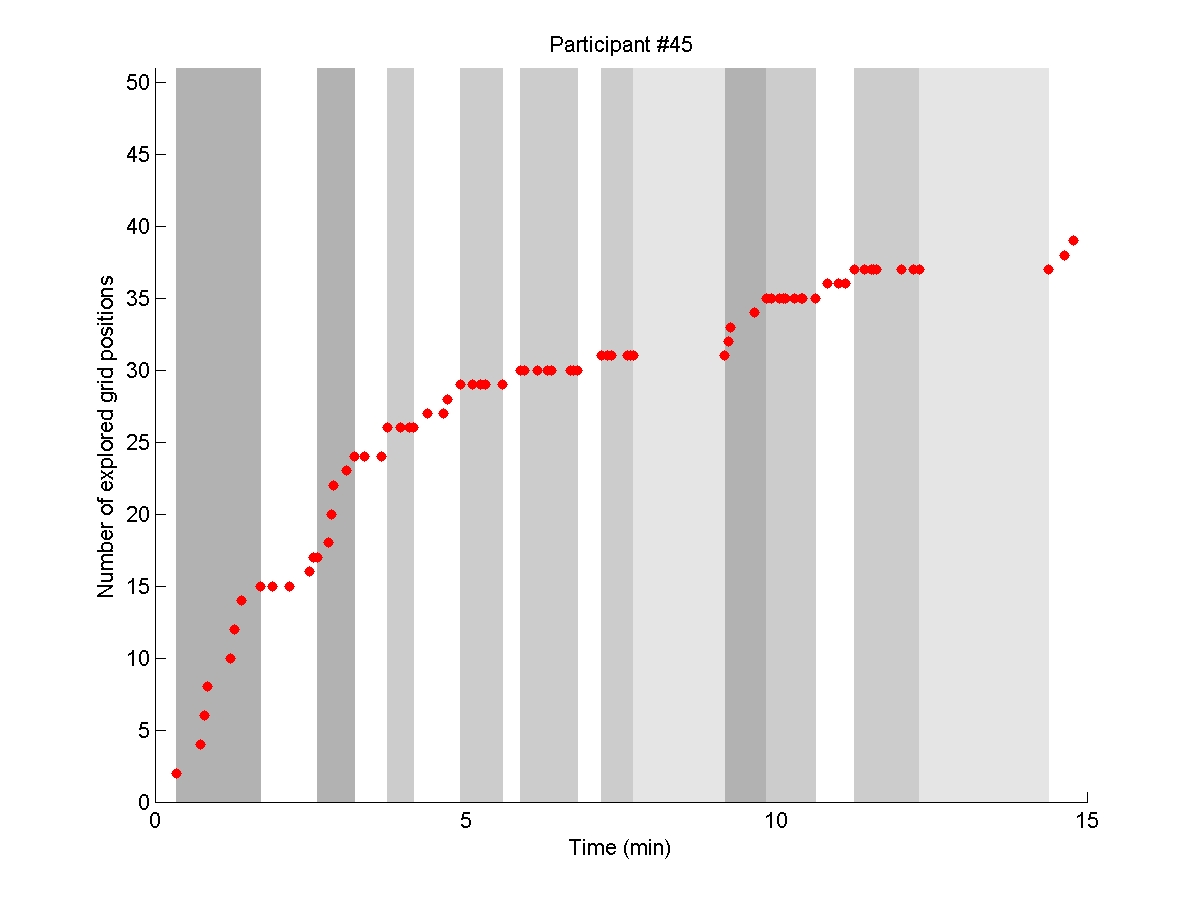

Supplement: Supplementary file 1 [file Presentation1.ZIP › individual plots/45.png]

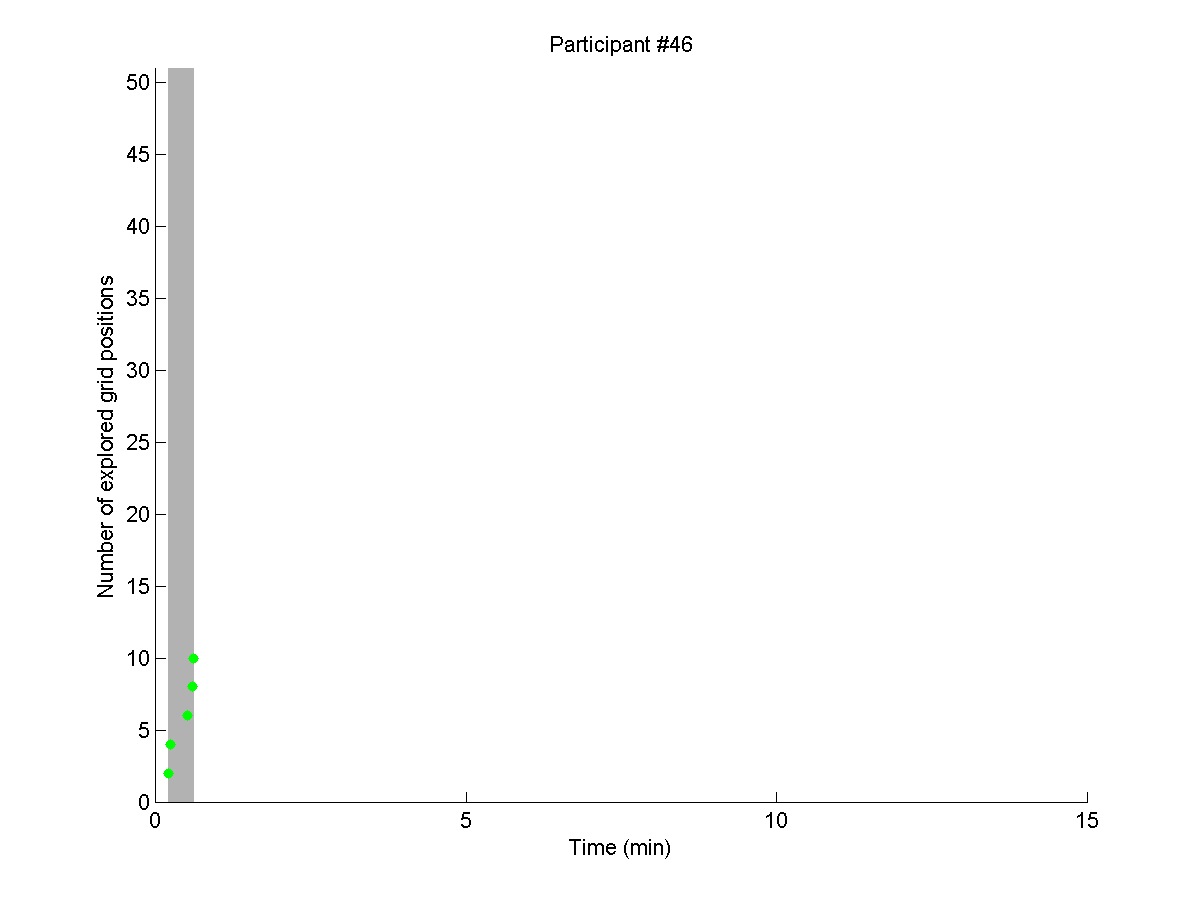

Supplement: Supplementary file 1 [file Presentation1.ZIP › individual plots/46.png]

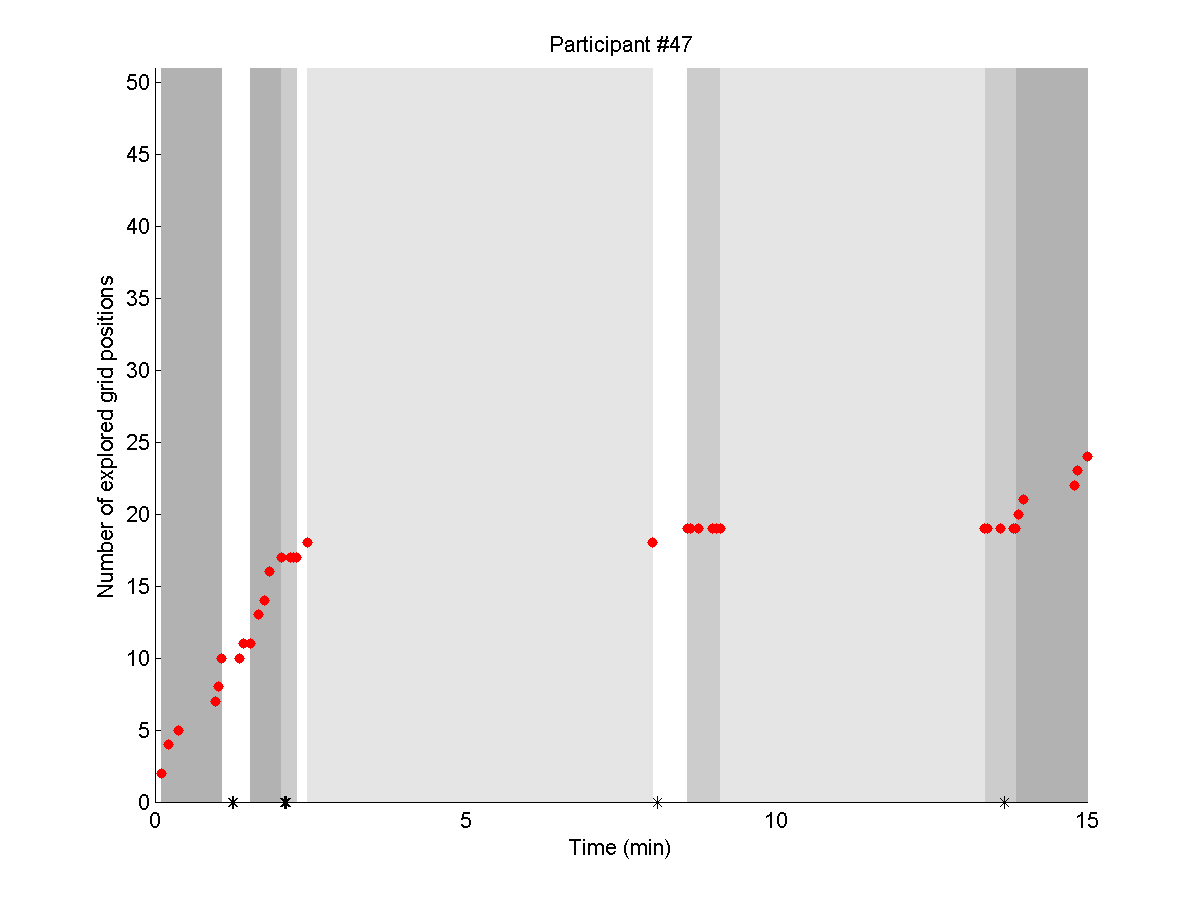

Supplement: Supplementary file 1 [file Presentation1.ZIP › individual plots/47.png]

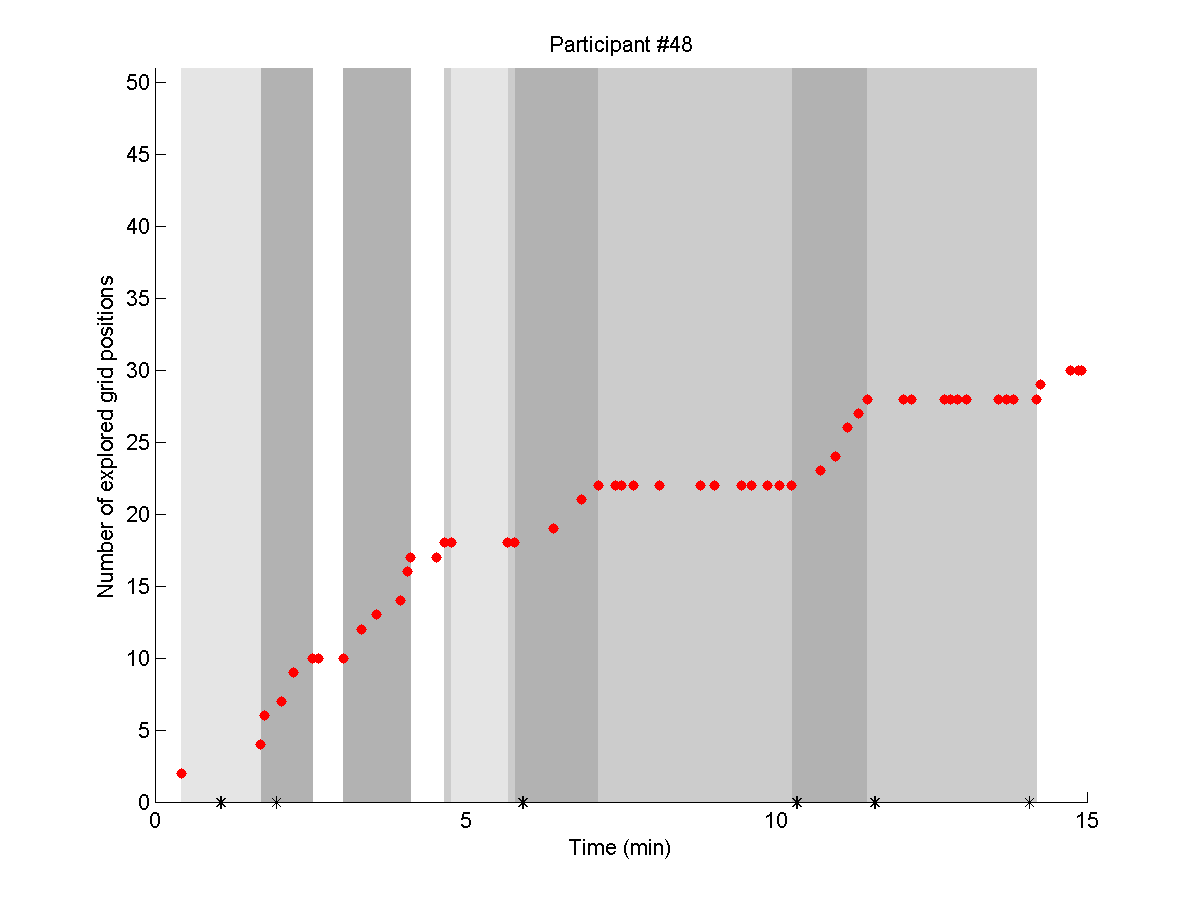

Supplement: Supplementary file 1 [file Presentation1.ZIP › individual plots/48.png]

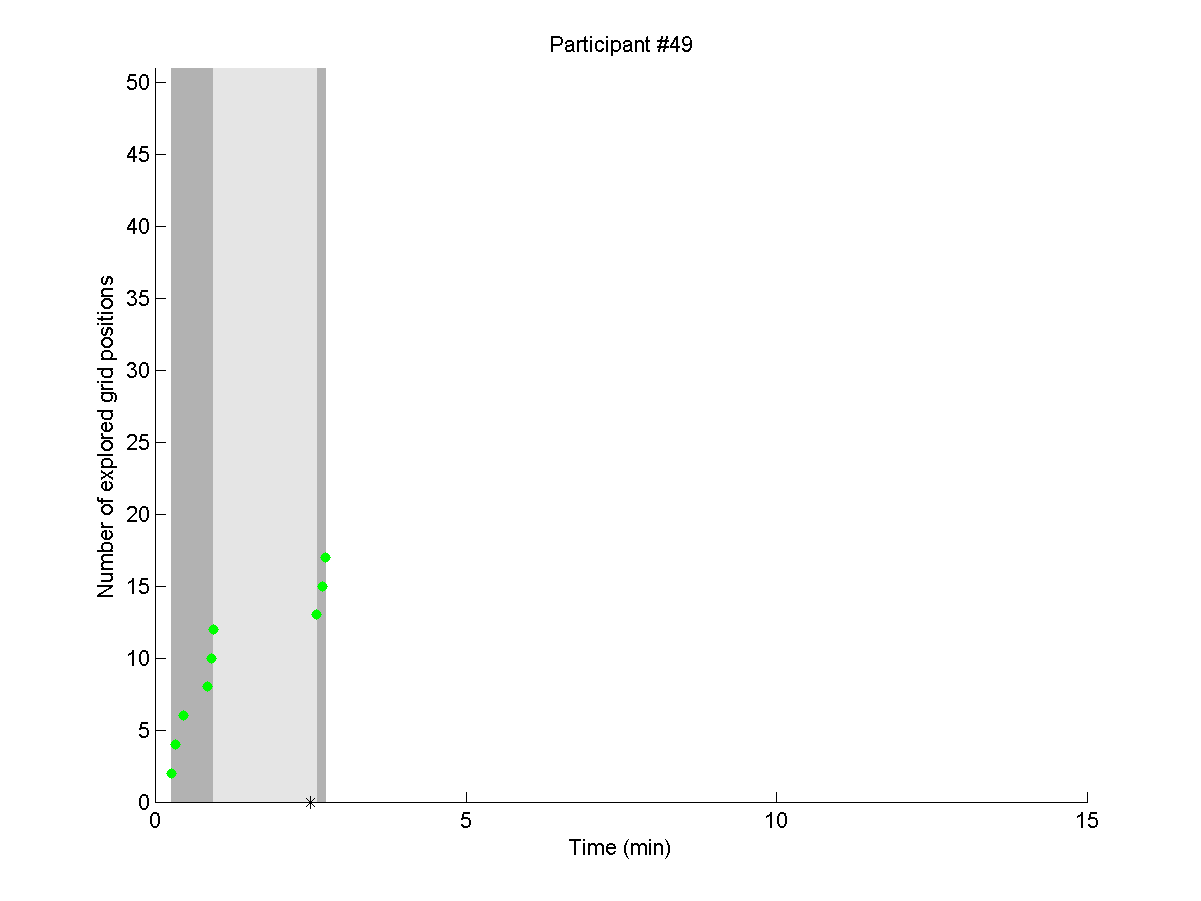

Supplement: Supplementary file 1 [file Presentation1.ZIP › individual plots/49.png]

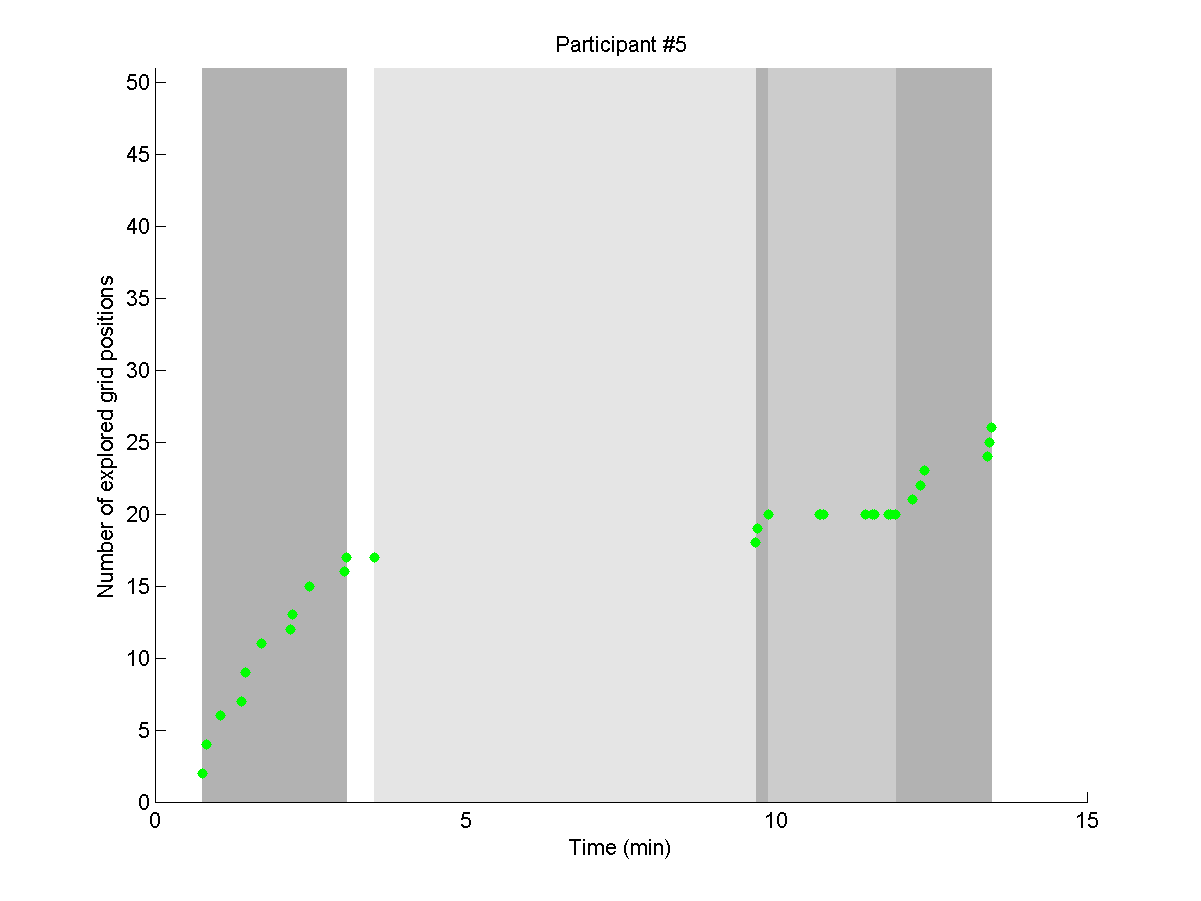

Supplement: Supplementary file 1 [file Presentation1.ZIP › individual plots/5.png]

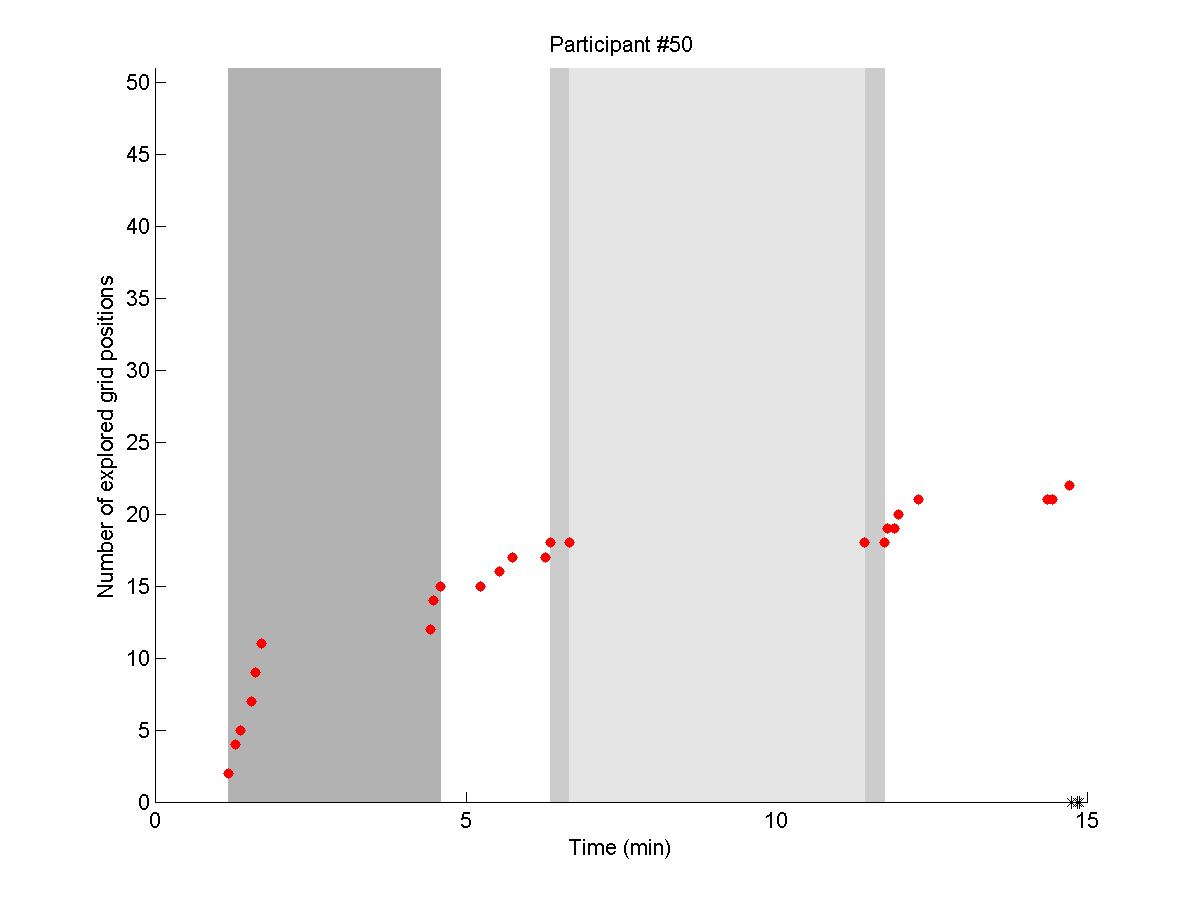

Supplement: Supplementary file 1 [file Presentation1.ZIP › individual plots/50.png]

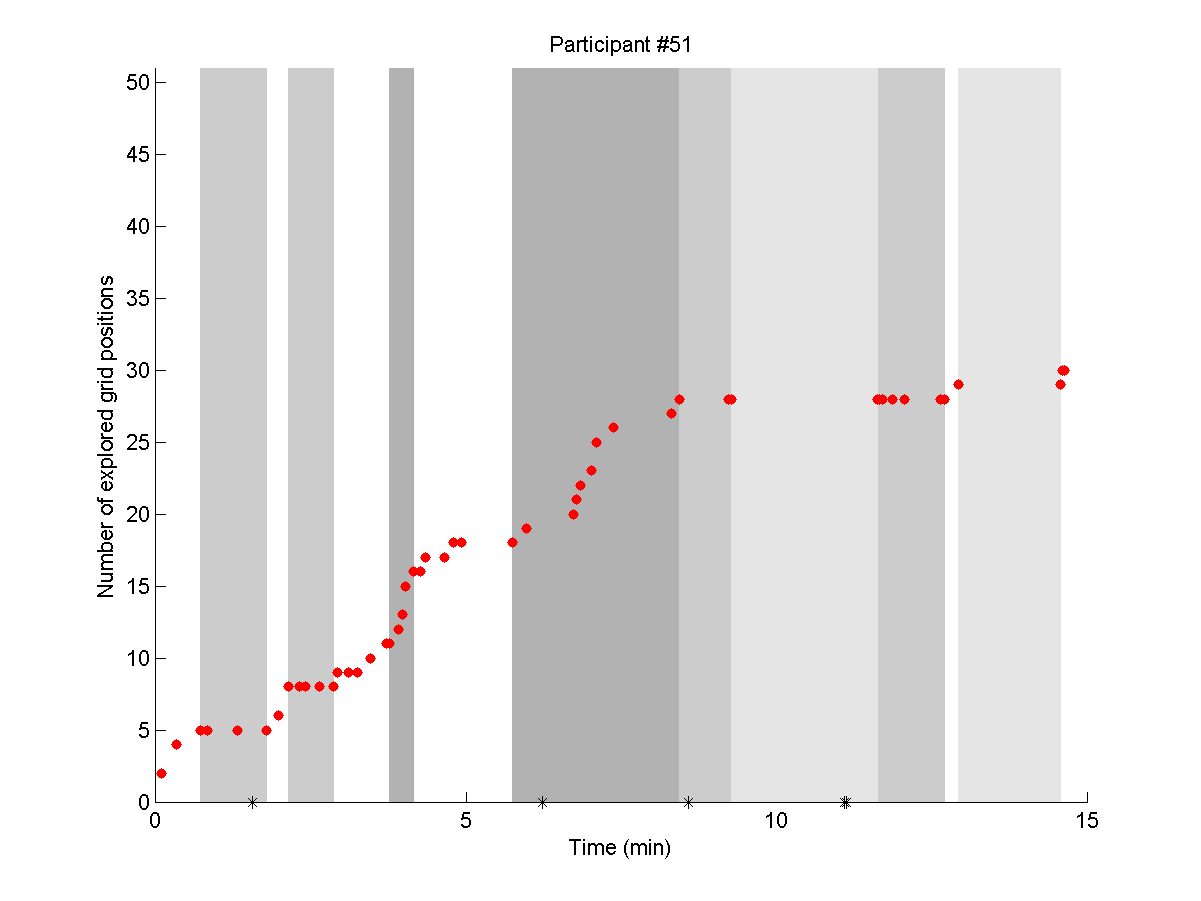

Supplement: Supplementary file 1 [file Presentation1.ZIP › individual plots/51.png]

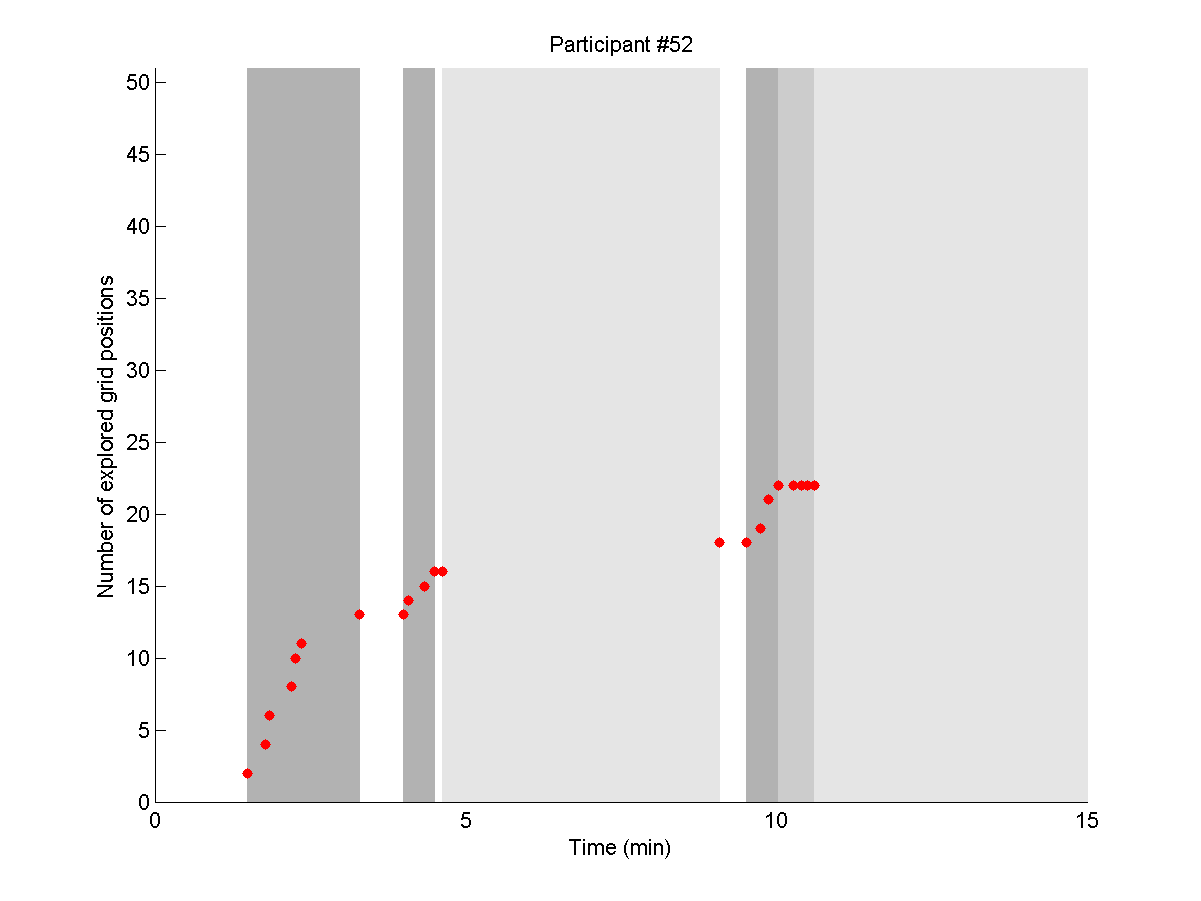

Supplement: Supplementary file 1 [file Presentation1.ZIP › individual plots/52.png]

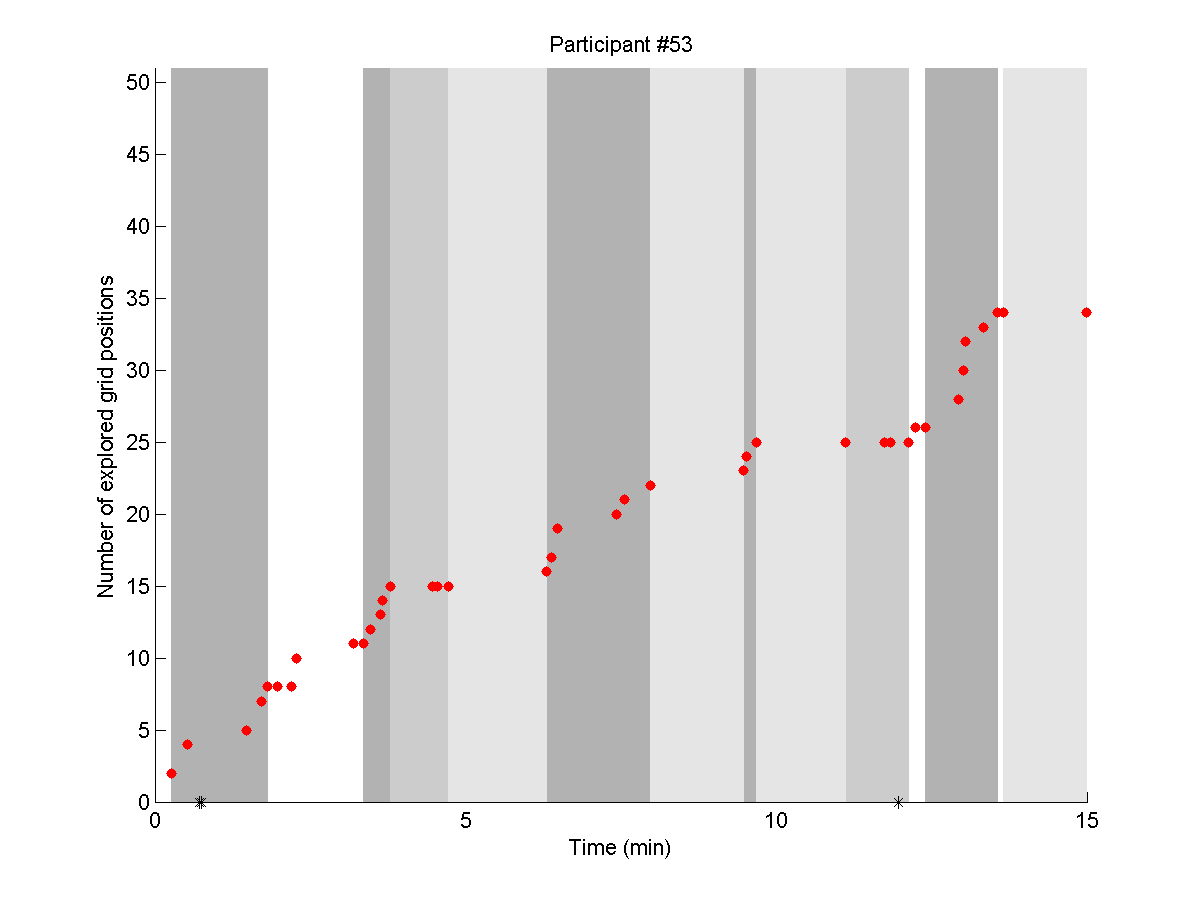

Supplement: Supplementary file 1 [file Presentation1.ZIP › individual plots/53.png]

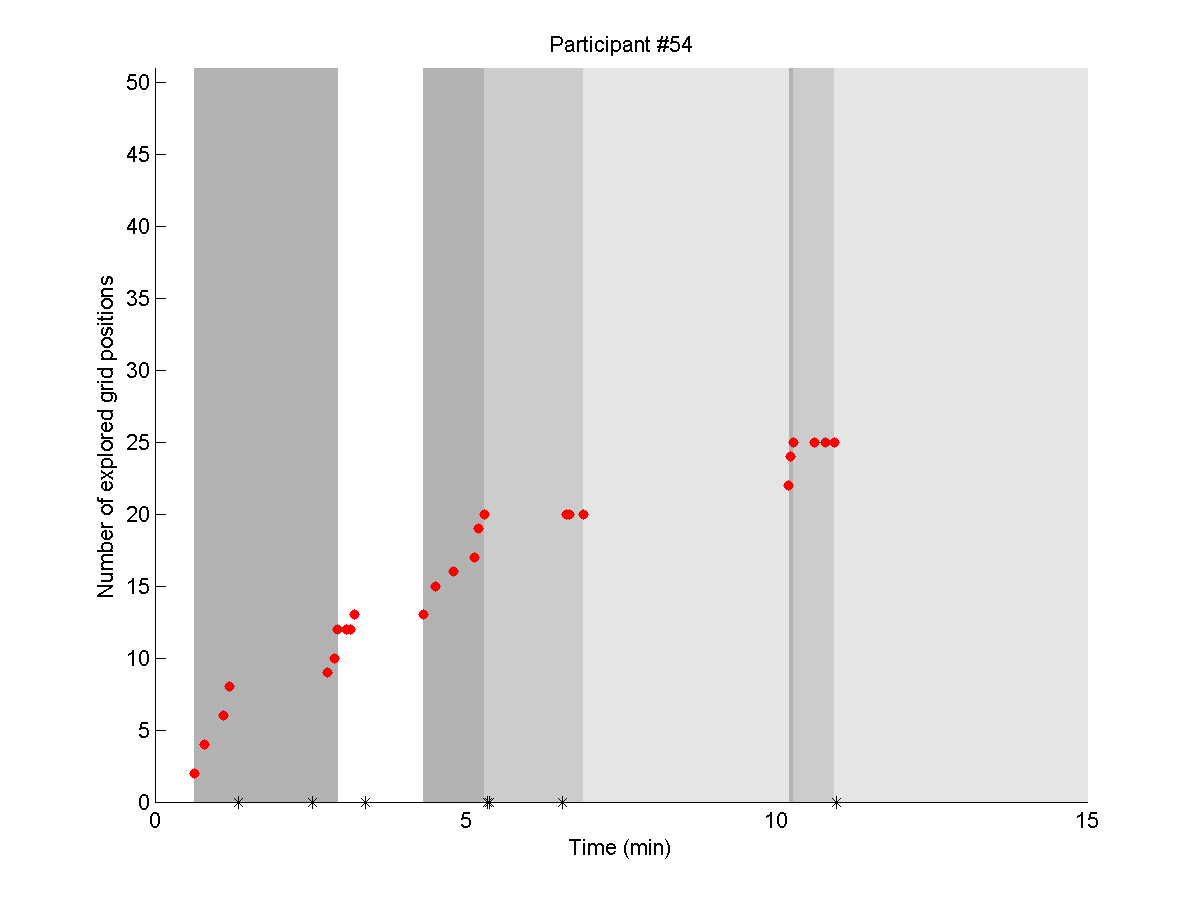

Supplement: Supplementary file 1 [file Presentation1.ZIP › individual plots/54.png]

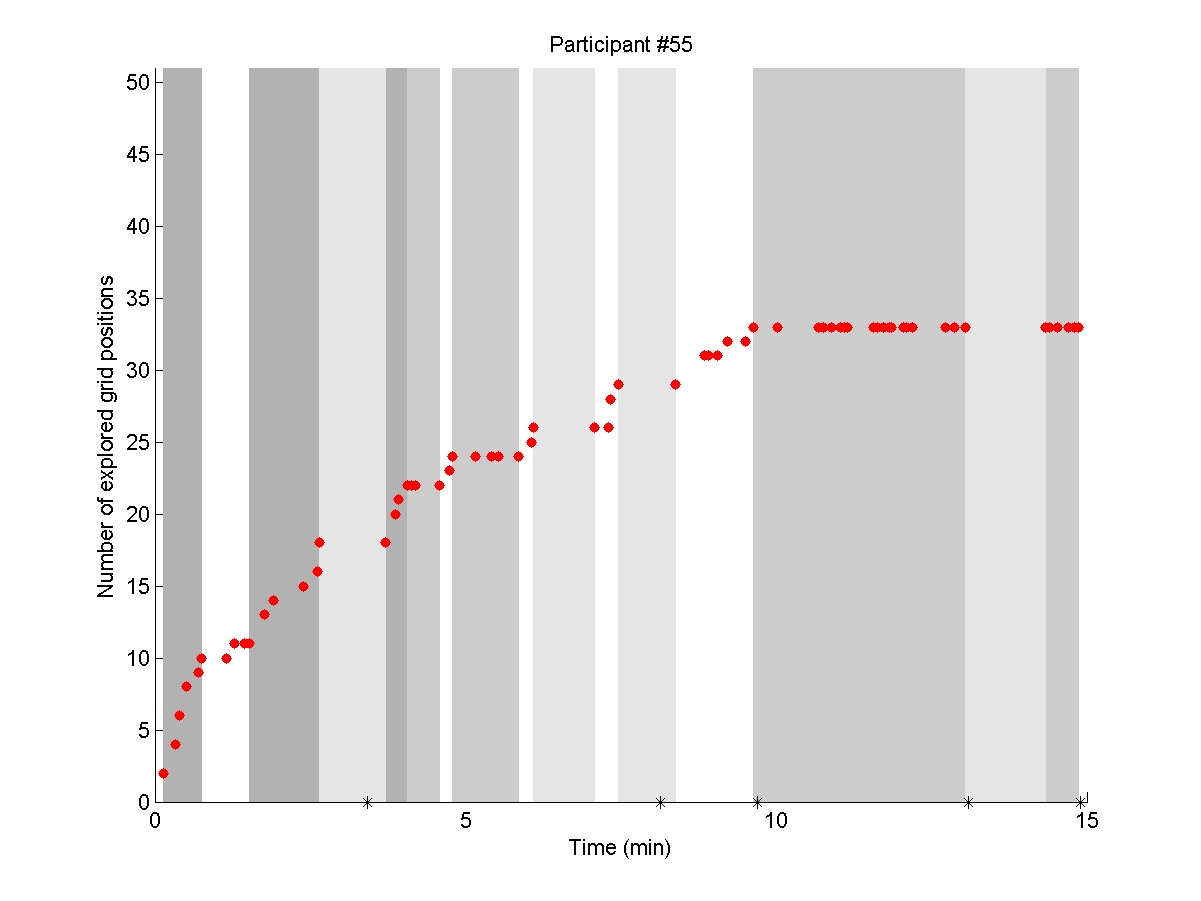

Supplement: Supplementary file 1 [file Presentation1.ZIP › individual plots/55.png]

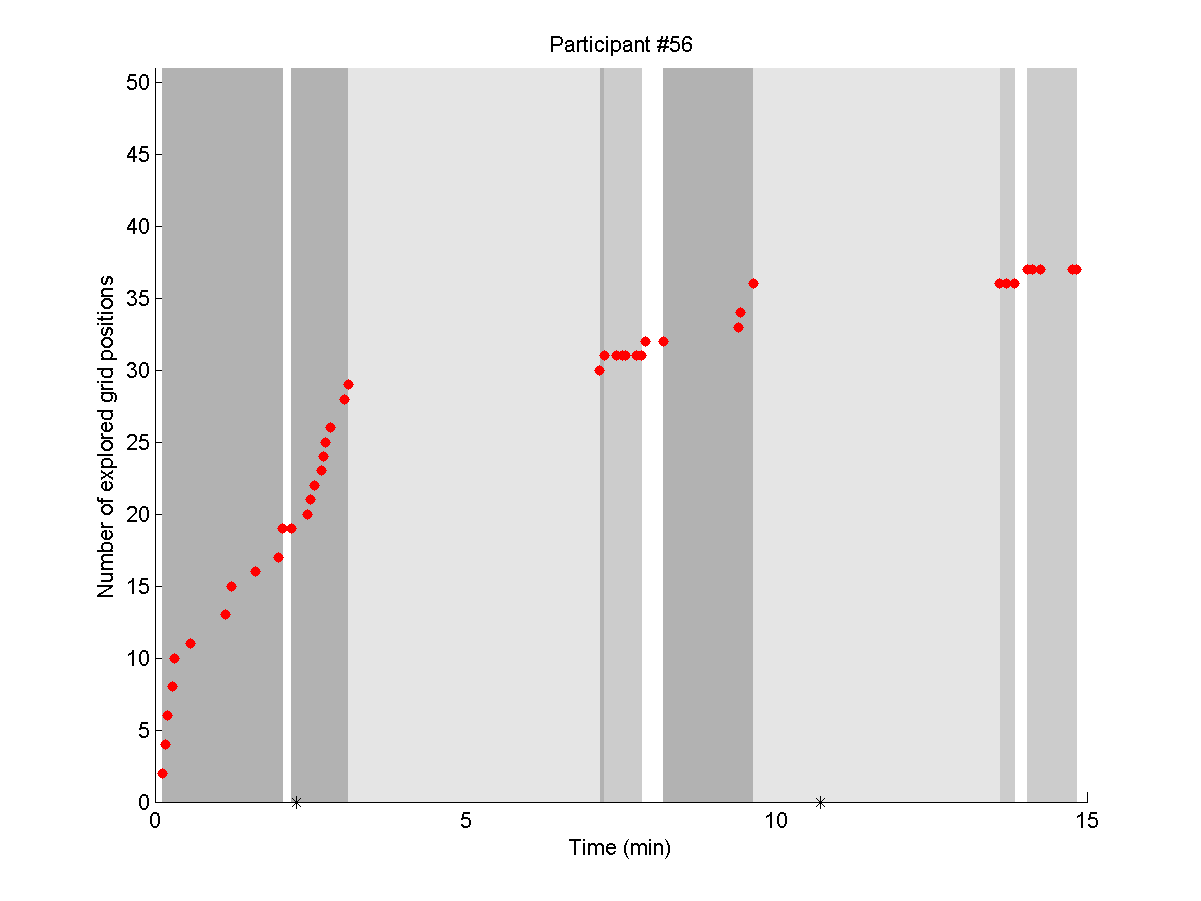

Supplement: Supplementary file 1 [file Presentation1.ZIP › individual plots/56.png]

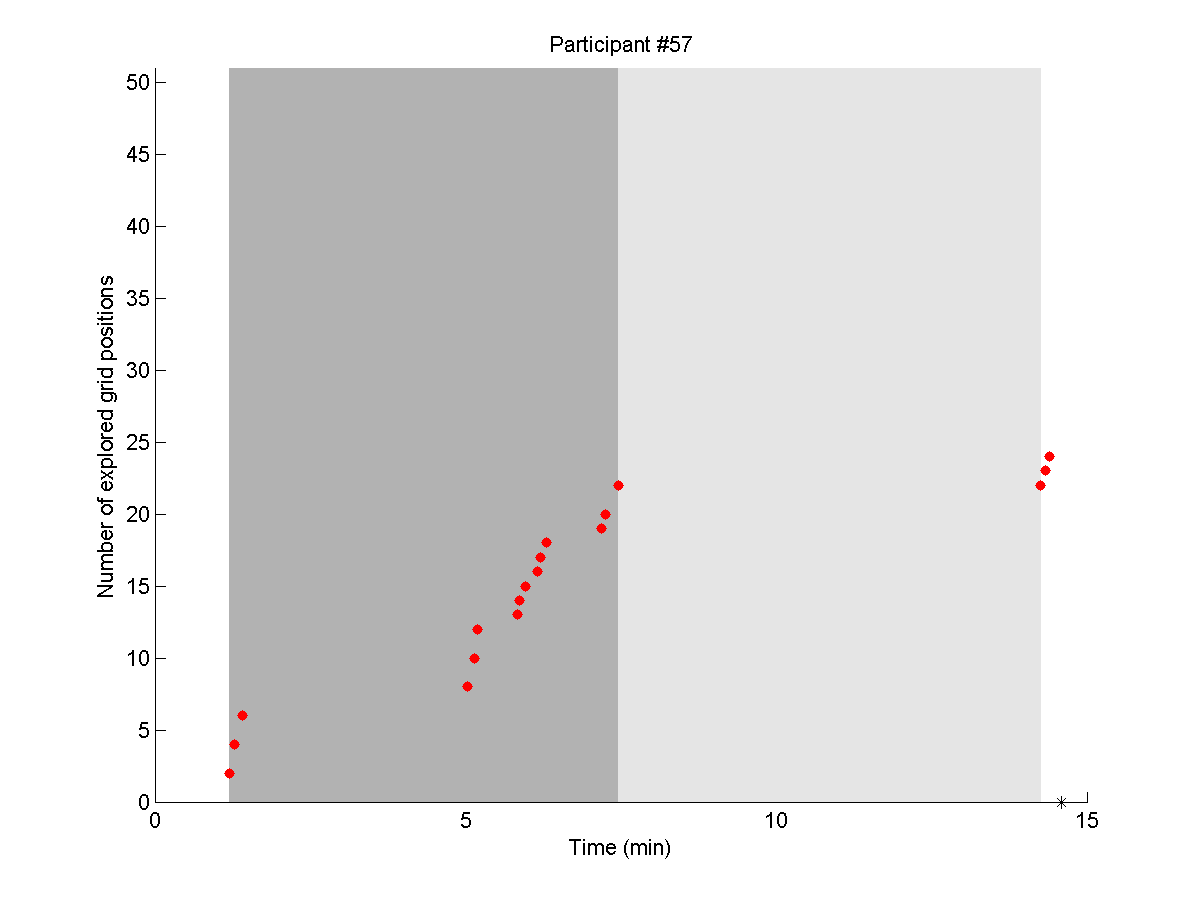

Supplement: Supplementary file 1 [file Presentation1.ZIP › individual plots/57.png]

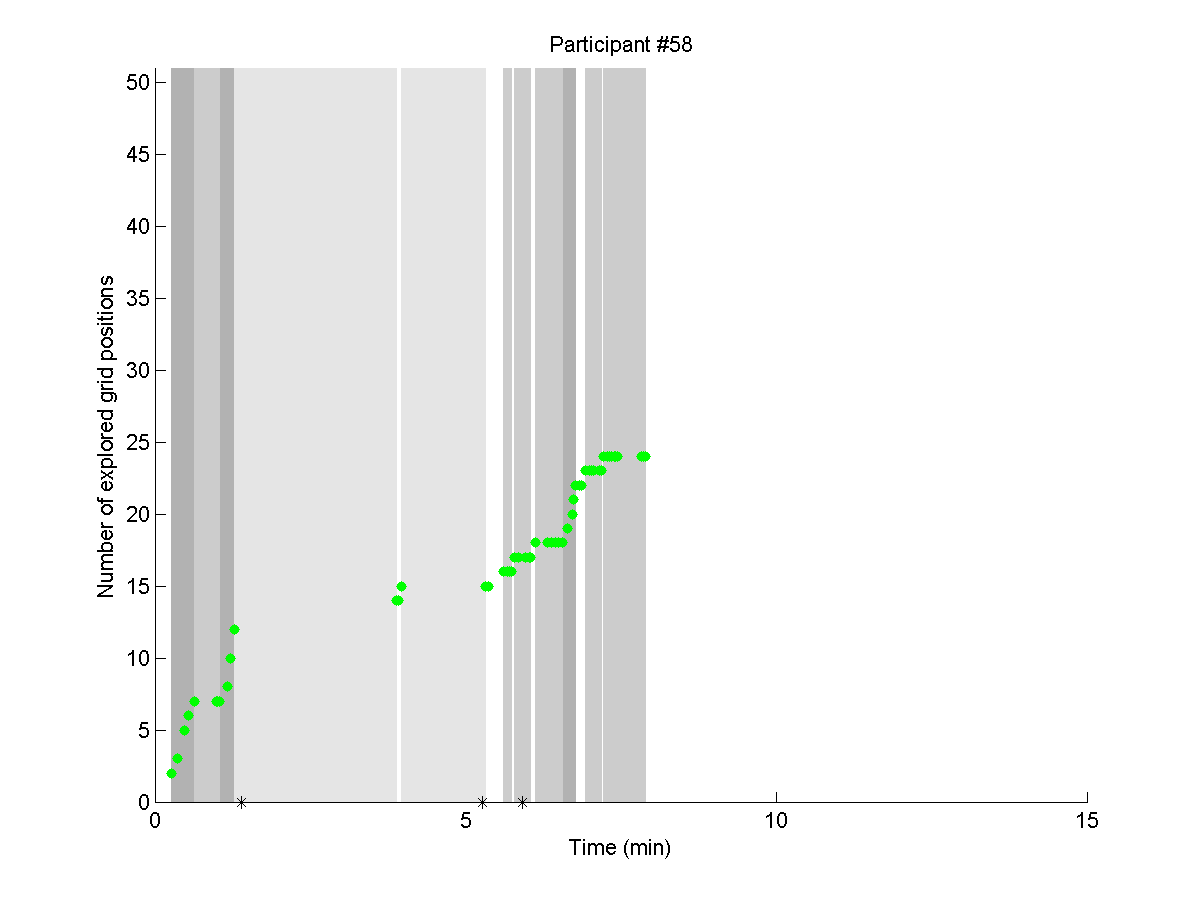

Supplement: Supplementary file 1 [file Presentation1.ZIP › individual plots/58.png]

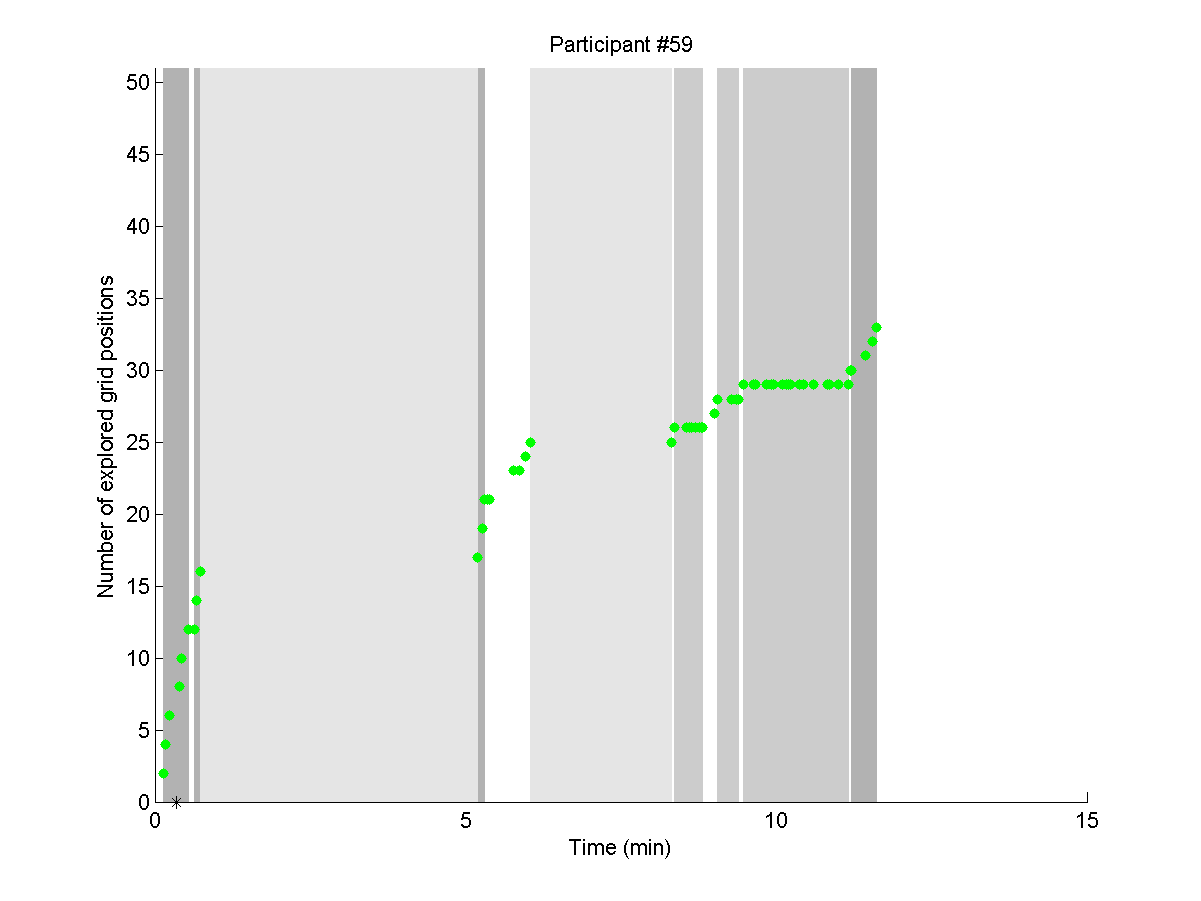

Supplement: Supplementary file 1 [file Presentation1.ZIP › individual plots/59.png]

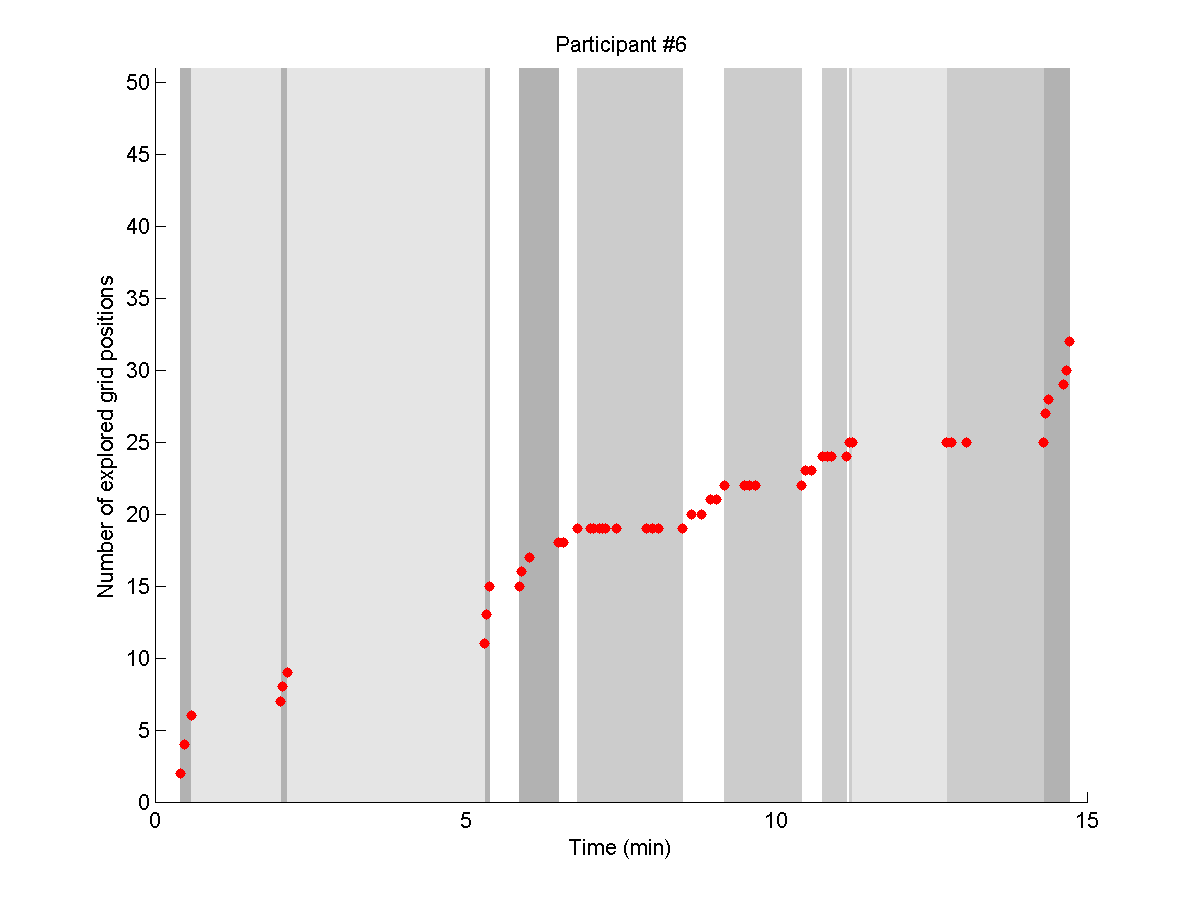

Supplement: Supplementary file 1 [file Presentation1.ZIP › individual plots/6.png]

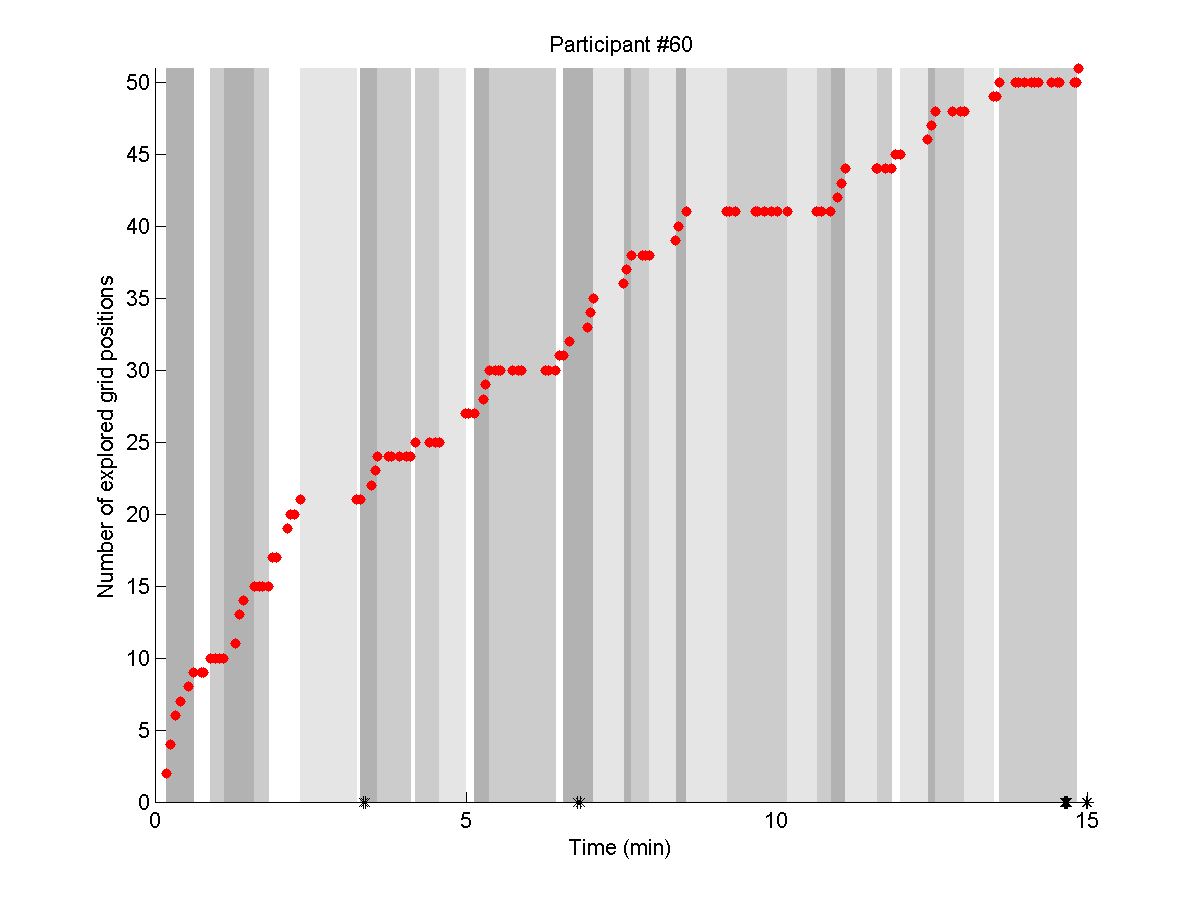

Supplement: Supplementary file 1 [file Presentation1.ZIP › individual plots/60.png]

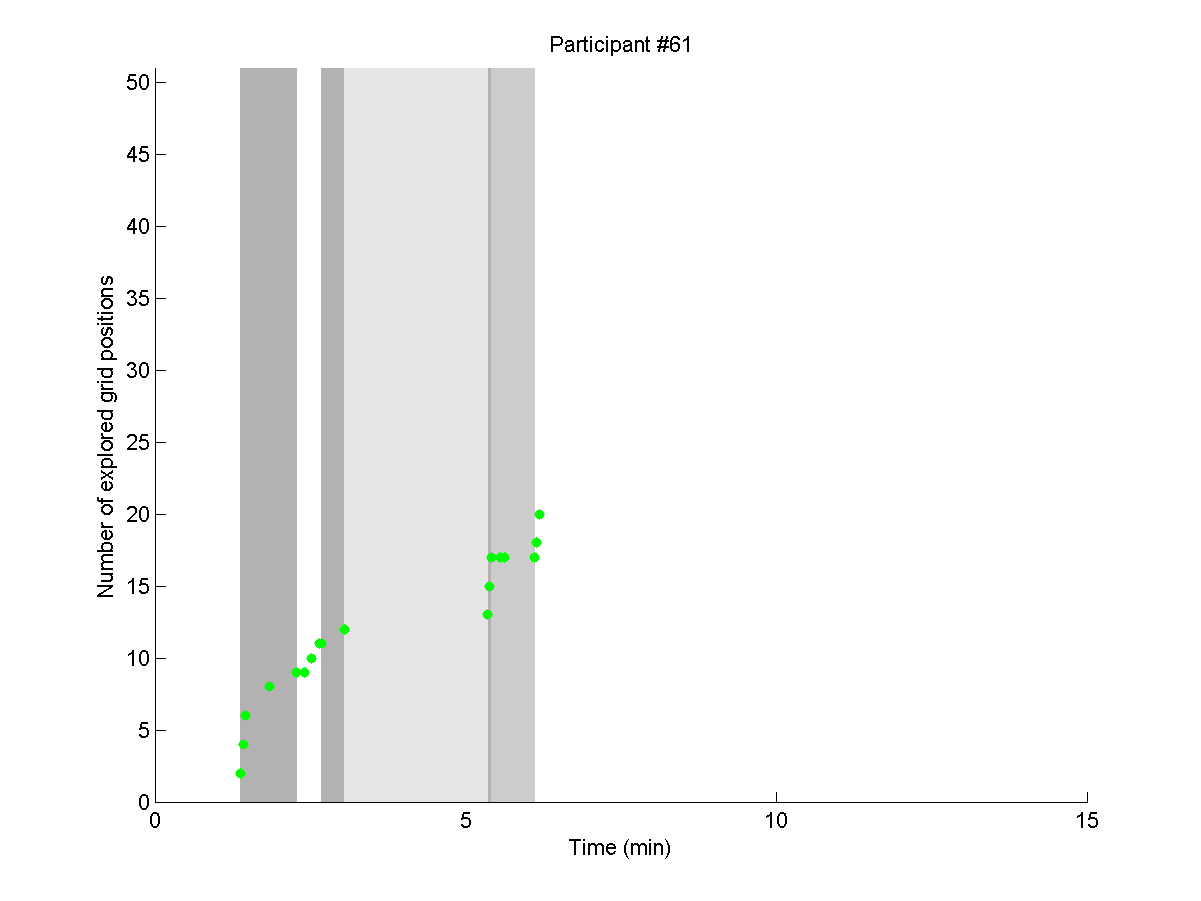

Supplement: Supplementary file 1 [file Presentation1.ZIP › individual plots/61.png]

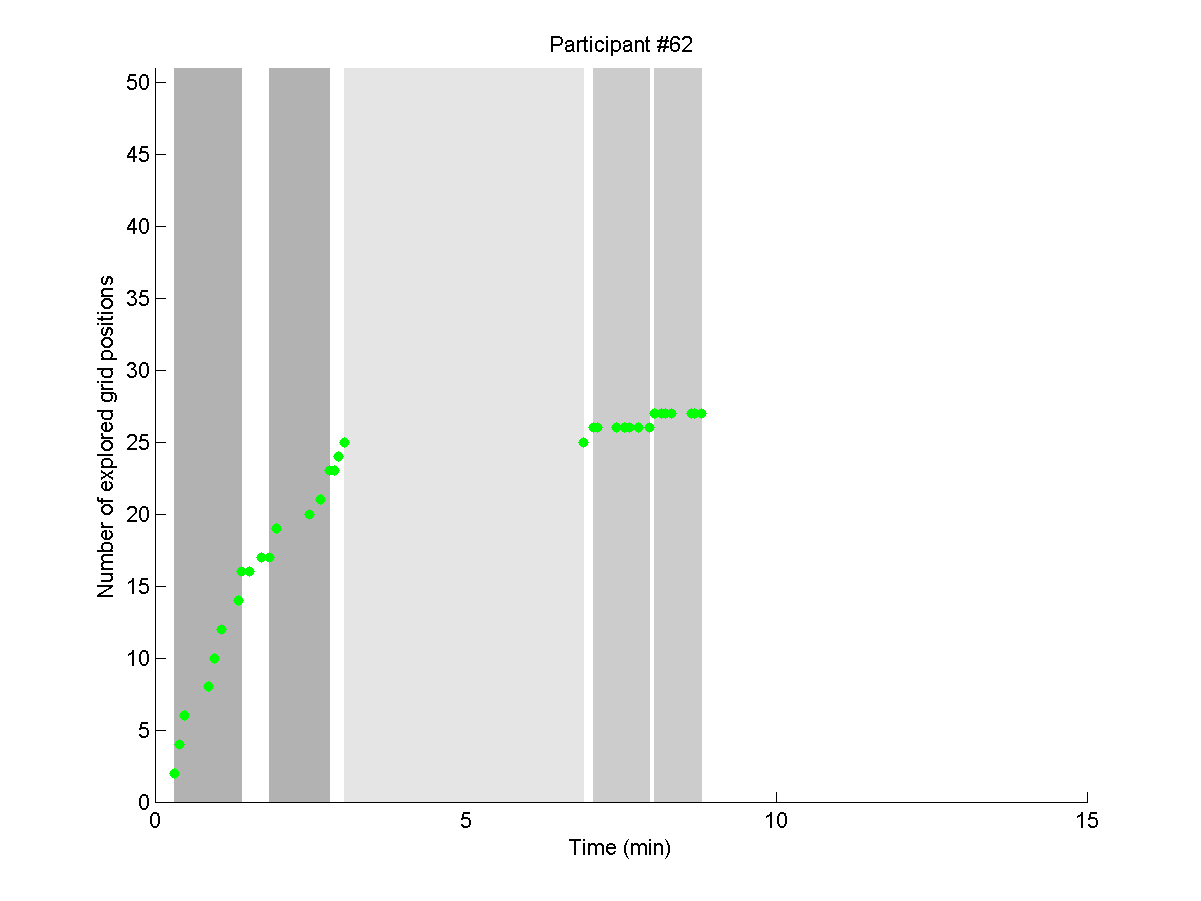

Supplement: Supplementary file 1 [file Presentation1.ZIP › individual plots/62.png]

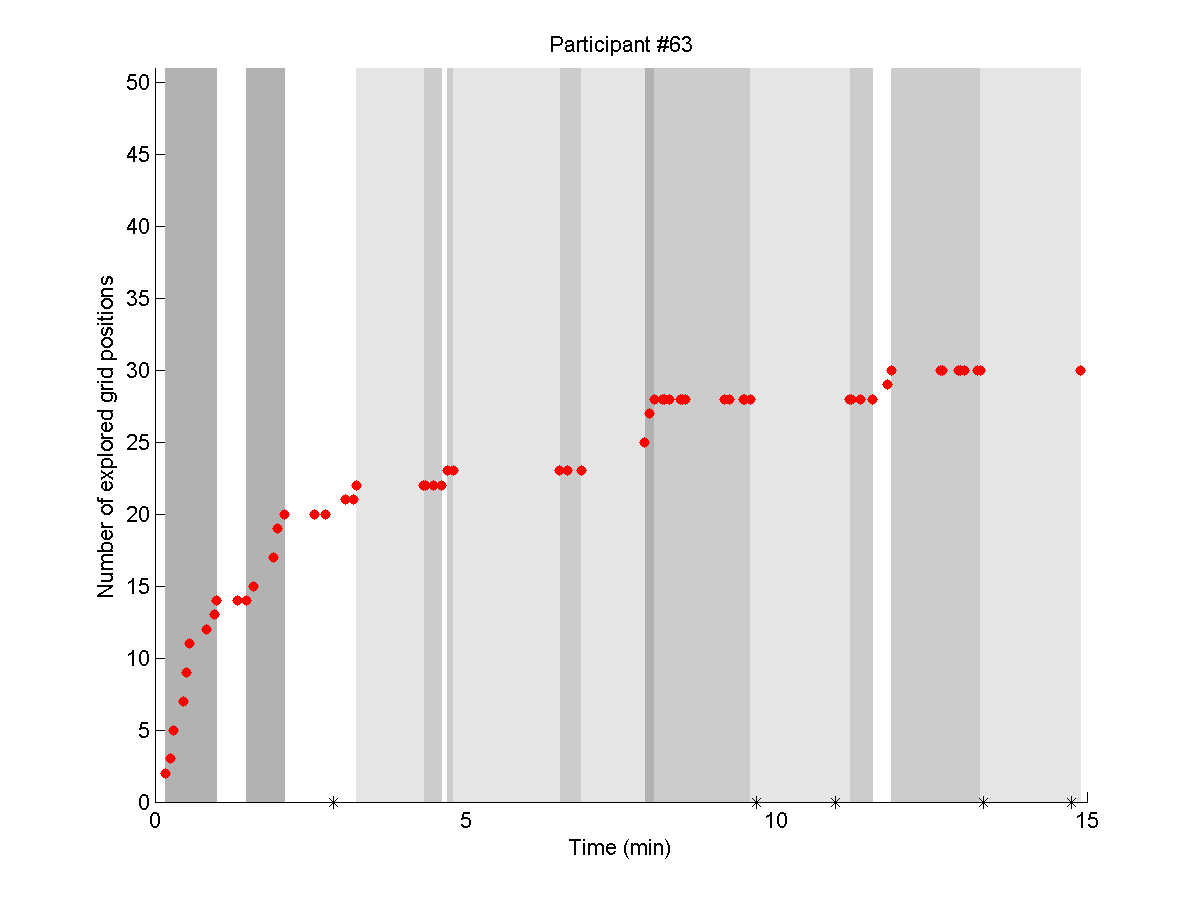

Supplement: Supplementary file 1 [file Presentation1.ZIP › individual plots/63.png]

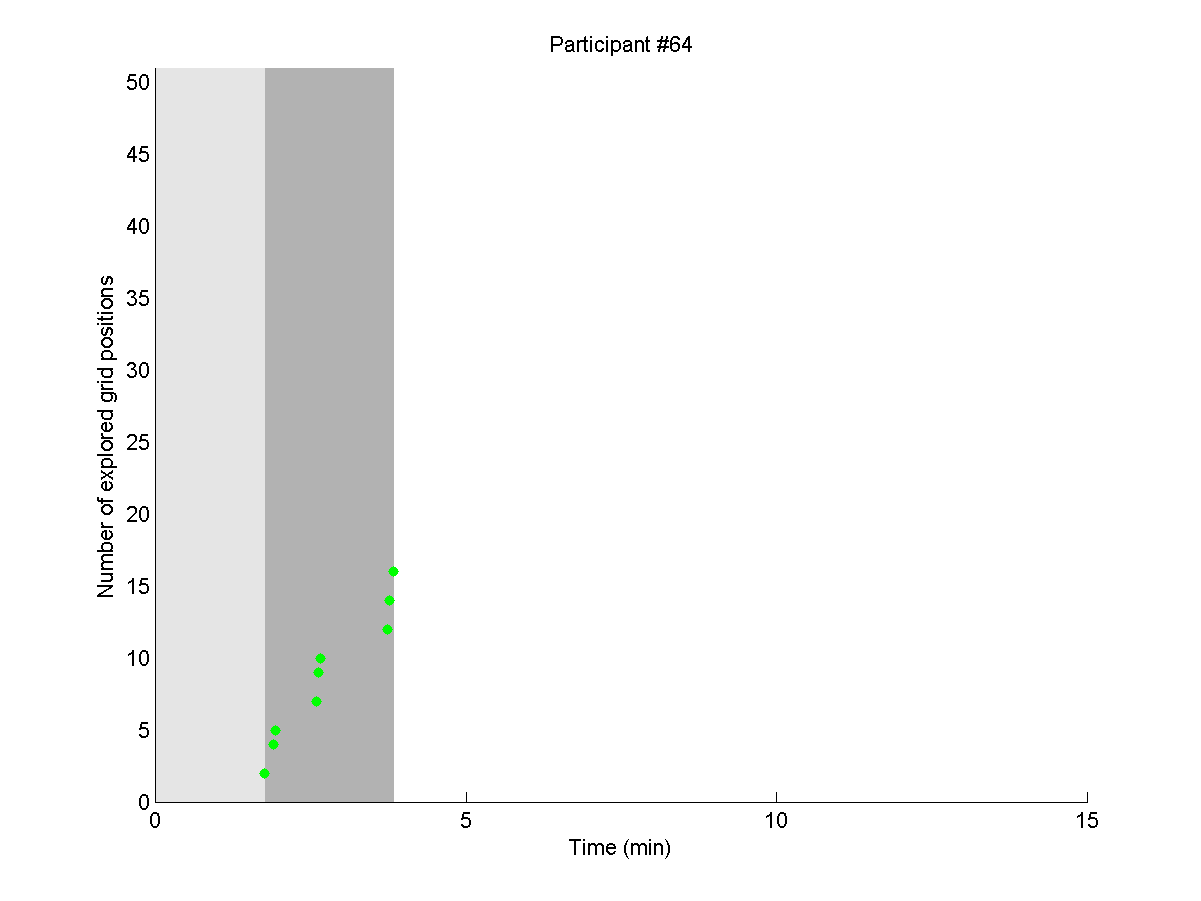

Supplement: Supplementary file 1 [file Presentation1.ZIP › individual plots/64.png]

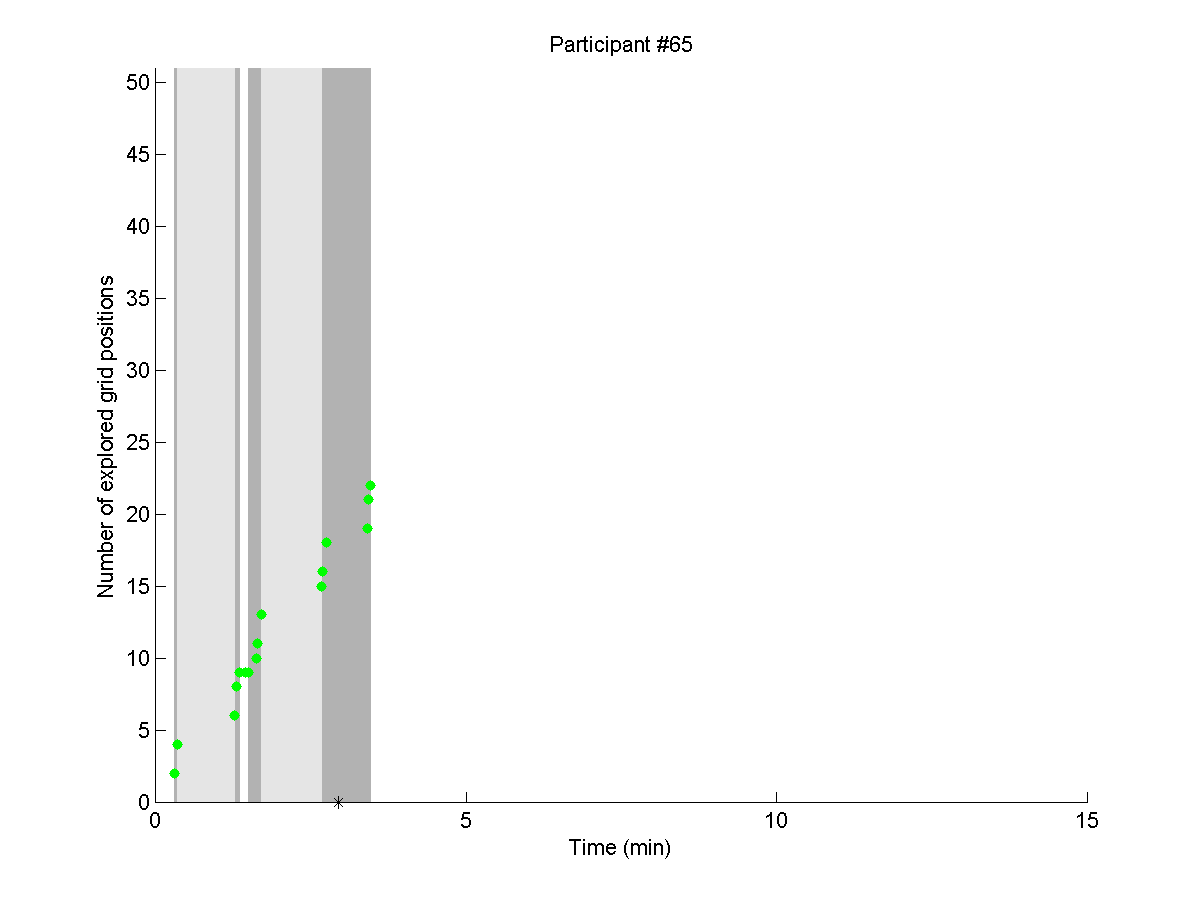

Supplement: Supplementary file 1 [file Presentation1.ZIP › individual plots/65.png]

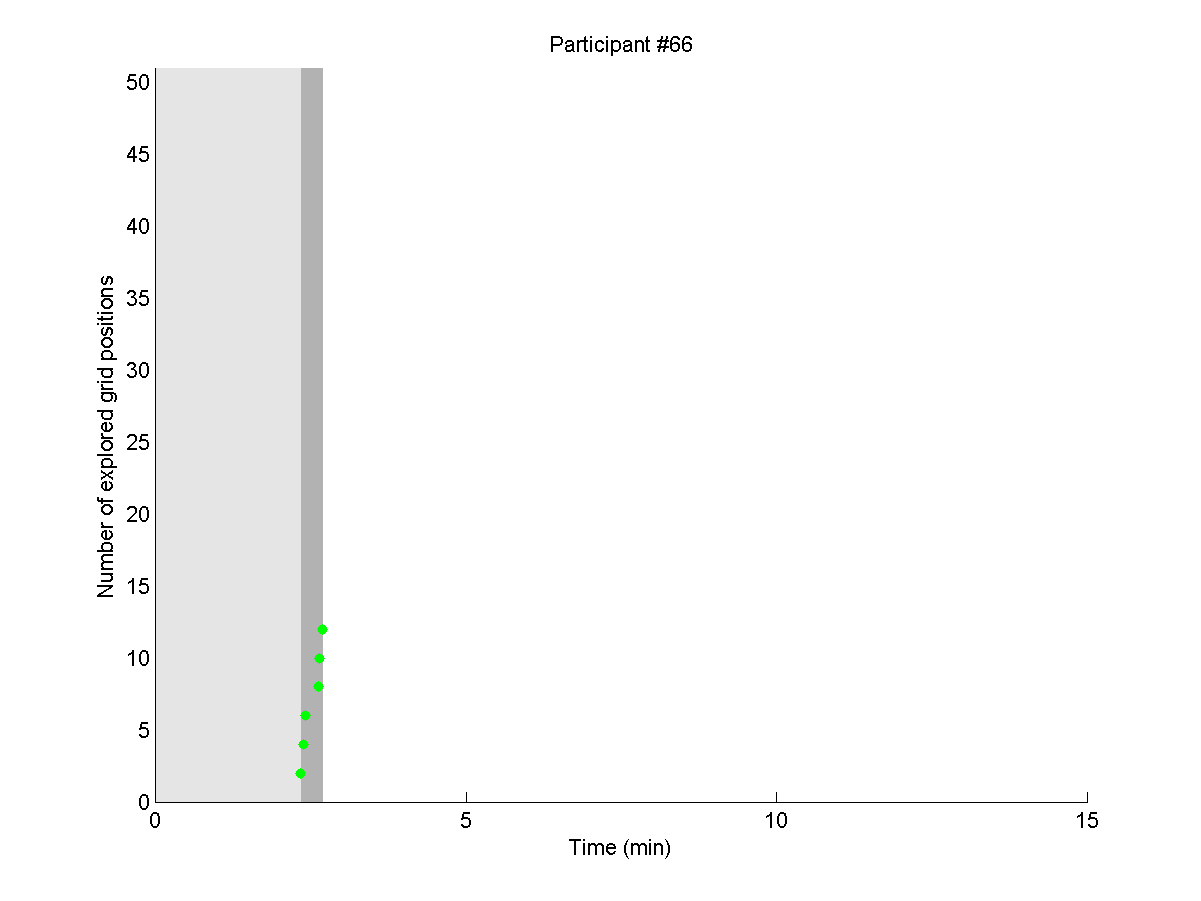

Supplement: Supplementary file 1 [file Presentation1.ZIP › individual plots/66.png]

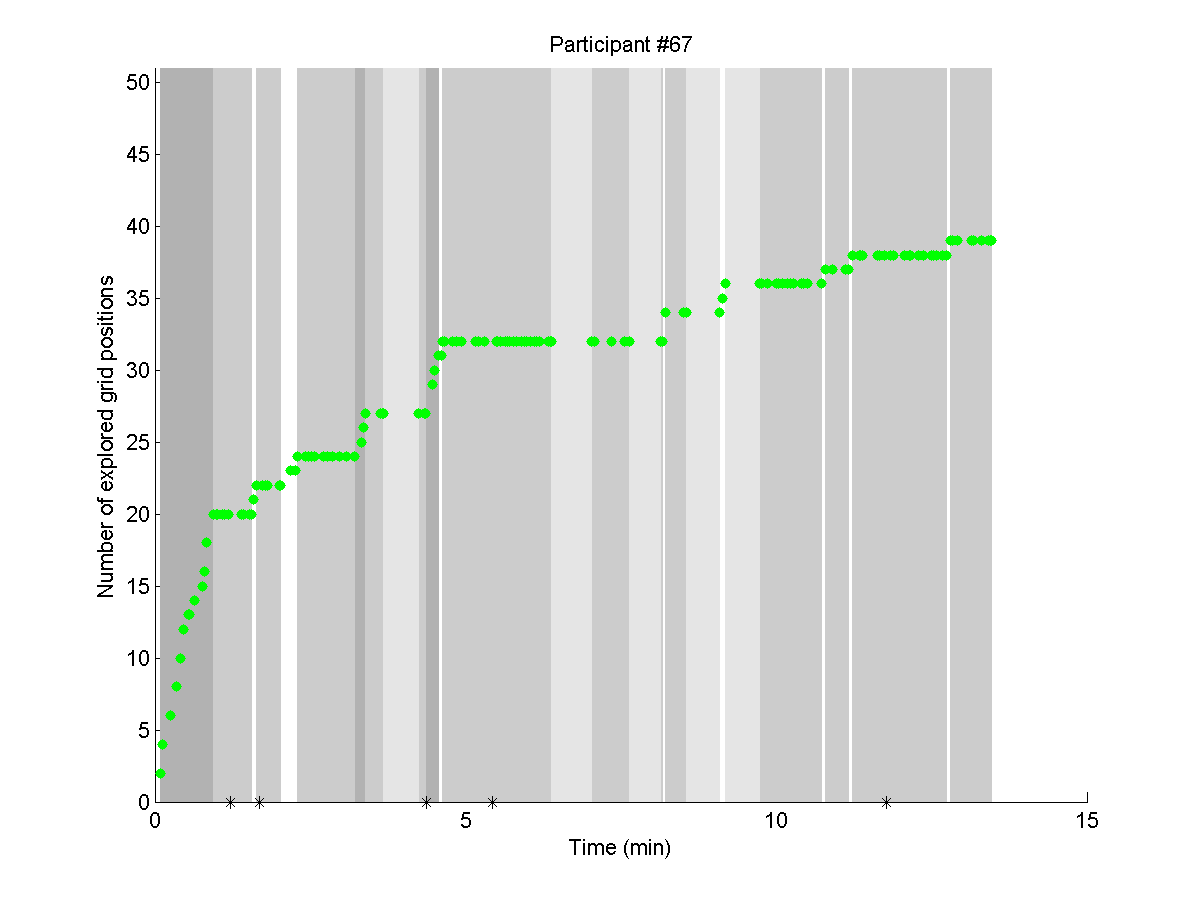

Supplement: Supplementary file 1 [file Presentation1.ZIP › individual plots/67.png]

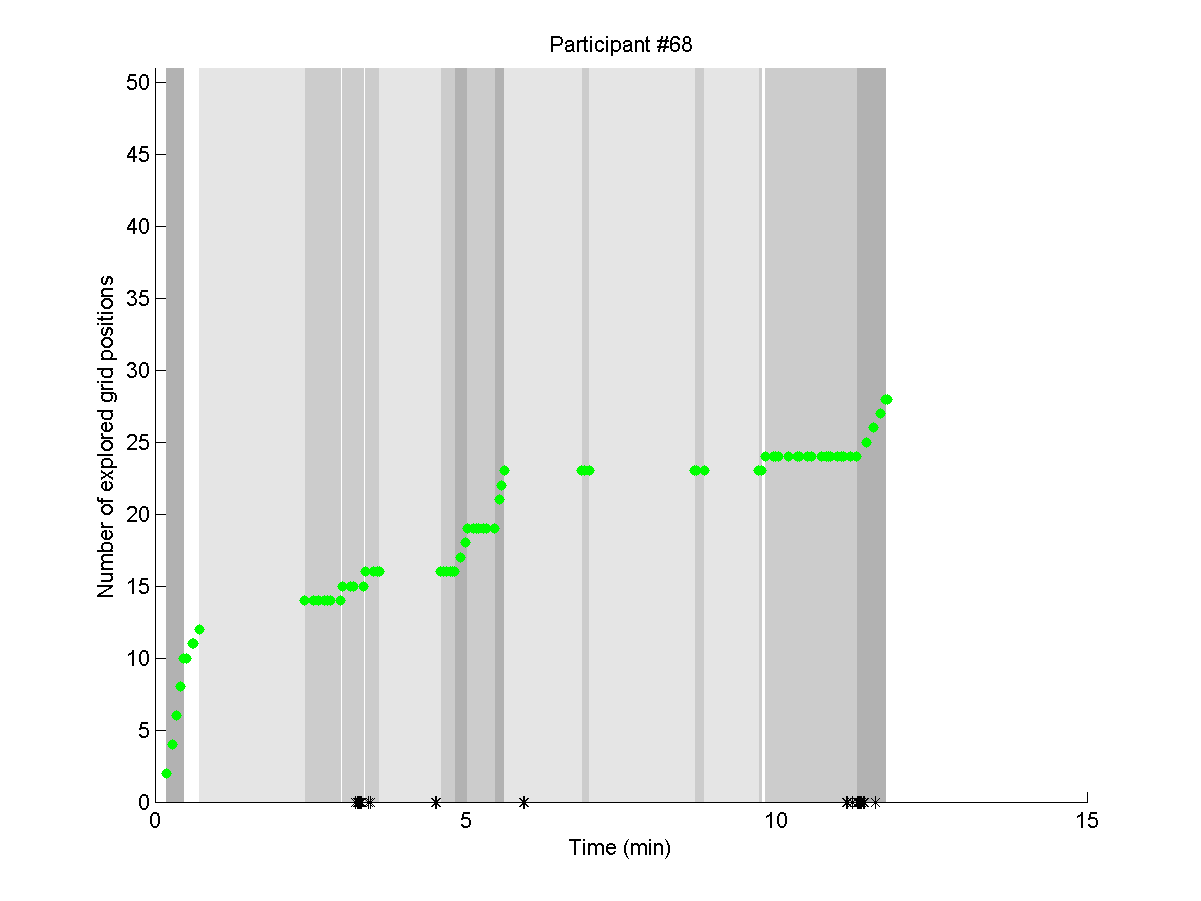

Supplement: Supplementary file 1 [file Presentation1.ZIP › individual plots/68.png]

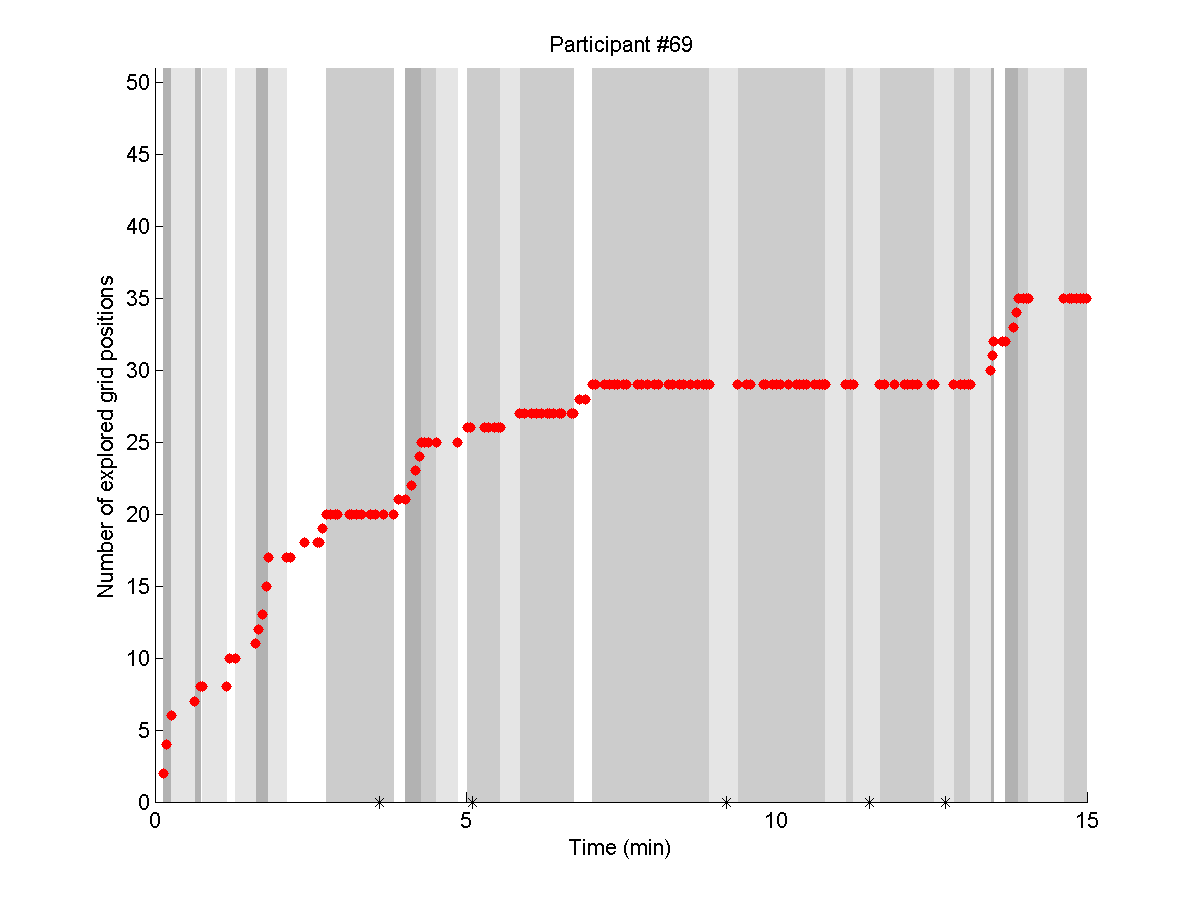

Supplement: Supplementary file 1 [file Presentation1.ZIP › individual plots/69.png]

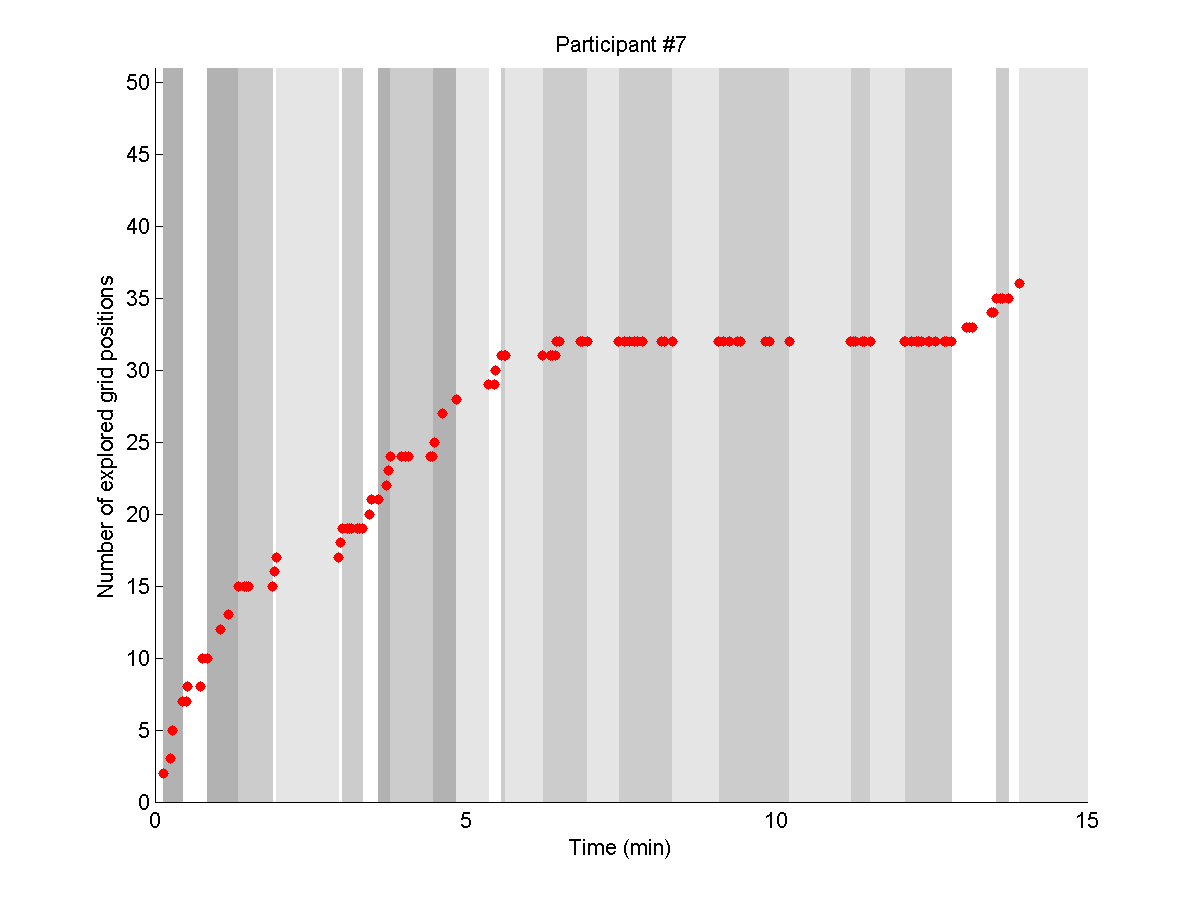

Supplement: Supplementary file 1 [file Presentation1.ZIP › individual plots/7.png]

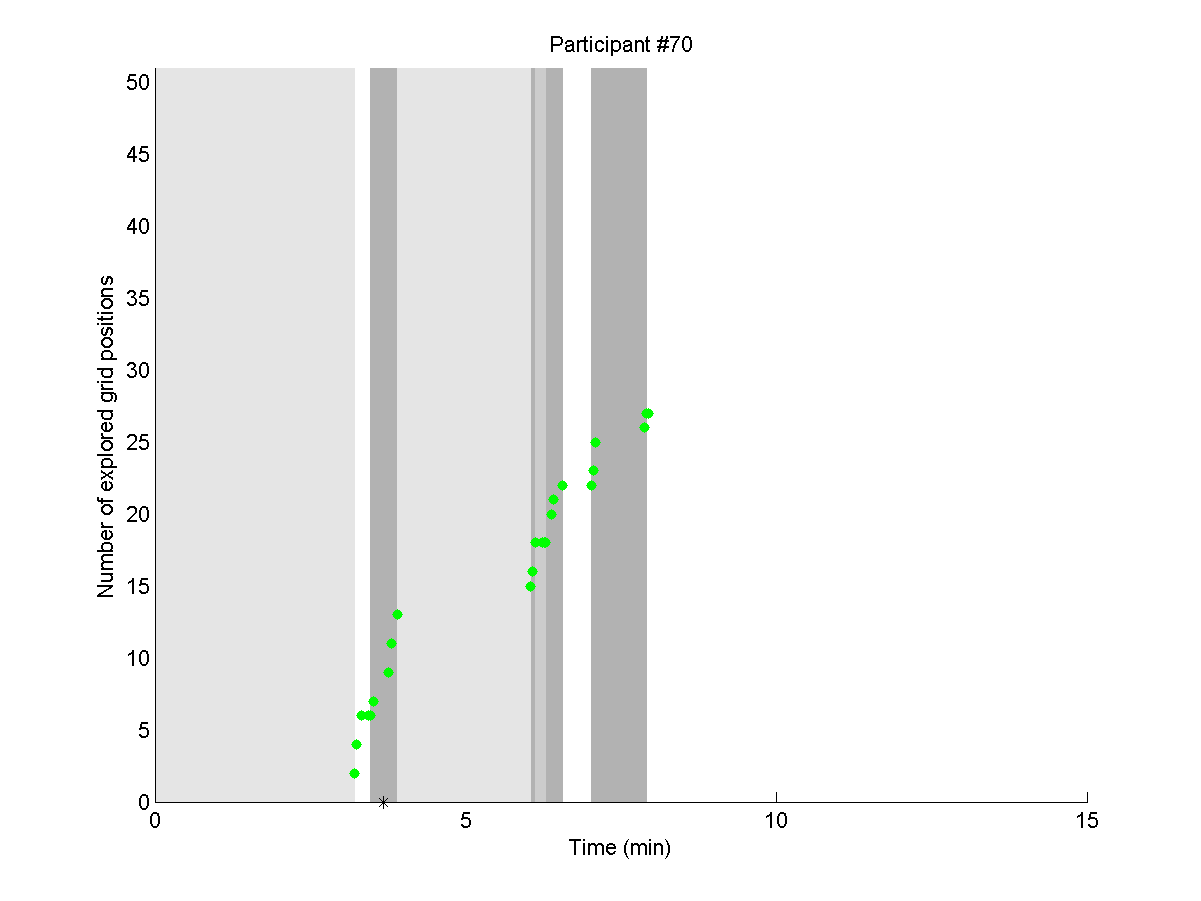

Supplement: Supplementary file 1 [file Presentation1.ZIP › individual plots/70.png]

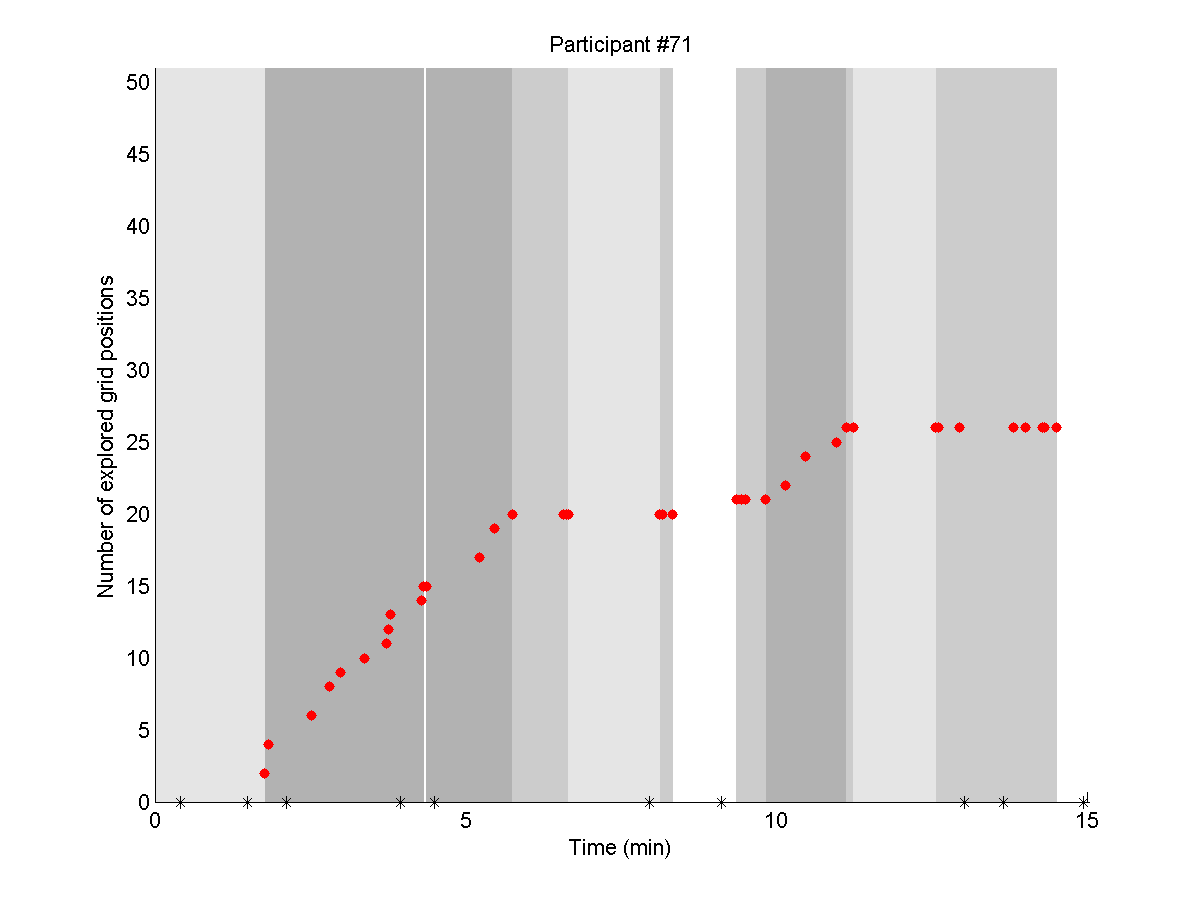

Supplement: Supplementary file 1 [file Presentation1.ZIP › individual plots/71.png]

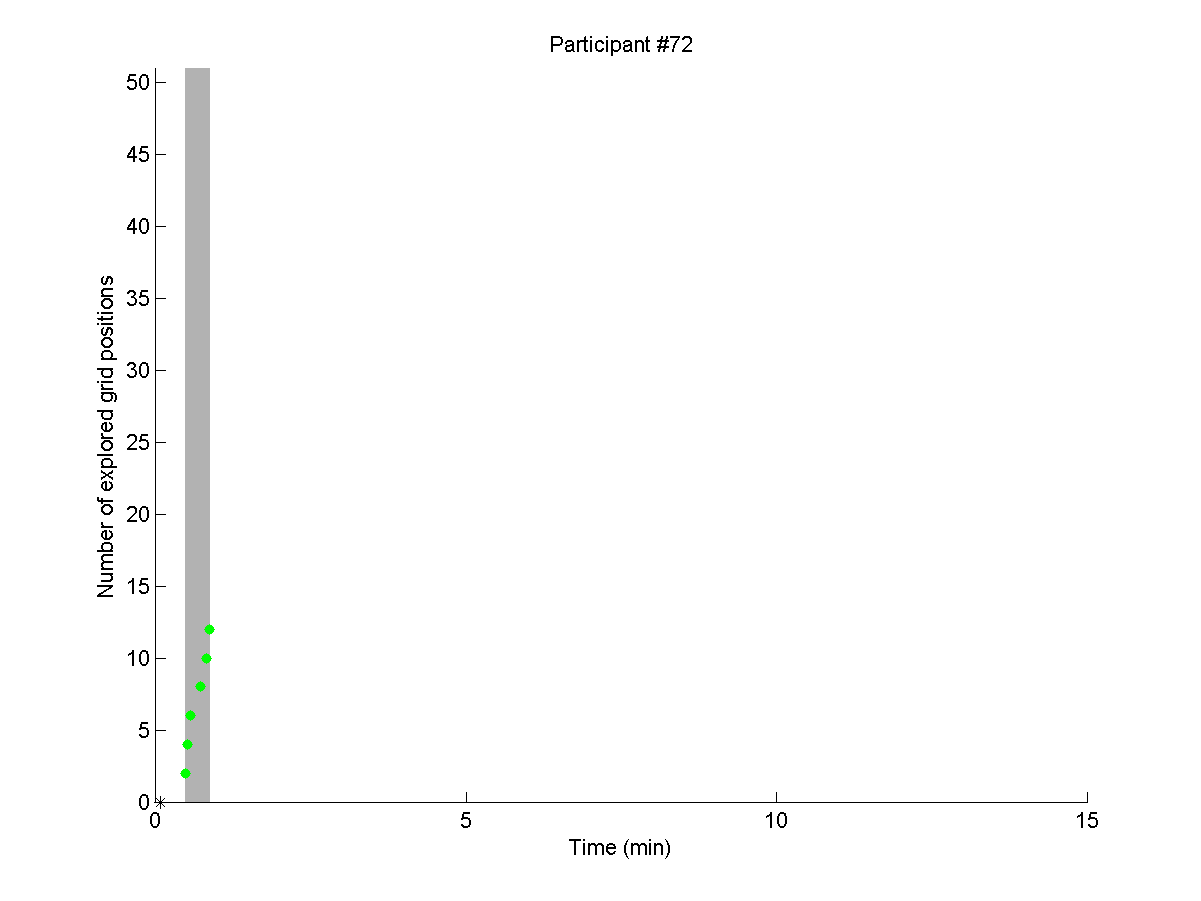

Supplement: Supplementary file 1 [file Presentation1.ZIP › individual plots/72.png]

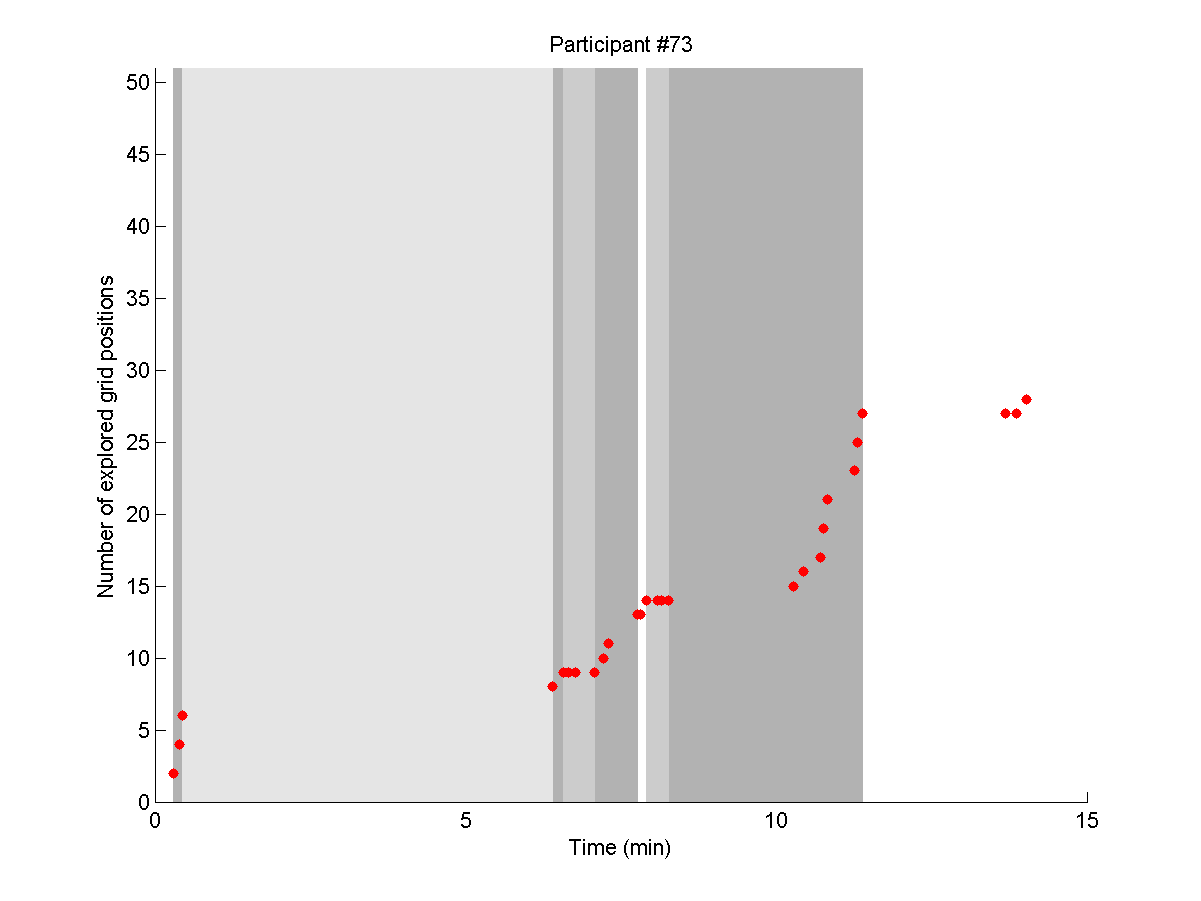

Supplement: Supplementary file 1 [file Presentation1.ZIP › individual plots/73.png]

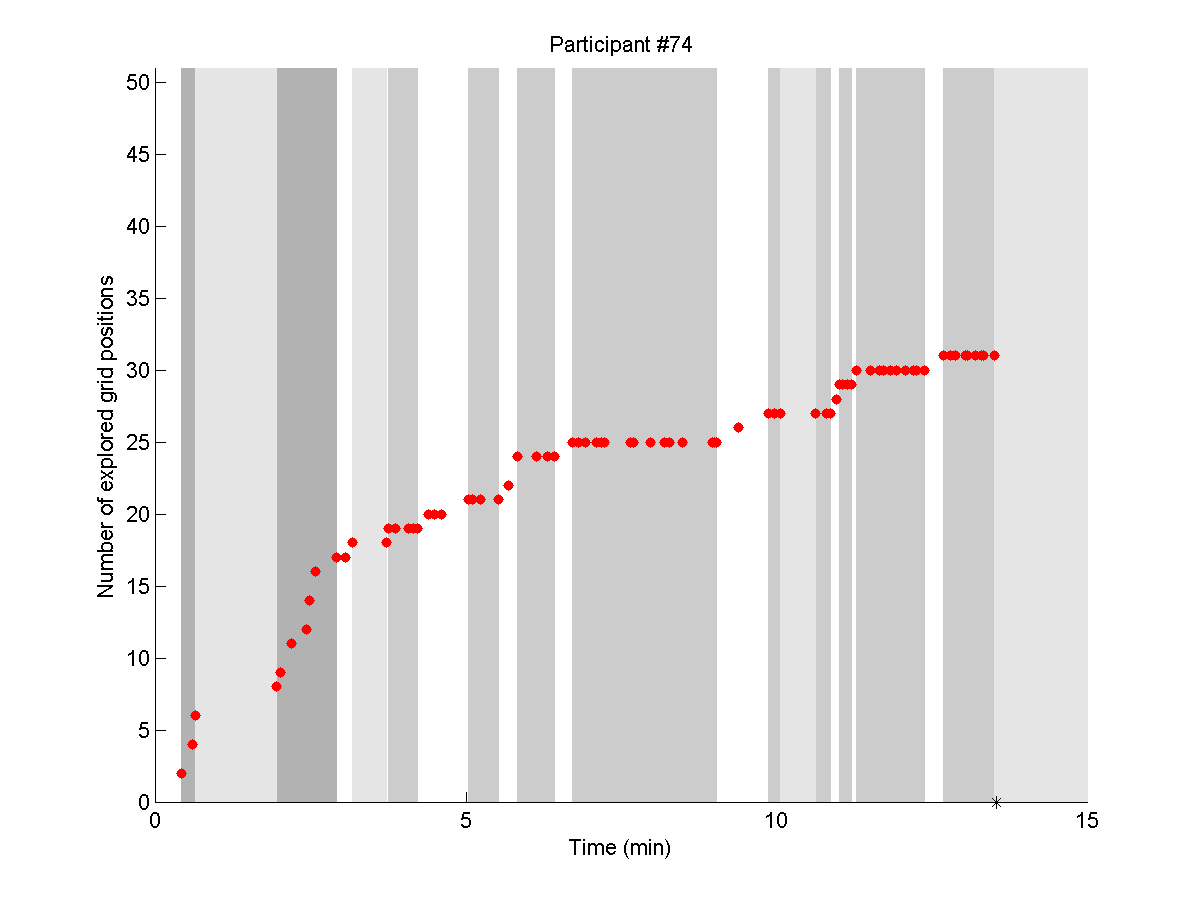

Supplement: Supplementary file 1 [file Presentation1.ZIP › individual plots/74.png]

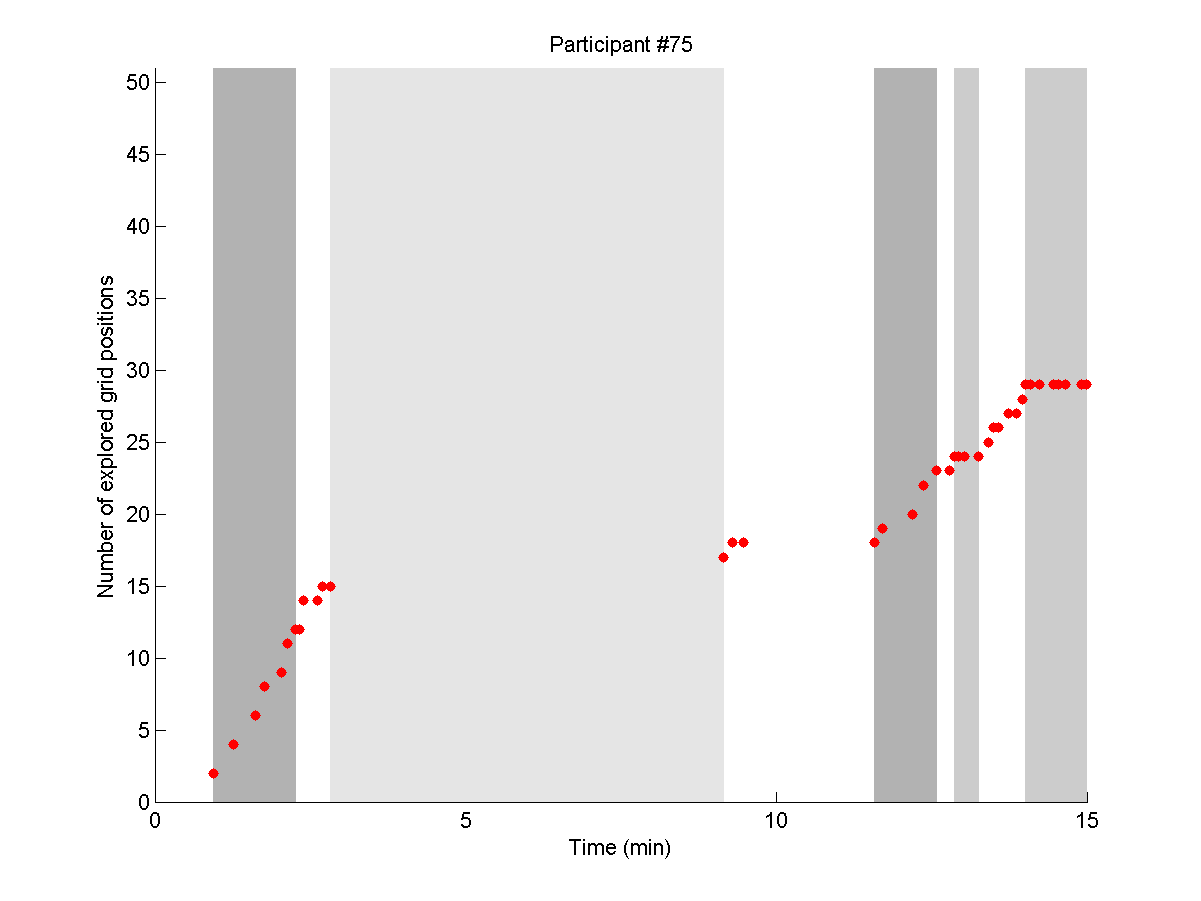

Supplement: Supplementary file 1 [file Presentation1.ZIP › individual plots/75.png]

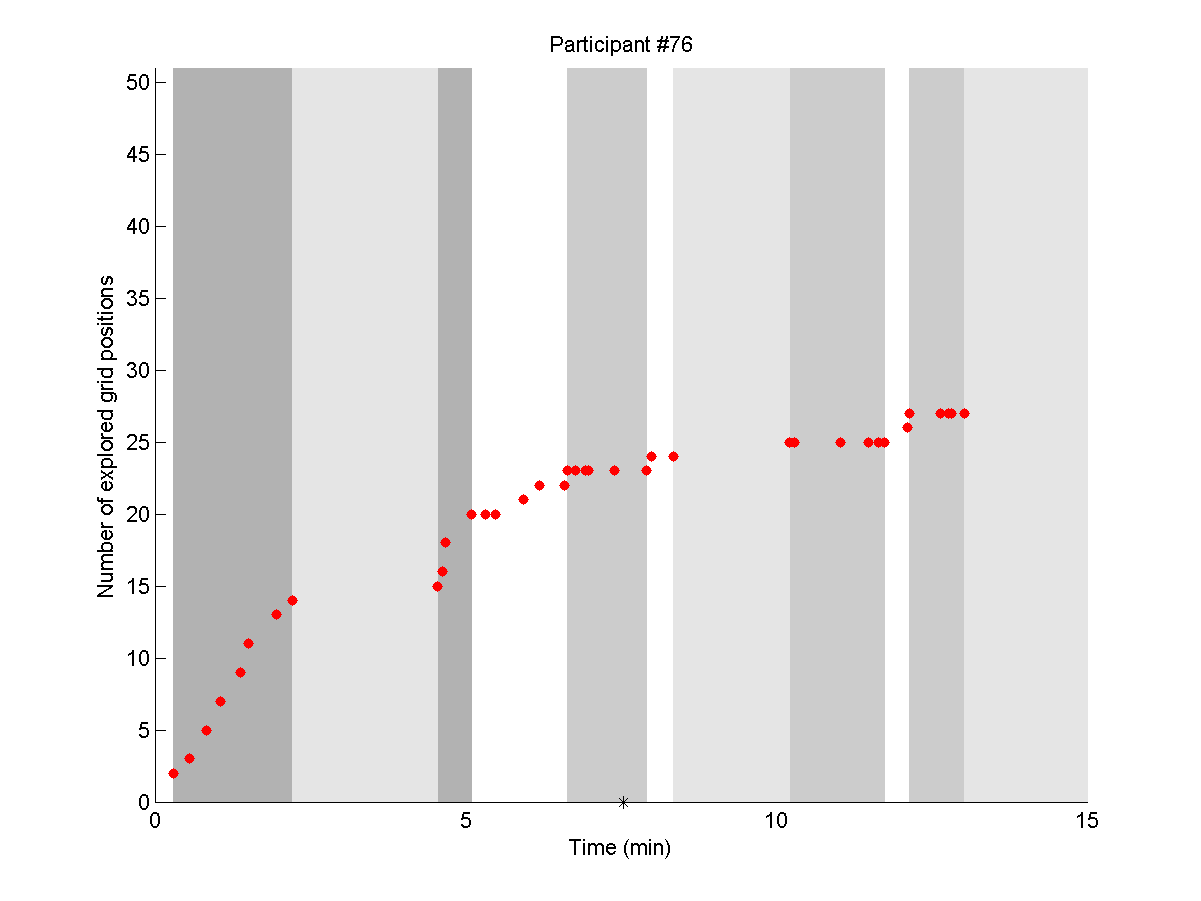

Supplement: Supplementary file 1 [file Presentation1.ZIP › individual plots/76.png]

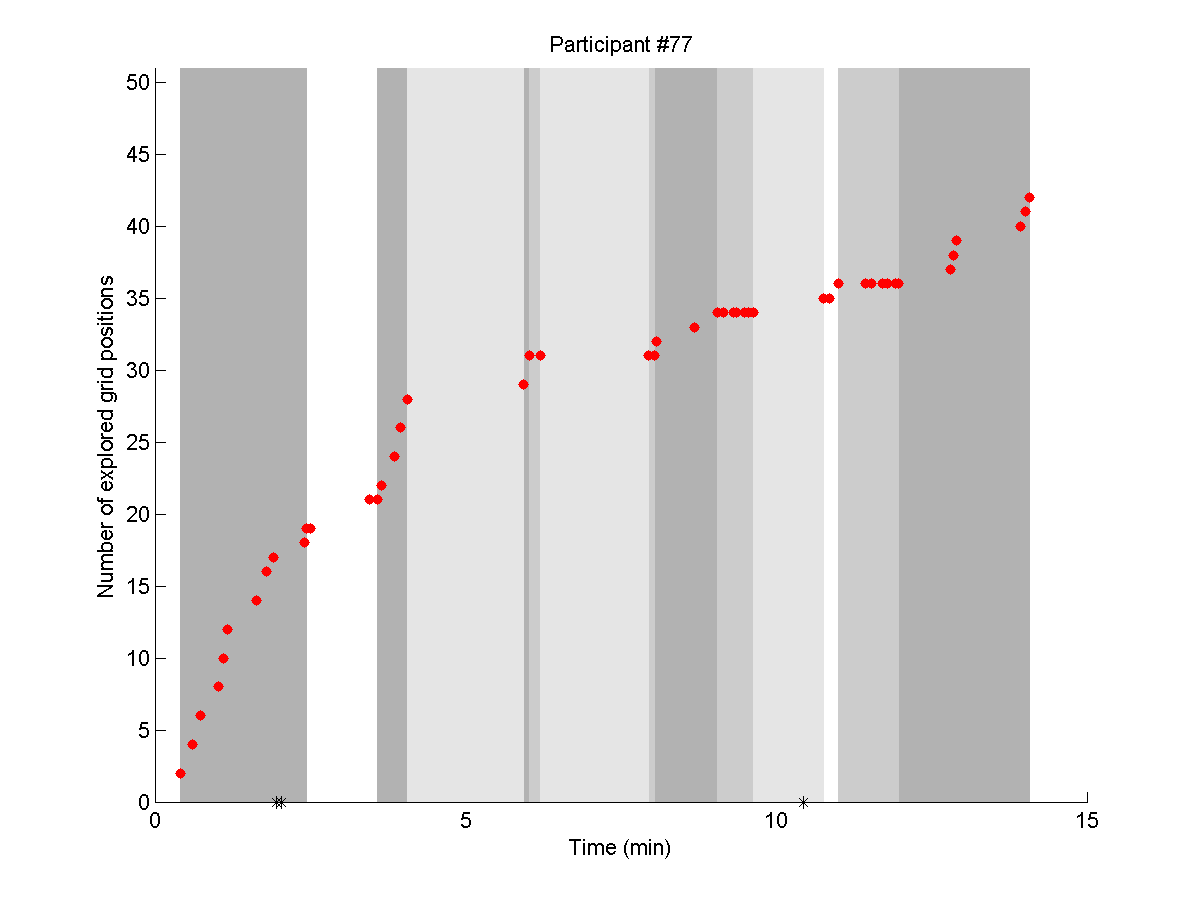

Supplement: Supplementary file 1 [file Presentation1.ZIP › individual plots/77.png]

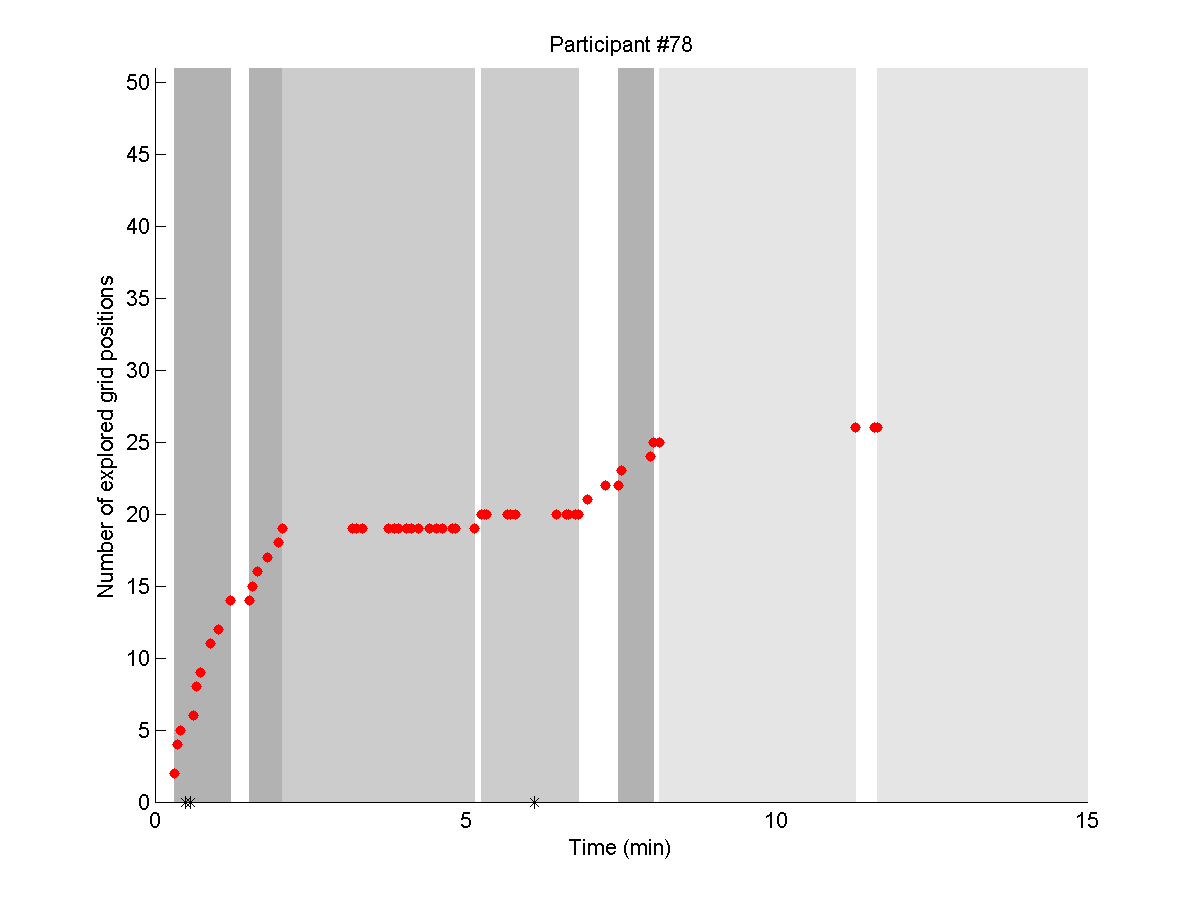

Supplement: Supplementary file 1 [file Presentation1.ZIP › individual plots/78.png]

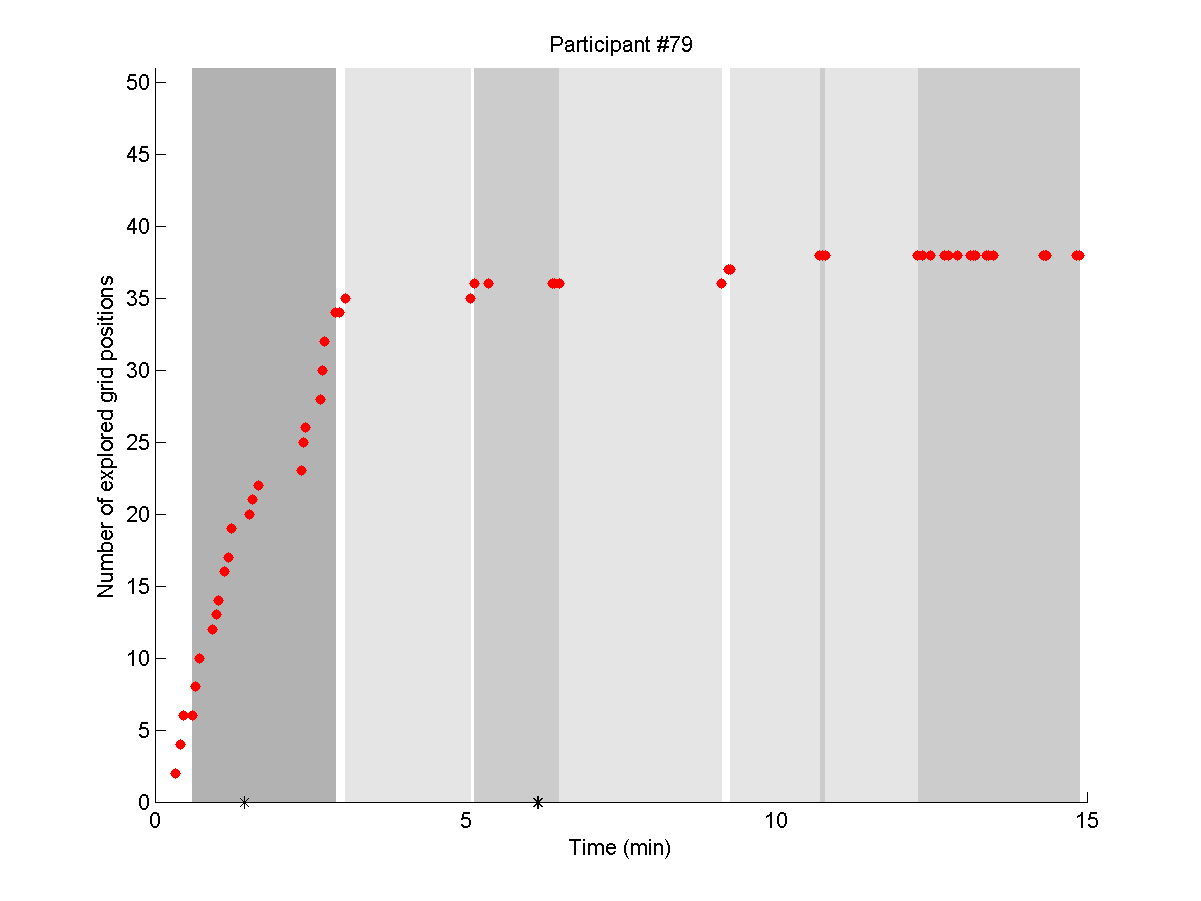

Supplement: Supplementary file 1 [file Presentation1.ZIP › individual plots/79.png]

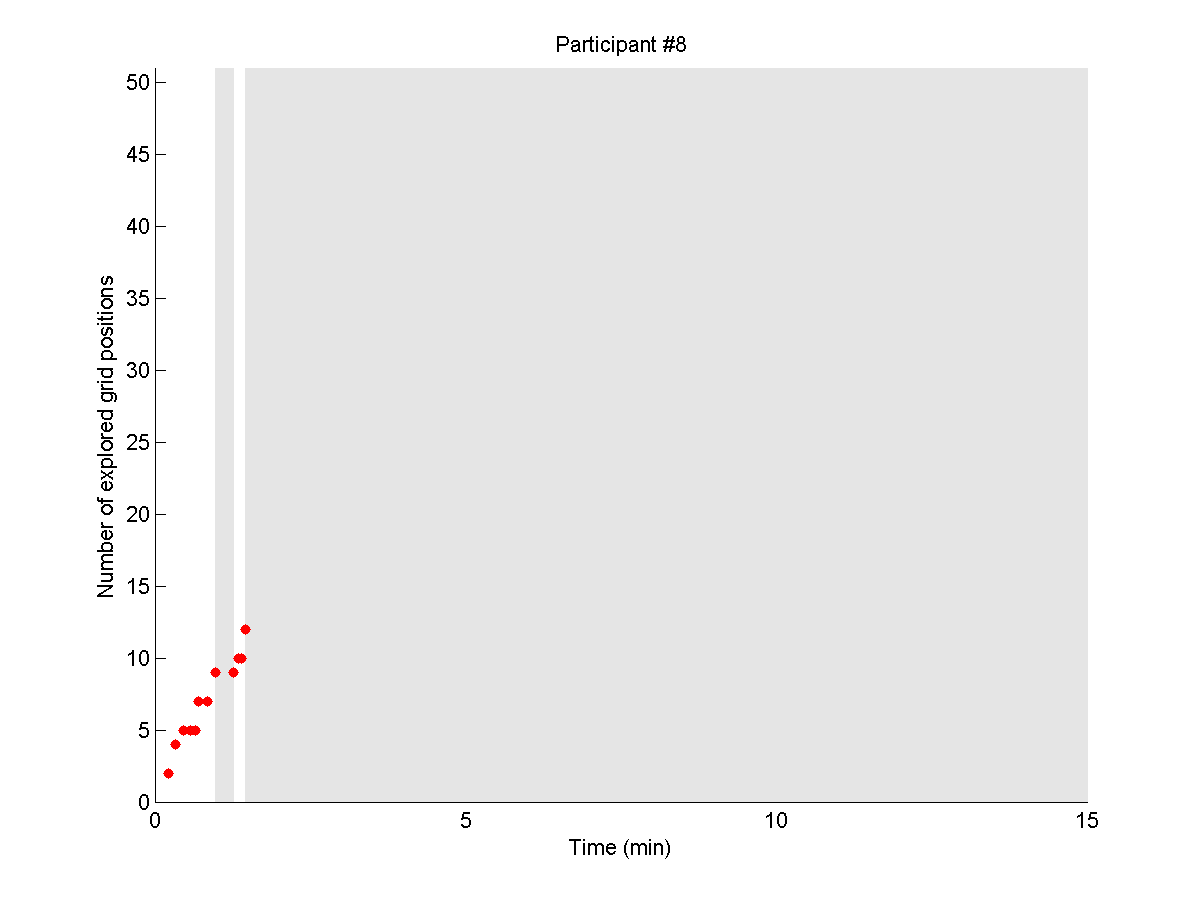

Supplement: Supplementary file 1 [file Presentation1.ZIP › individual plots/8.png]

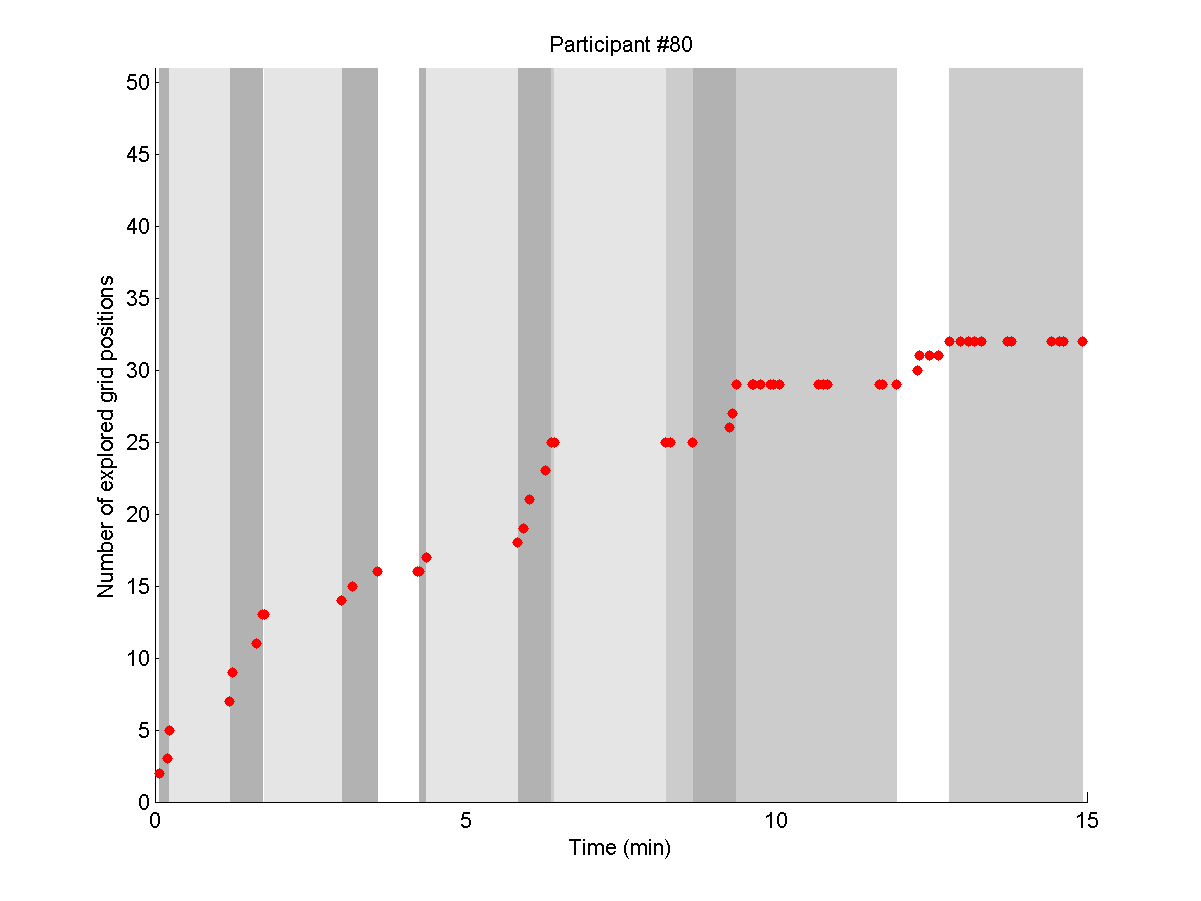

Supplement: Supplementary file 1 [file Presentation1.ZIP › individual plots/80.png]

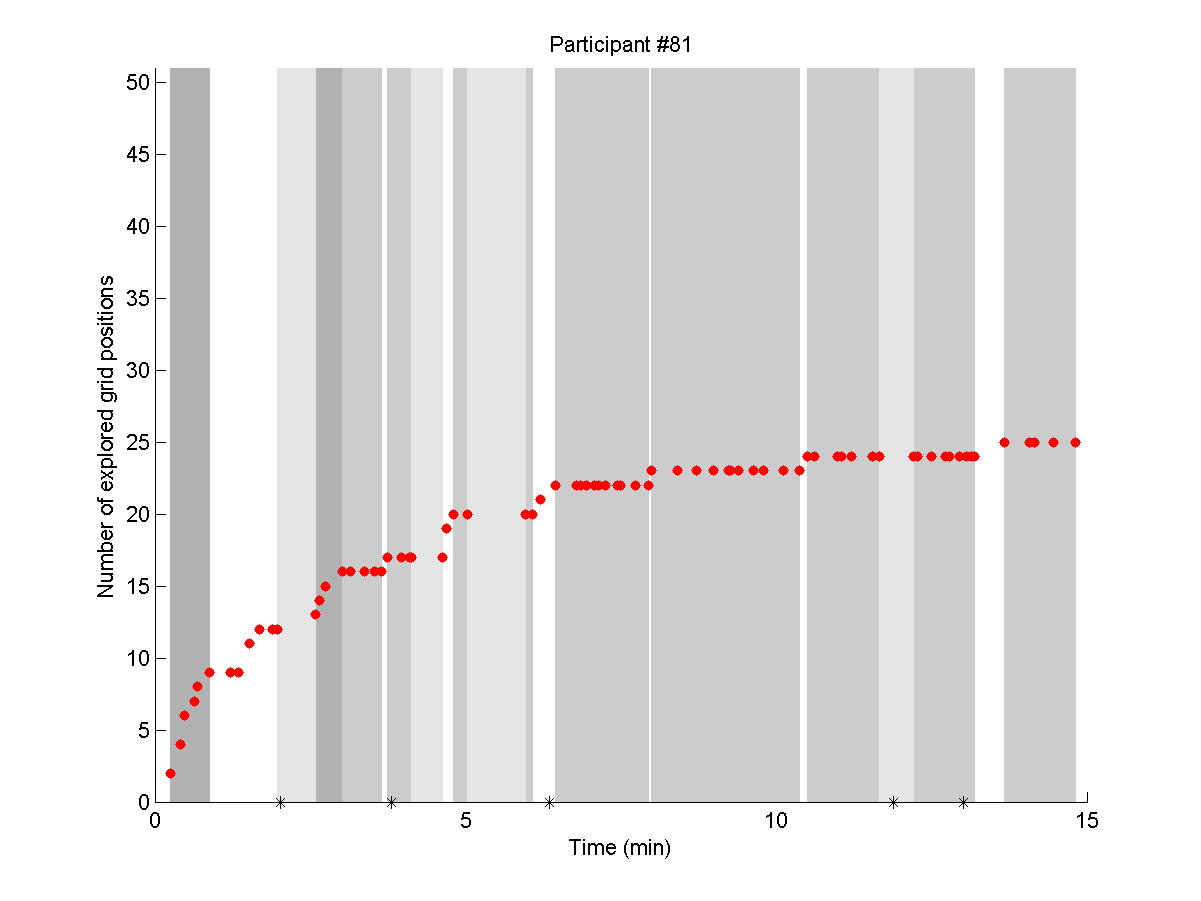

Supplement: Supplementary file 1 [file Presentation1.ZIP › individual plots/81.png]

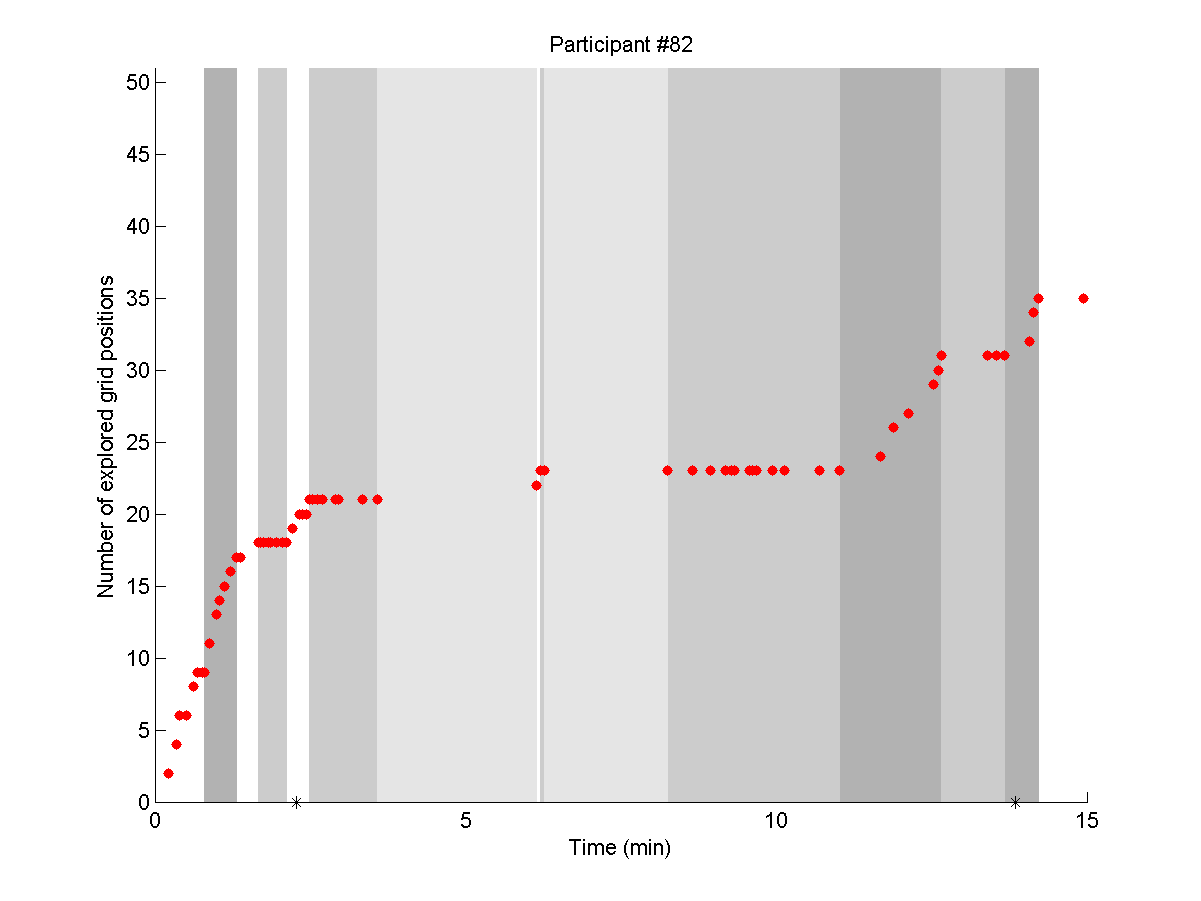

Supplement: Supplementary file 1 [file Presentation1.ZIP › individual plots/82.png]

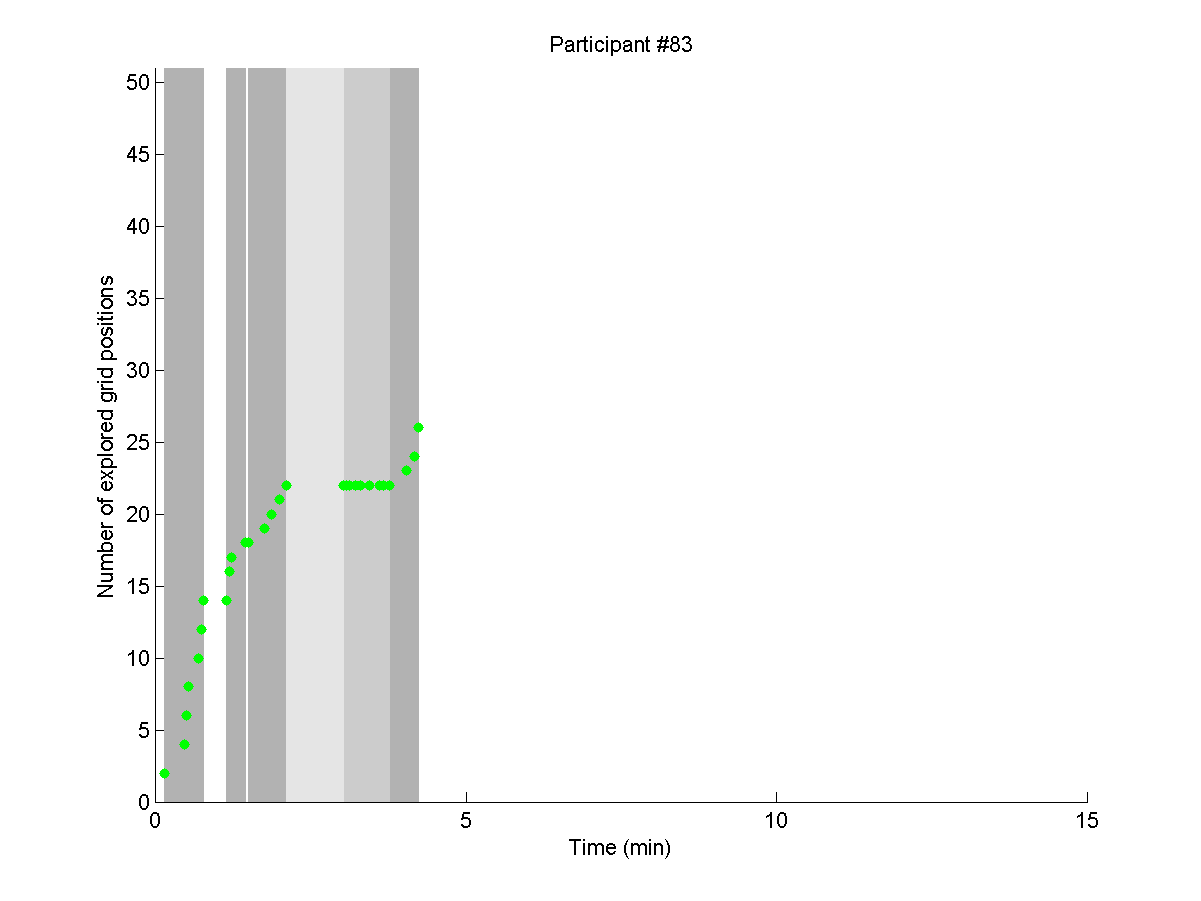

Supplement: Supplementary file 1 [file Presentation1.ZIP › individual plots/83.png]

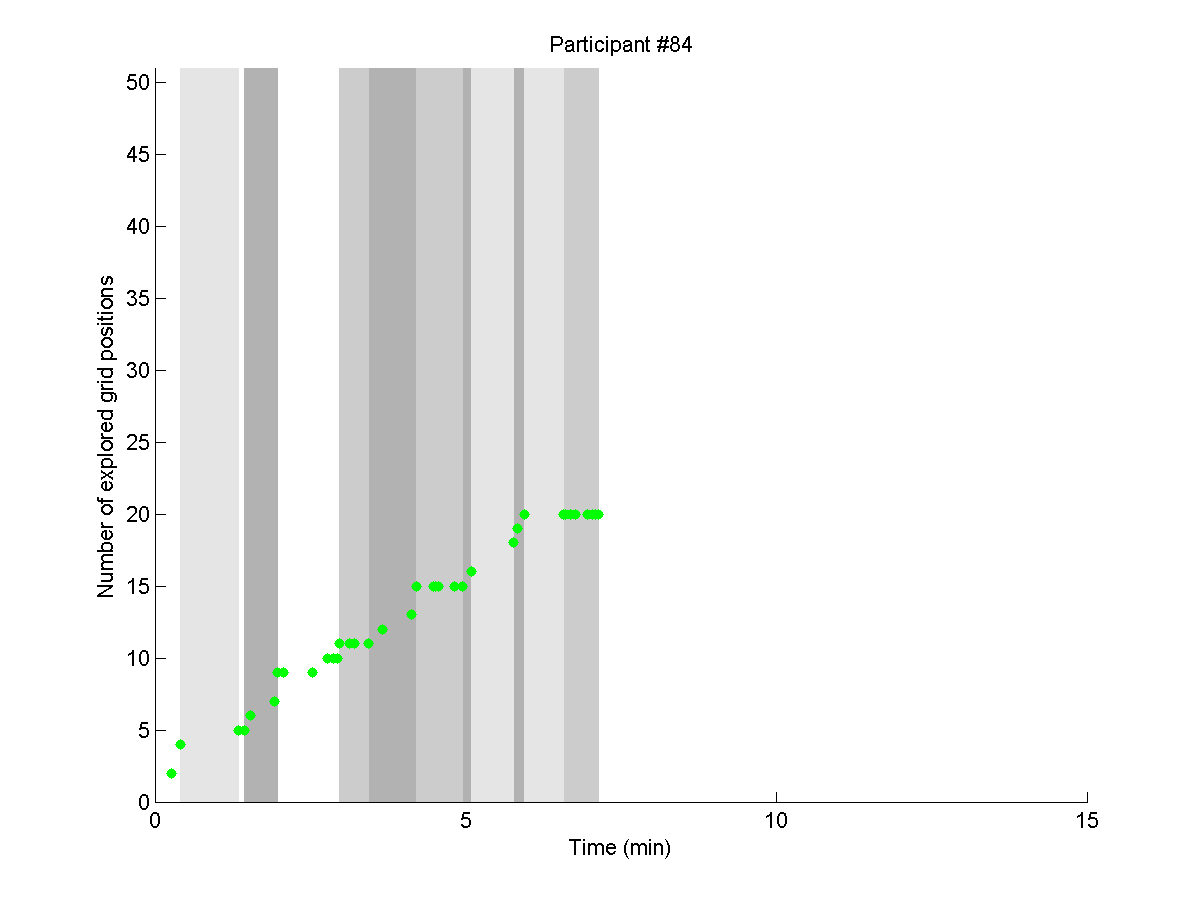

Supplement: Supplementary file 1 [file Presentation1.ZIP › individual plots/84.png]

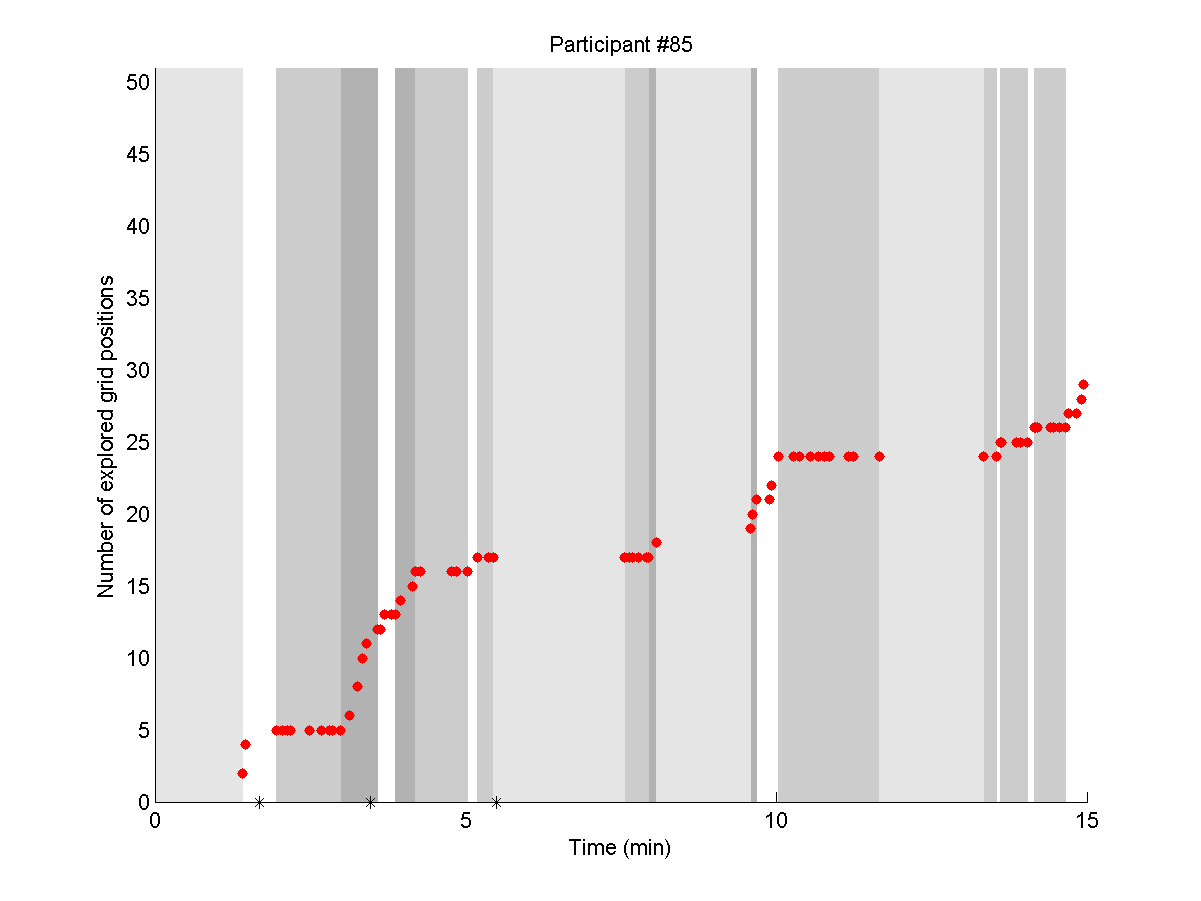

Supplement: Supplementary file 1 [file Presentation1.ZIP › individual plots/85.png]

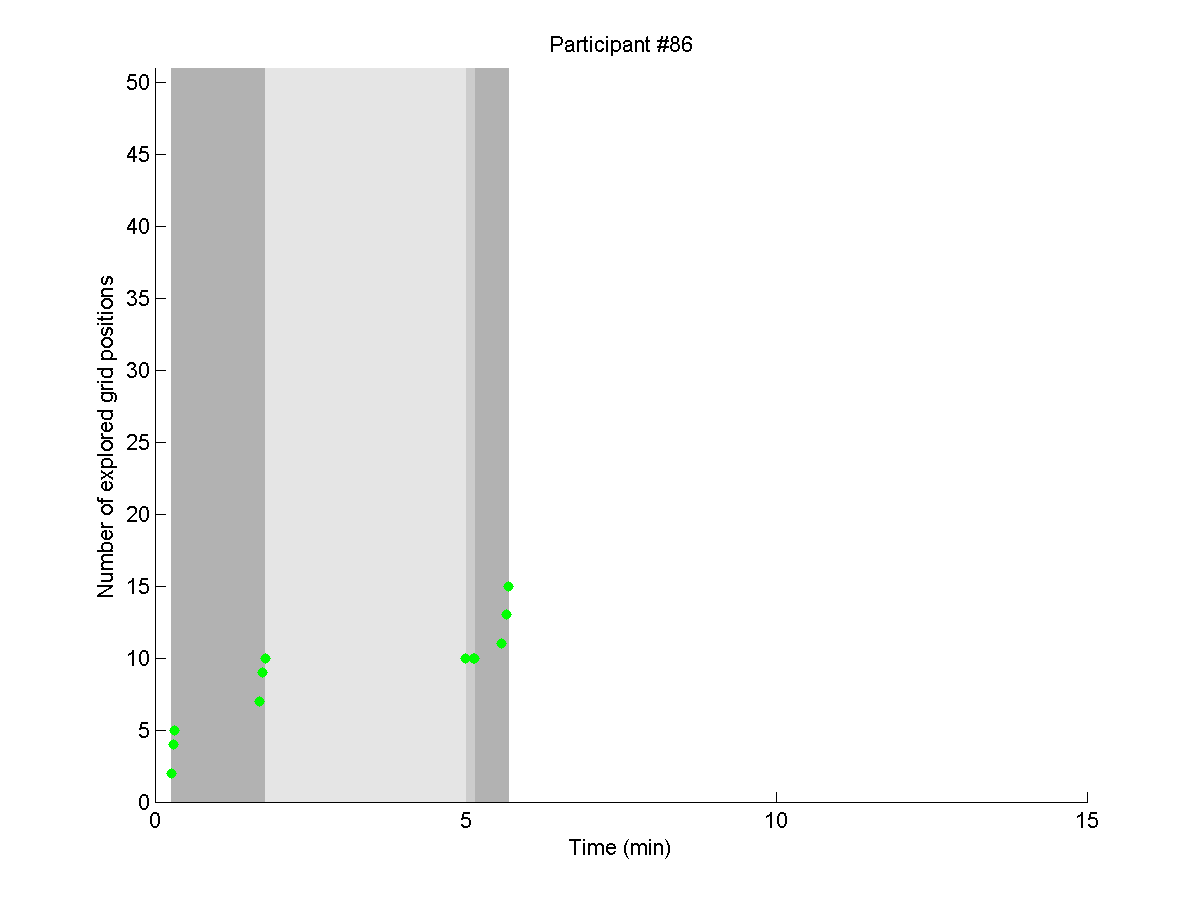

Supplement: Supplementary file 1 [file Presentation1.ZIP › individual plots/86.png]

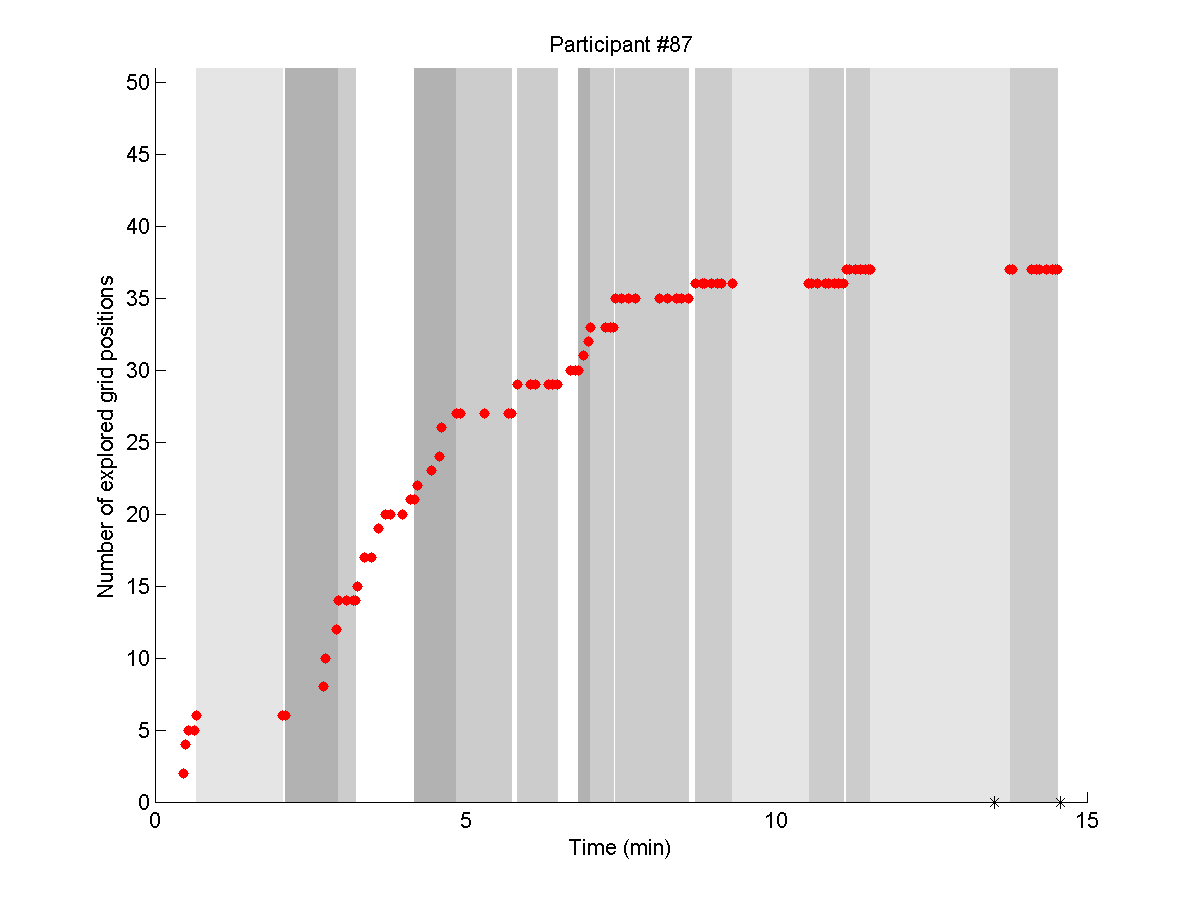

Supplement: Supplementary file 1 [file Presentation1.ZIP › individual plots/87.png]

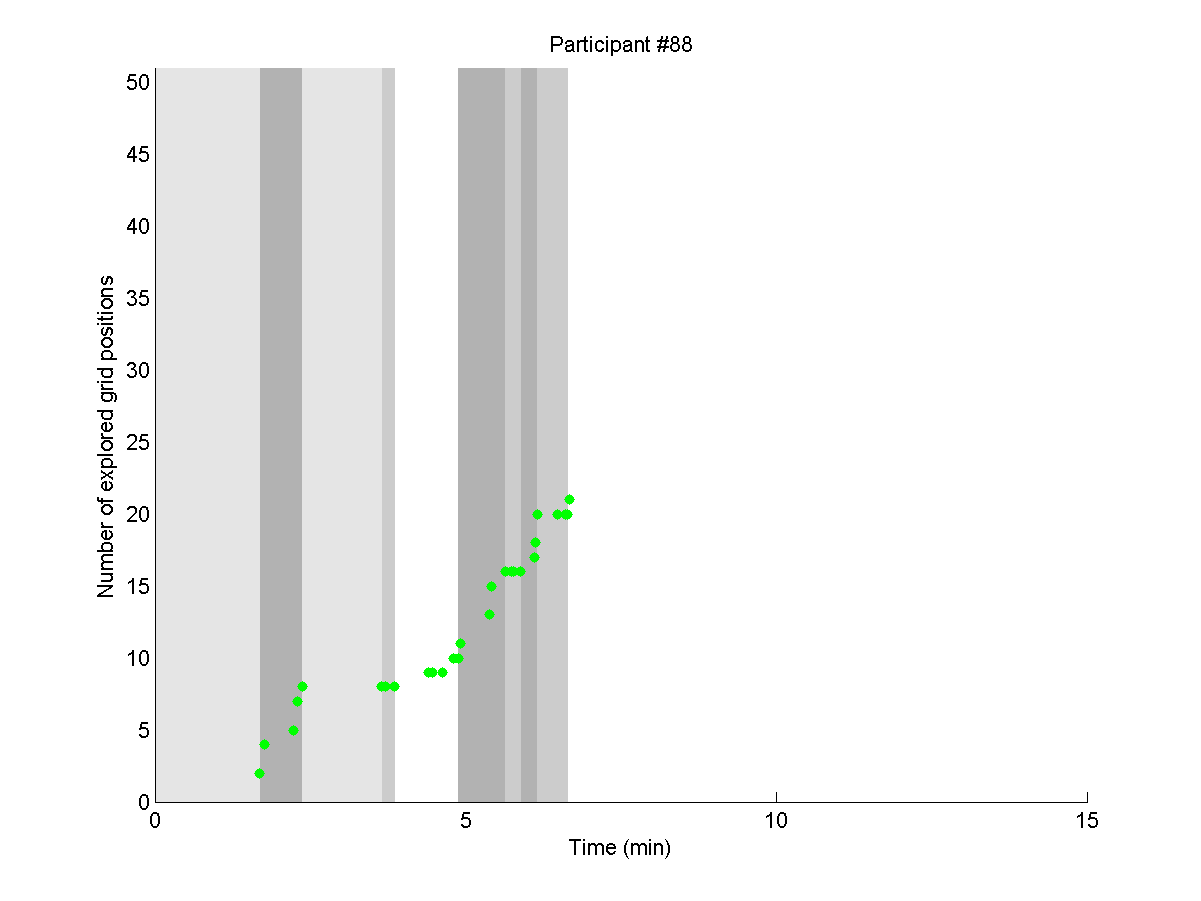

Supplement: Supplementary file 1 [file Presentation1.ZIP › individual plots/88.png]

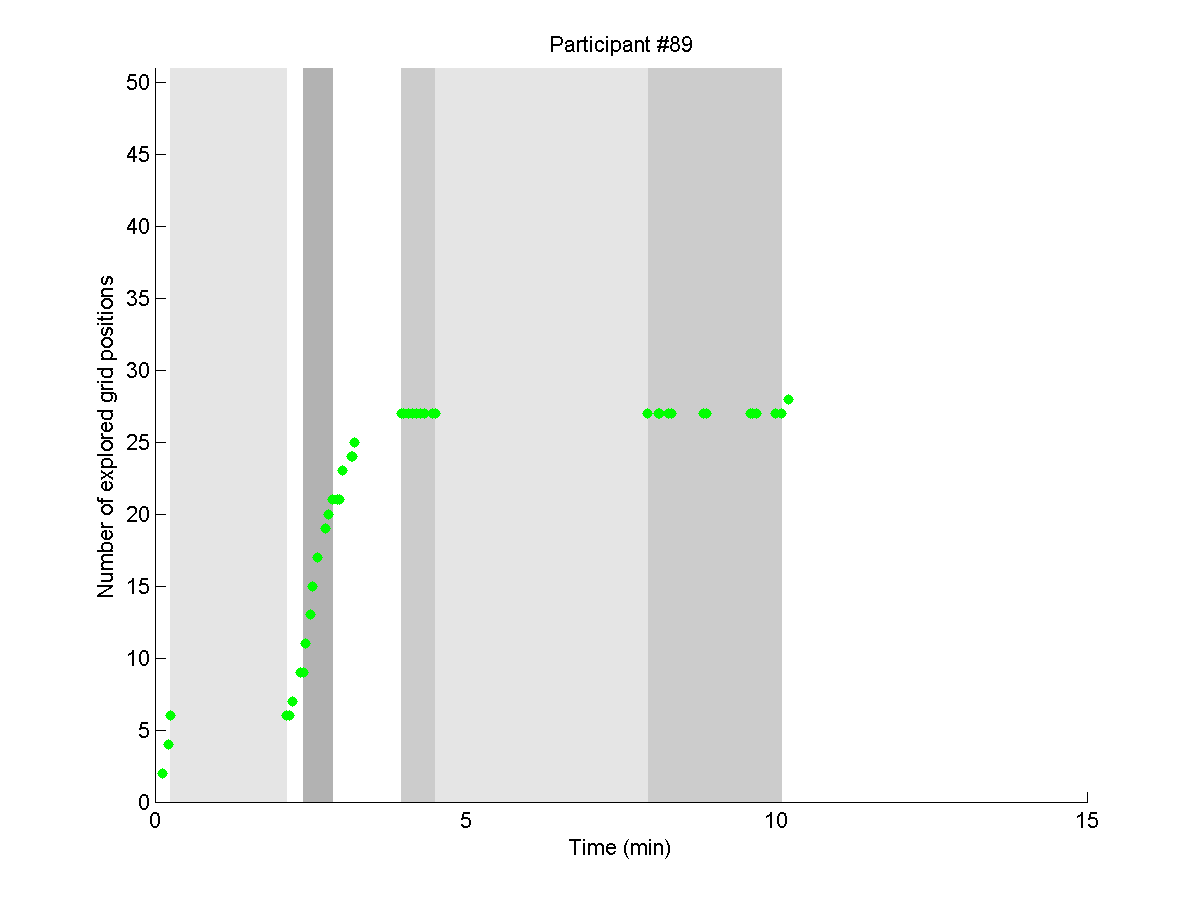

Supplement: Supplementary file 1 [file Presentation1.ZIP › individual plots/89.png]

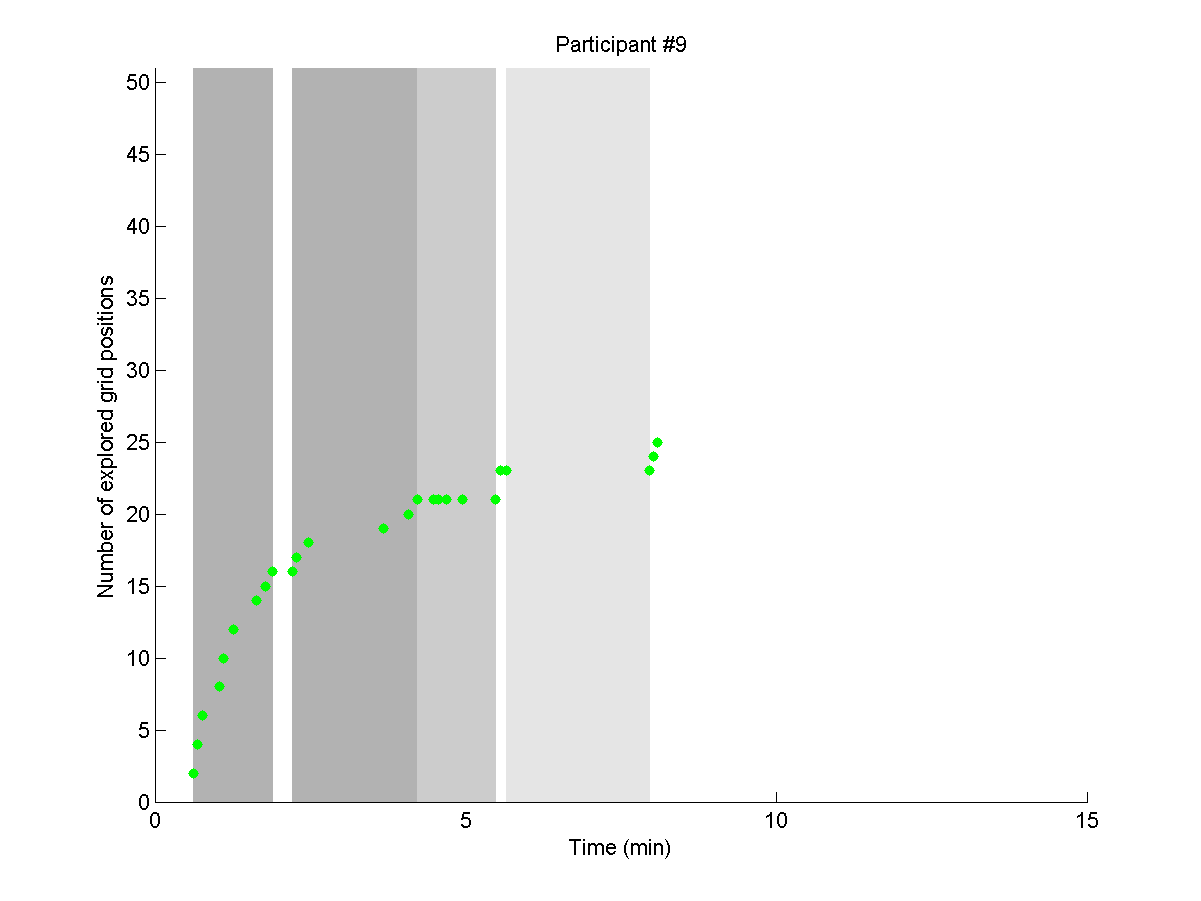

Supplement: Supplementary file 1 [file Presentation1.ZIP › individual plots/9.png]

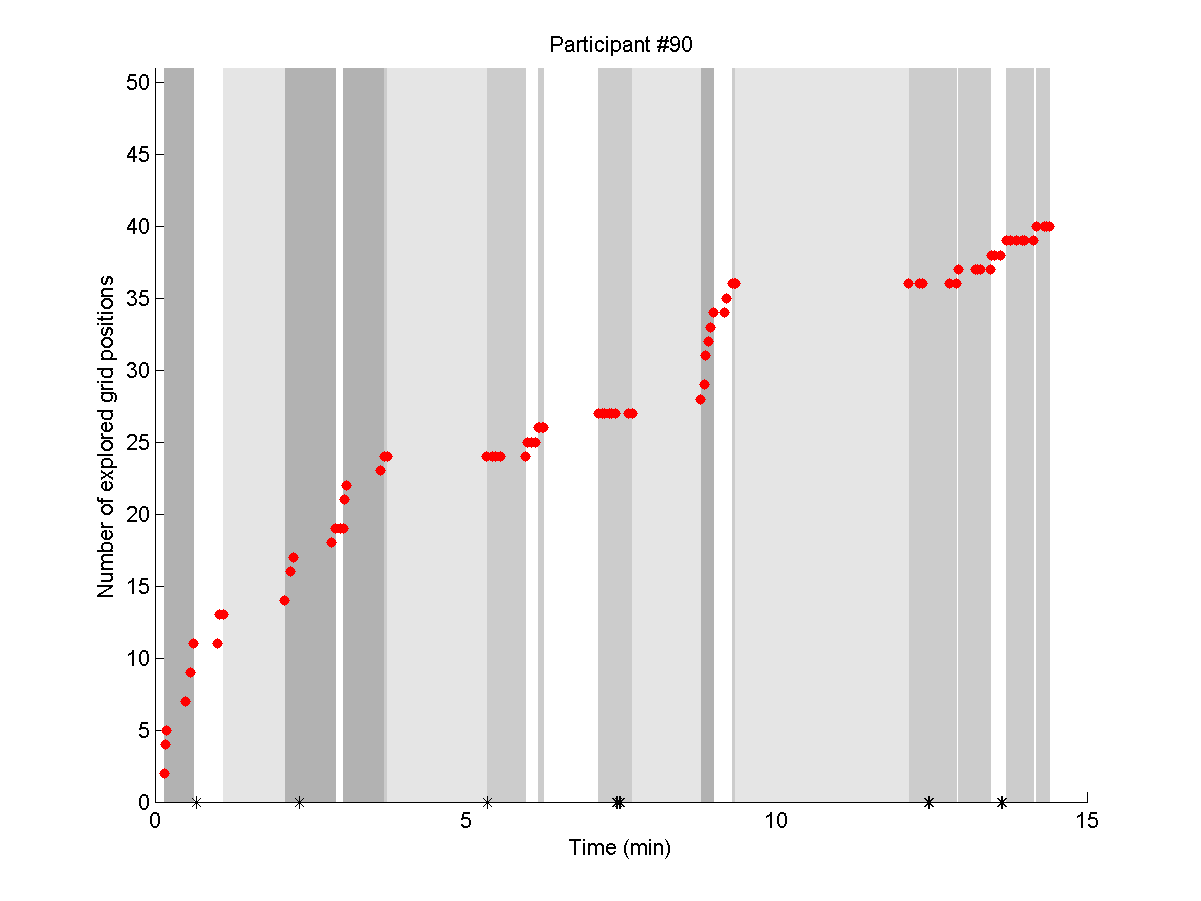

Supplement: Supplementary file 1 [file Presentation1.ZIP › individual plots/90.png]

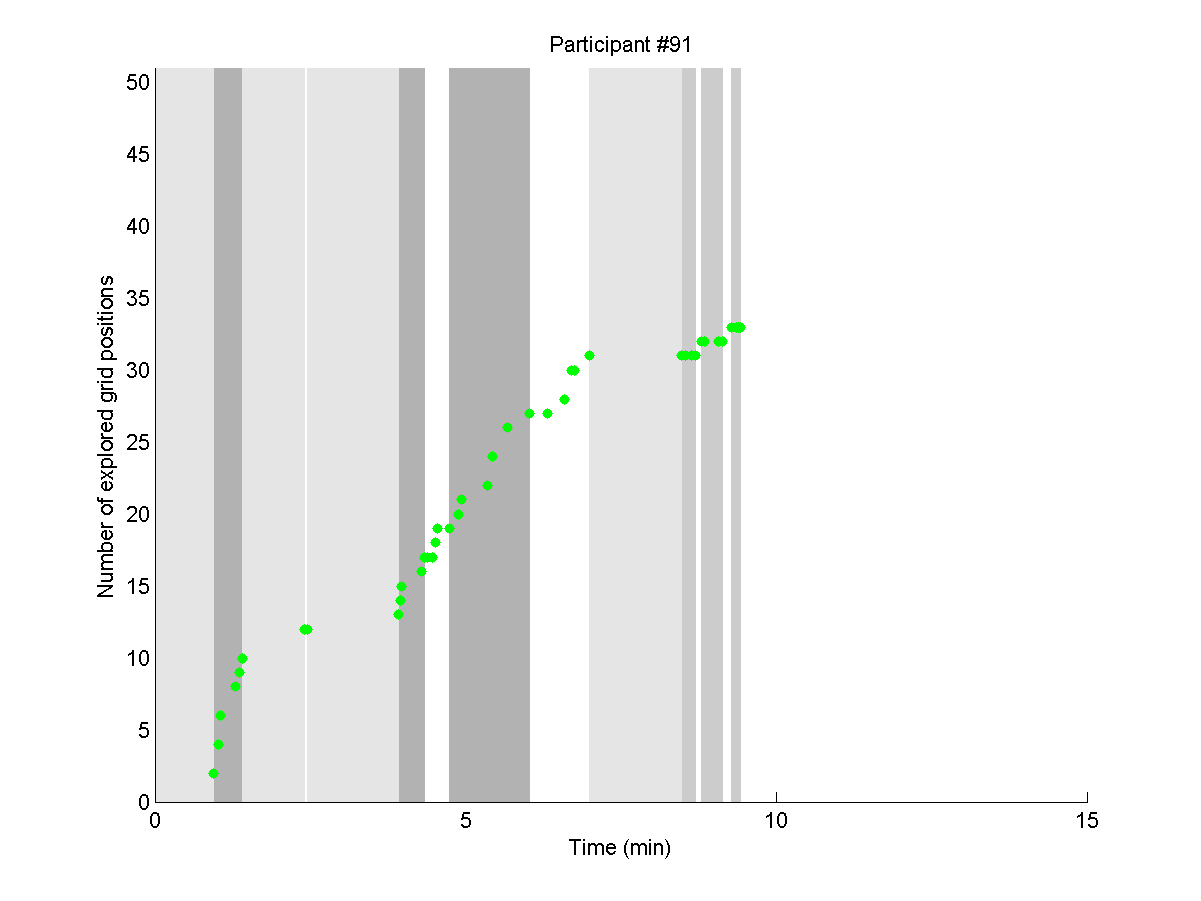

Supplement: Supplementary file 1 [file Presentation1.ZIP › individual plots/91.png]

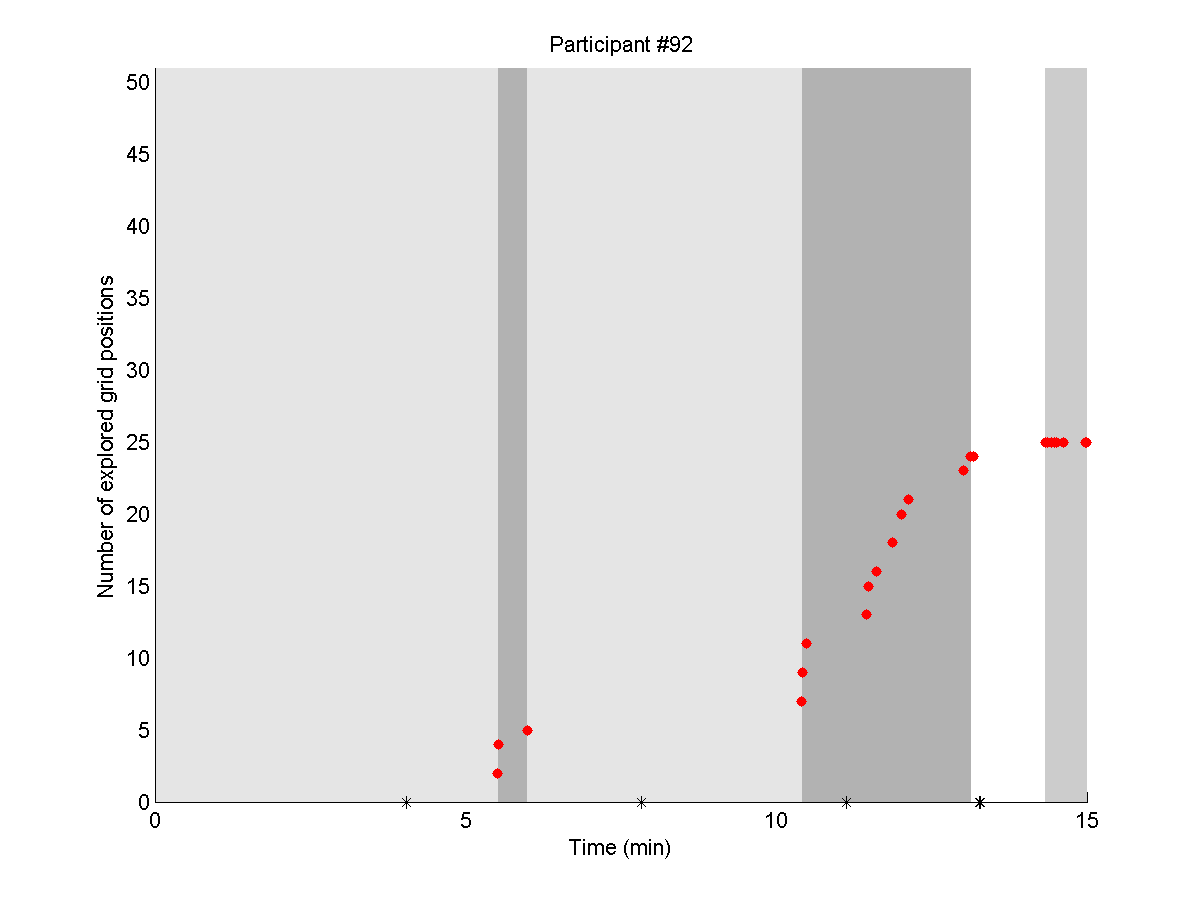

Supplement: Supplementary file 1 [file Presentation1.ZIP › individual plots/92.png]

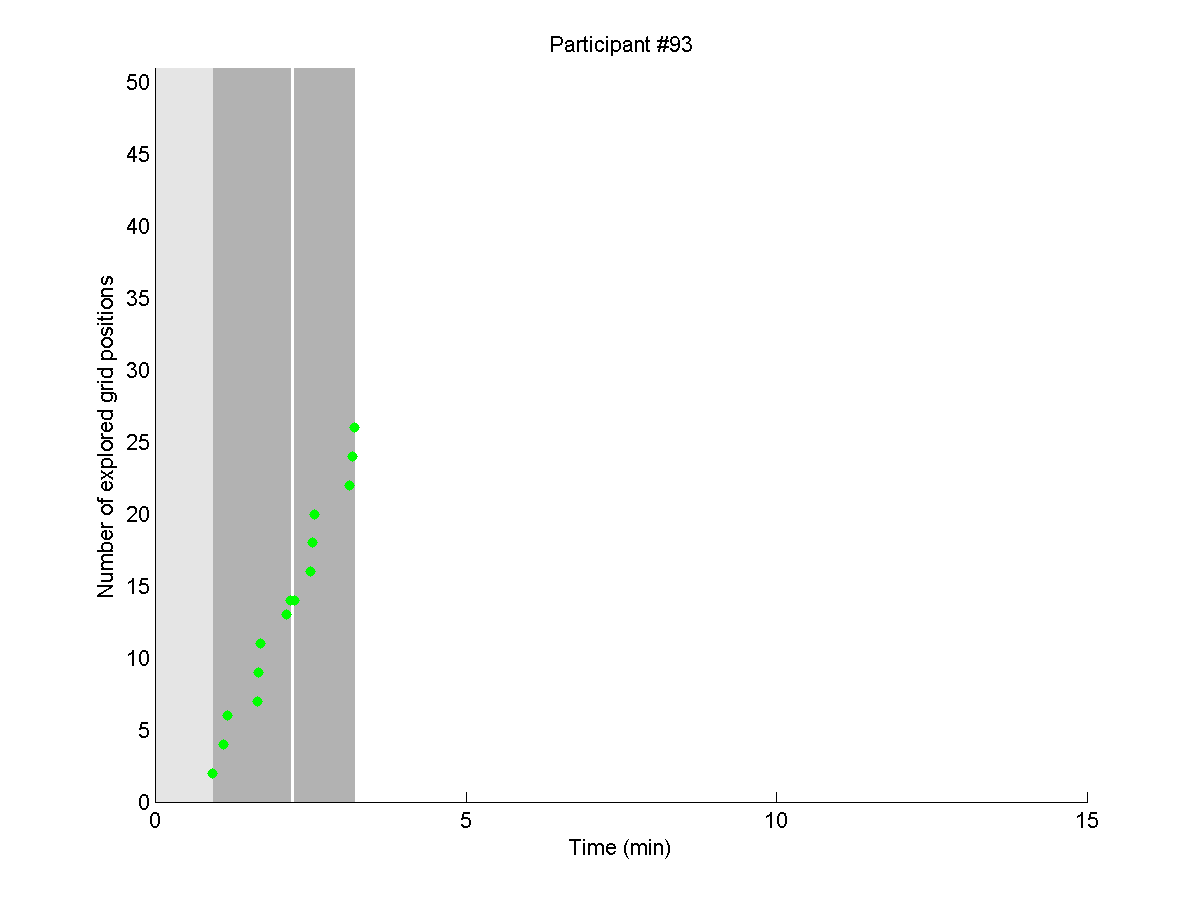

Supplement: Supplementary file 1 [file Presentation1.ZIP › individual plots/93.png]

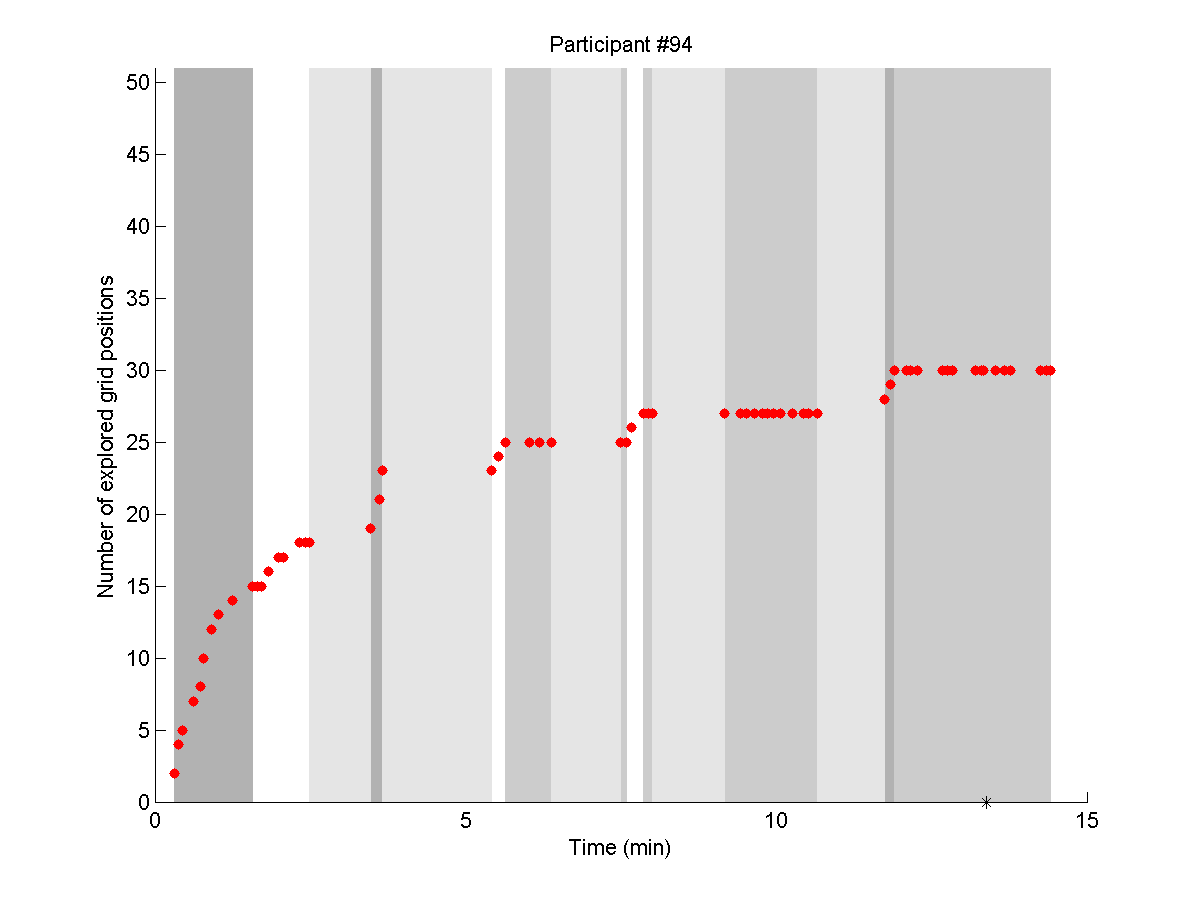

Supplement: Supplementary file 1 [file Presentation1.ZIP › individual plots/94.png]

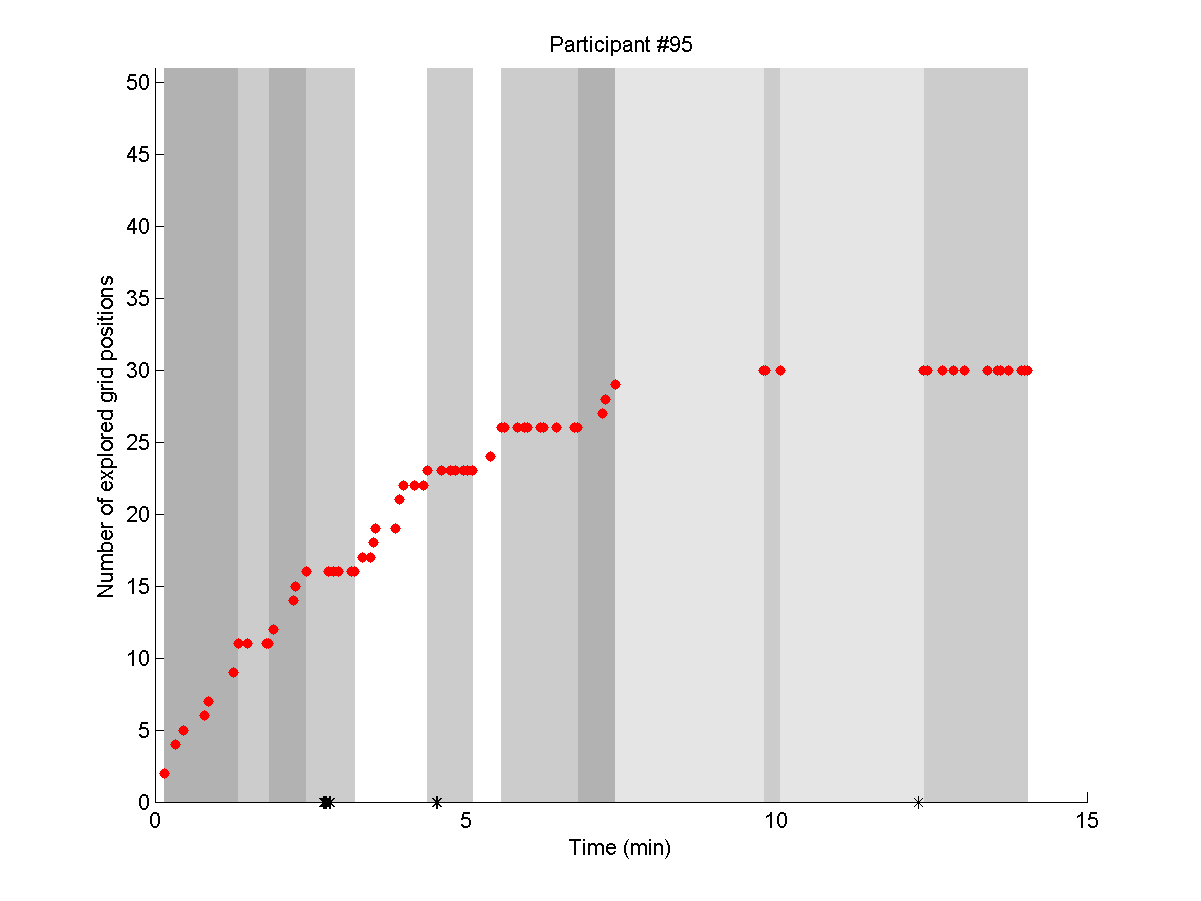

Supplement: Supplementary file 1 [file Presentation1.ZIP › individual plots/95.png]

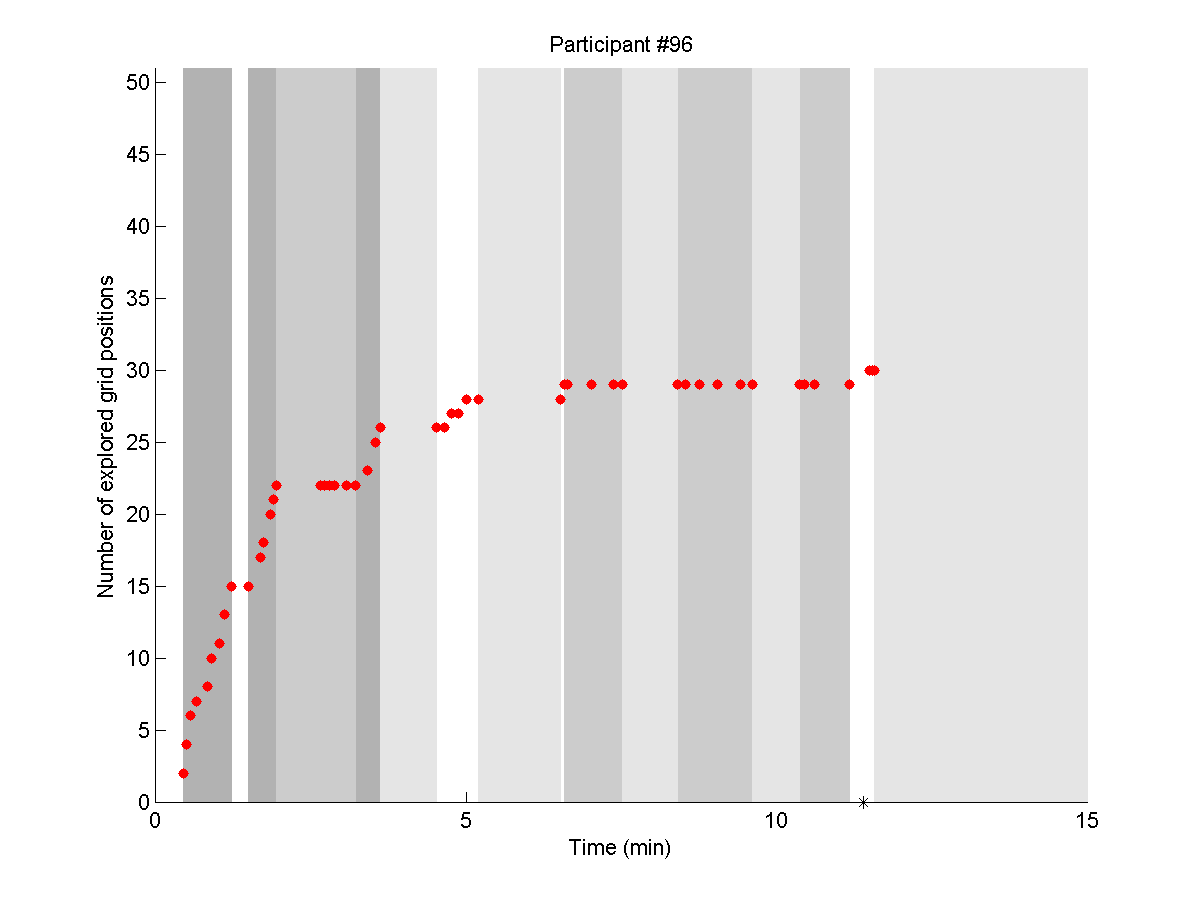

Supplement: Supplementary file 1 [file Presentation1.ZIP › individual plots/96.png]

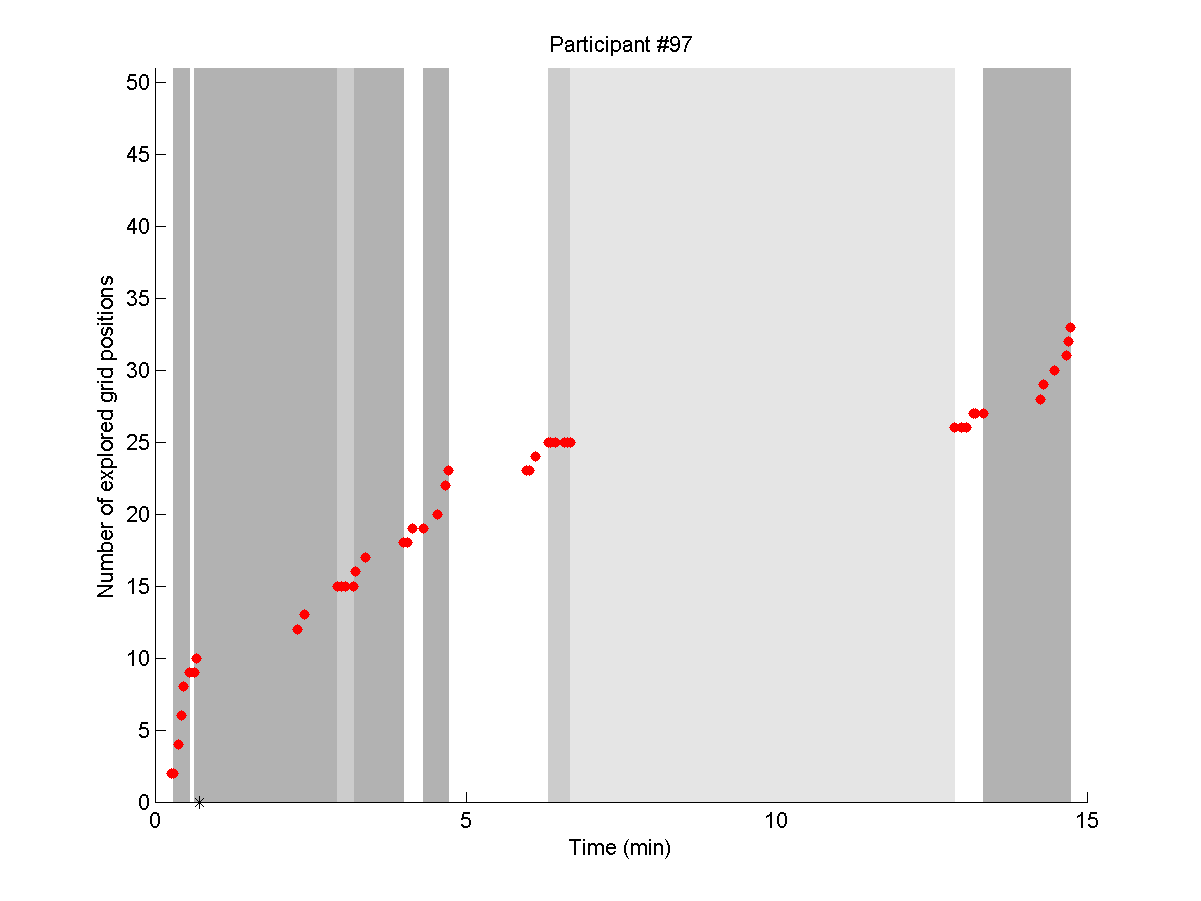

Supplement: Supplementary file 1 [file Presentation1.ZIP › individual plots/97.png]

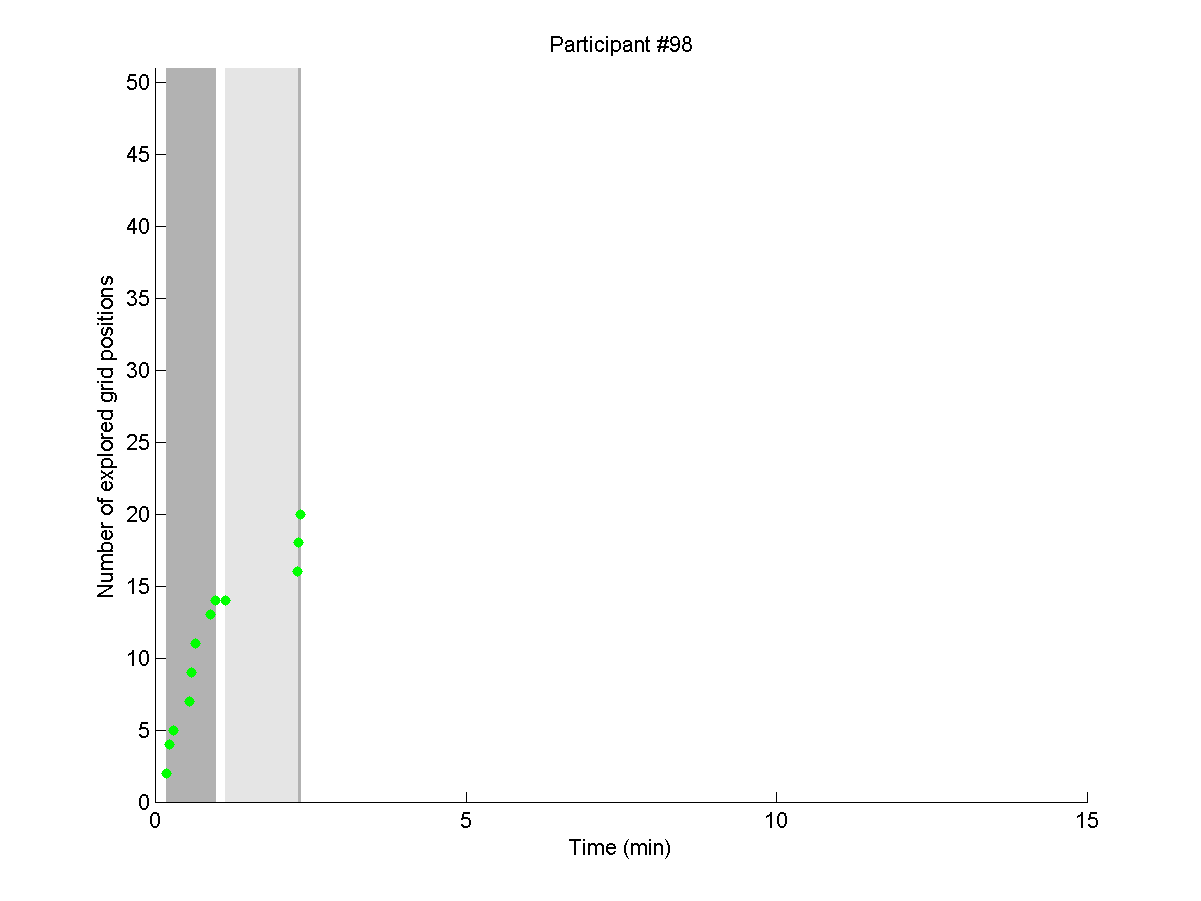

Supplement: Supplementary file 1 [file Presentation1.ZIP › individual plots/98.png]
